# Novel pyrrolo[2,1-*a*]isoquinoline aryl ketones attenuate carbon nanotube-induced acute lung injury through NF-κB pathway inhibition

**DOI:** 10.1039/d5ra05055c

**Published:** 2025-11-06

**Authors:** Guo-Liang Qiu, Jing Zhang, Zongze Yao, Wei Shao, Jiahong Ma, Hua-Li Qin, Wenjian Tang

**Affiliations:** a Department of Pharmacy, Anhui No. 2 Provincial People's Hospital Hefei 230041 China; b Anhui Province Key Laboratory of Occupational Health, Anhui No. 2 Provincial People's Hospital Hefei 230041 China; c School of Chemistry, Chemical Engineering and Life Science, Wuhan University of Technology Wuhan 430070 China qinhuali@whut.edu.cn ahmupharm@126.com; d School of Pharmacy, Anhui Medical University Hefei 230032 China

## Abstract

Acute lung injury (ALI), a life-threatening inflammatory disorder characterized by disrupted gas exchange and high mortality, urgently requires novel therapeutic strategies. Herein, anti-inflammatory activity of a series of pyrrolo[2,1-*a*]isoquinoline aryl ketones against carbon nanotube-induced ALI was evaluated, along with their mechanism. Sixteen aryl ketone derivatives incorporating a pyrrolo[2,1-*a*]isoquinoline scaffold were evaluated for their anti-inflammatory potential in RAW264.7 macrophage cells. Among them, 3g bearing 4-F-phenyl and 3k with 2-Me-phenyl demonstrated notably potent inhibitory effects on the LPS-induced release of nitric oxide (NO) in RAW264.7 macrophages, outperforming betulinic acid (IC_50_ values: 3g = 6.91 μM, 3k = 10.10 μM, betulinic acid = 11.89 μM). Both compounds exhibited a dose-dependent suppression of TNF-α secretion, with IC_50_ values of 7.85 μM and 8.30 μM for 3g and 3k, Furthermore, they effectively mitigated histopathological lung damage in SWCNT-exposed mice. Mechanistic studies revealed that 3g and 3k effectively diminished the activation of NF-κB through the inhibition of IκB phosphorylation and the subsequent blockade of p65 nuclear translocation. These results identify pyrrolo[2,1-*a*]isoquinoline aryl ketones as potential therapeutic agents capable of attenuating SWCNT-induced pulmonary inflammation through modulation of the NF-κB signaling pathway modulation.

## Abstract 2

Novel pyrrolo[2,1-*a*]isoquinoline aryl ketones 3g and 3k represent promising anti-inflammatory candidates for SWCNT-induced pulmonary injury, functioning through NF-κB pathway regulation.

## Graphical abstract

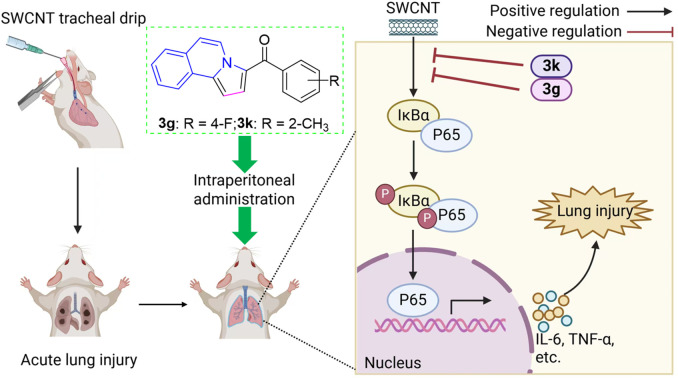

## 1. Introduction

Acute lung injury (ALI) manifests as damage to the alveolar epithelial cells and lung capillary endothelial cells, accompanied by elevated alveolar-capillary permeability and compromised gas exchange function. This pathological condition may arise from a multitude of direct and indirect etiological factors, encompassing severe infections, shock states, septicemia, burns injuries, traumatic events, and poisoning incidents. The hallmarks of ALI encompass inflammation, oxidative stress, neutrophil infiltration, *etc.*^1–3^ When inflammation remains unchecked, it has the potential to progress into acute respiratory distress syndrome (ARDS), in the final analysis, result in multi-organ dysfunction.^2^ In clinical practice, ALI presents a formidable, life-threatening challenge, marked by notably high rates of morbidity and mortality.^4^ To date, current therapeutic approaches have demonstrated limited efficacy in mitigating both the mortality rate and incidence of this debilitating disease.^5^

Carbon nanotubes (CNTs) represent a category of nanomaterials that find a diverse array of applications across both industrial and consumer sectors, spanning from rechargeable batteries to medical devices and drug delivery systems.^6,7^ However, due to their exceedingly small size, fibrous morphology, and high degree of bio-persistence, CNT-respirable fibers can act as foreign entities when inhaled. These fibers possess the potential to induce a spectrum of toxic effects, encompassing the onset of pathological conditions such as inflammation and fibrosis.^8^ Nevertheless, anti-inflammatory have demonstrated a high degree of efficacy in mitigating CNT-induced ALI. Thus, the pursuit of novel anti-inflammatory compounds through drug screening continues to be a highly dynamic and actively researched area within the scientific community.^9–12^

**Fig. 1:**
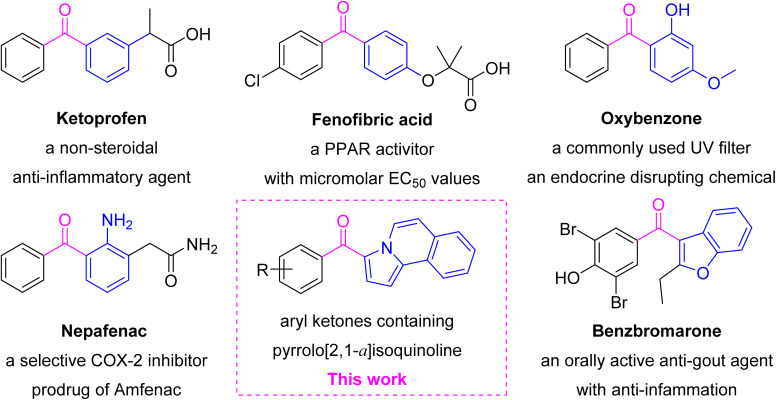
The fragment splicing of pyrrolo[2,1-*a*]isoquinoline aryl ketones as anti-inflammatory agents in this study.

Diaryl ketones, a privileged structural motif in medicinal chemistry, are valuable building blocks widely present in natural products and therapeutic agents (Fig. 1). Ketoprofen, a non-steroidal anti-inflammatory drug (NSAID), inhibits the cyclooxygenase activity (IC_50_ = 2 nM for COX-1 and 26 nM for COX-2).^13,14^ Fenofibric acid not only is a PPAR activator (EC_50_ = 22.4, 1.47, and 1.06 μM for PPAR-α, -γ and -δ, respectively), but also has COX-2 inhibitory activity (IC_50_ = 48 nM).^15^ Oxybenzone, an endocrine-disrupting chemical, can disrupt retinoid X receptor signaling, alter epigenetic status, and impair autophagy in apoptotic neuronal cells.^16^ Nepafenac as a selective COX-2 inhibitor is routinely used in ophthalmology to control pain following cataract surgery.^17^ Benzbromarone as an oral anti-gout agent has anti-inflammatory, anti-oxidative stress and nephroprotective effects.^18^ NSAIDs are the powerful armor against COX-2- and NF-κB-mediated inflammation, and many are inhibitors of NF-κB pathway.^19^ Therefore, it is meaningful for finding new structural motifs containing aryl ketone as anti-inflammatory agents.^20–22^

Pyrrolo[2,1-*a*]isoquinoline combining isoquinoline and pyrrole is a kind of scaffold in a lot of bioactive natural molecules and pharmaceutically important materials, such as the well-known lamellarin alkaloids derived from marine invertebrates, exhibiting prominent antitumor, antiviral, antibacterial and anti-oxidant activities.^23–25^ Viewing the significance of diaryl ketone as a privileged motifs in medicinal chemistry, pyrrolo[2,1-*a*]isoquinoline unit would be introduced into aryl ketone to construct one new molecule (Fig. 1). (*E*)-2-Methoxyethene-1-sulfonyl fluoride (MESF) as a sulfonyl fluoride reagent was used to construct bioactive molecules.^26,27^ A class of unique aryl ketones containing a pyrrolo[2,1-*a*]isoquinoline motif was synthesized using an acetylene substitute for [3 + 2] cycloaddition between MESF and isoquinolinium *N*-ylides. In this study, structurally unique chemicals containing pyrrolo[2,1-*a*]isoquinoline ketone motifs were studied for the anti-inflammatory activity and preliminary mechanisms.

## 2. Results and discussion

### 2.1. Chemistry

**Scheme 1:**
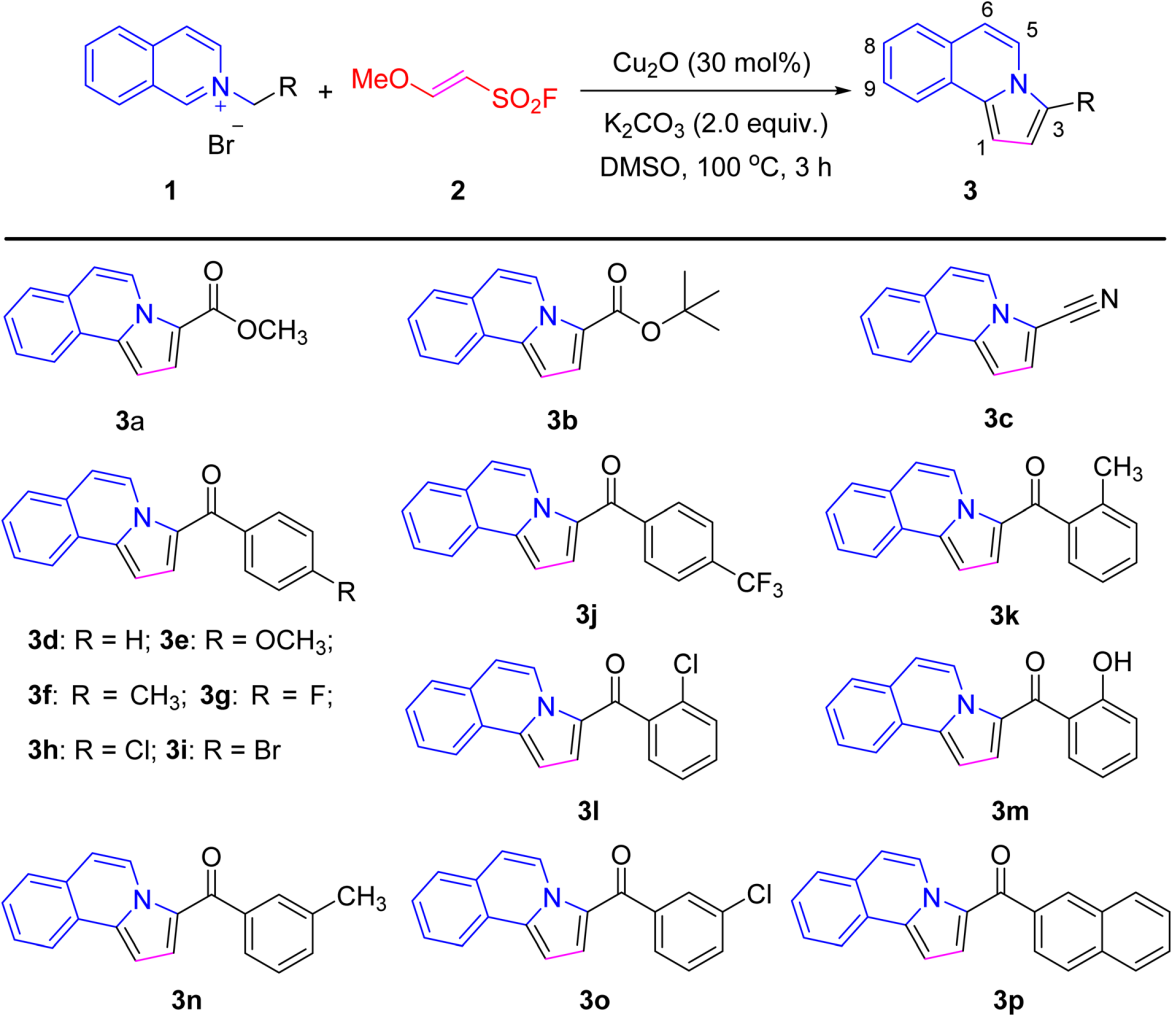
Chemical structures of pyrrolo[2,1-*a*]isoquinoline derivatives 3a–3p.

Recently, a series of pyrrolo[2,1-*a*]isoquinoline aryl ketones were synthesized using an acetylene substitute for the [3 + 2] cycloaddition between MESF and isoquinolinium *N*-ylides (Scheme 1). The [3 + 2] cycloaddition intermediate was generated by the reaction isoquinolinium *N*-ylide (1) and MESF (2) *via* a 1,3-dipolar cycloaddition reaction. The reaction process included an oxidative electron-transfer and subsequent sulfonyl fluoride and methoxy's elimination. Under basic and high-temperature conditions, the desired product pyrrolo[2,1-*a*]isoquinoline aryl ketones 3a–3p in 32–78% yield.^27^

### 2.2. Cytotoxicity evaluation

The toxic effect of synthesized compounds was evaluated on RAW264.7 cells utilizing a CCK8 assay kit. At an identical concentration of 50 μM, most compounds displayed a minimal impact on cell viability, indicating the low toxicity of tested compounds (Table 1). The results showed that most of tested compounds had survival rates that exceeded 80%. Therefore, compounds at this concentration proved to be appropriate for subsequent biological evaluation, excluding only those exhibiting the highest cytotoxicity.

**Table 1:** Effect of compounds on the activity of RAW264.7 cells

Compd	Cell viability^a^ (%)	Compd	Cell viability^a^ (%)
Control	100	3a	92.4 ± 6.3
3b	95.6 ± 2.9	3c	97.1 ± 2.6
3d	96.8 ± 6.6	3e	88.7 ± 5.8
3f	53.6 ± 6.4	3g	86.5 ± 1.2
3h	100.8 ± 3.0	3i	95.9 ± 7.7
3j	89.9 ± 5.6	3k	98.8 ± 4.7
3l	99.3 ± 8.8	3m	46.4 ± 6.5
3o	91.5 ± 8.5	3p	93.2 ± 4.7
Betulinic acid	85.8 ± 7.4		

a The results were shown as means ± SD (*n* = 3).

### 2.3. Anti-inflammatory activity

The severe inflammatory response associated with ALI has led to its notably high ates of both mortality and morbidity. Despite extensive research efforts focused on the treatment ALI, no specific drugs have been identified thus far. In the quest to discover innovative anti-inflammatory drugs for ALI treatment, we assessed the anti-inflammatory potential of pyrrolo[2,1-*a*]isoquinoline aryl ketones were evaluated through their capacity to suppress LPS-induced NO release in RAW264.7 cells.^21,28^ Griess reagent was used to quantify the levels of LPS-induced NO release in RAW264.7 cells following treatment with the synthesized compounds. Compared to the control group, a notably elevated release of NO was observed subsequent to the stimulation of RAW264.7 cells with LPS. When cells were subjected to pretreatment with the tested compounds at varying concentrations, a significant reduction was observed in the LPS-induced production of NO within the cell supernatant. As shown in Table 2, the majority of compounds exhibited anti-inflammatory activity, with compounds 3k and 3g significantly reducing LPS-induced NO release, an effect superior to that of betulinic acid (IC_50_ values of 6.91, 10.10 and 11.89 μM for 3g, 3k and betulinic acid, respectively).

**Table 2:** Inhibition for LPS-induced NO production in RAW 264.7 cells^d^

Compound	IC_50_^a^^,^^b^ (μM) or inhibition rate^a^^,^^b^^,^^c^ (%)	Compound	IC_50_^a^^,^^b^ (μM) or inhibition rate a^,^^b^^,^^c^ (%)
3a	19.90 ± 0.71	3b	42.0 ± 4.2%
3c	46.9 ± 1.8%	3d	48.0 ± 1.3%
3e	na	3f	tox
3g	6.91 ± 0.12	3h	39.6 ± 5.3%
3i	na	3j	na
3k	10.10 ± 2.09	3l	38.2 ± 5.1%
3m	tox	3n	46.8 ± 3.1%
3o	39.2 ± 7.1%	3p	na
Betulinic acid	11.89 ± 1.30		

a The results were shown as means ± SD (*n* = 3).

b NO production was detected using Griess reagent.

c The NO inhibition rates were measured at the 40 mM concentration.

d na = no activity at the concentration of 40 μM; tox = toxicity.

For pyrrolo[2,1-*a*]isoquinoline aryl ketones, the nature and positional distribution of substituents on the phenyl moiety significantly influence their anti-inflammatory activity. The structure–activity relationship (SAR) was as followed: (i) for the pyrrolo[2,1-*a*]isoquinoline-3-carboxylates, the anti-inflammatory activity was affected by the ester group (3a for –OCH_3_ > 3b for –*O-t*-butyl); (ii) the substituent of phenyl ring affected the activity, for example, 4-F-phenyl showed the most activity (3g, IC_50_ = 6.91 μM), IC_50_ of 2-Me-phenyl (3k) was 10.10 μM, compounds 3h, 3l and 3o with Cl substituent also had good activity; (iii) the position of substituent of phenyl ring affected the activity, for example, 3k (*o*-CH_3_) > 3n (*m*-CH_3_) for the methyl substituent, 3h (*p*-Cl) ≈ 3l (*o*-Cl) ≈ 3o (*m*-Cl) for the chloro-substituent (Table 2). Combined with the cytotoxicity results (Table 1), the active compounds 3g and 3k were chosen for subsequent experiments.

### 2.4. Effects of compounds 3g and 3k on TNF-α and IL-6

**Fig. 2:**
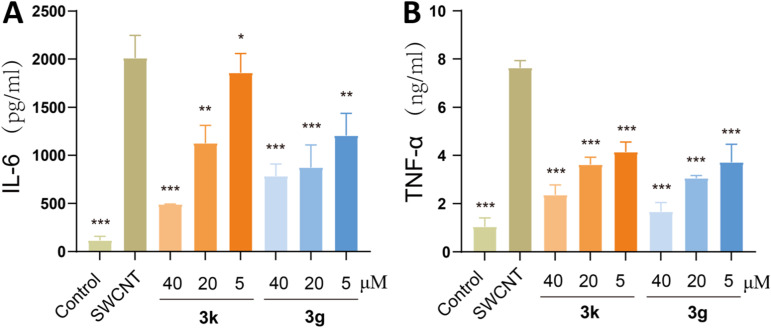
Compounds 3g and 3k inhibited the cytokine production of RAW264.7 cells stimulated by LPS. RAW264.7 cells were pretreated with 3g and 3k, at concentrations of 5, 20, 40 μM for 1 h, then incubated with LPS (0.5 mg mL^−1^) for 24 h. The levels of IL-6 (A) and TNF-α (B) in the culture medium were measured by ELISA kits. Data are expressed as mean ± SEM for each group of independent experiments (*n* = 3); *p < 0.05; **p < 0.01; ***p < 0.001 *vs.* model group.

To elucidate the anti-inflammatory mechanisms underlying 3g and 3k, we conducted a comprehensive investigation into their impact on the secretion of pro-inflammatory cytokines, employing an LPS-stimulated RAW264.7 macrophage model. TNF-α and IL-6, which serve as pivotal mediators in the pathogenesis of ALI, are primarily secreted by activated alveolar macrophages, neutrophils, and lung structural cells. Notably, elevated levels of these cytokines exhibit a strong correlation with deteriorated clinical outcomes in patients suffering from ALI.^29–31^ In this study, RAW264.7 cells were treated with LPS (0.5 μg mL^−1^) and varying concentrations of 3g or 3k for 24 hours. ELISA analysis revealed a significant upregulation of TNF-α and IL-6 in LPS-stimulated cells compared to untreated controls (p < 0.001; Fig. 2). Both compounds suppressed cytokine secretion in a dose-dependent manner, with 3g exhibiting slightly higher potency than 3k (IC_50_ = 7.85 and 8.30 μM for TNF-α inhibition, respectively). These findings demonstrated that 3g with 4-F-phenyl and 3k with 2-Me-phenyl effectively mitigate inflammatory cascades through the inhibition of pro-inflammatory cytokine secretion, consequently, both alleviate exaggerated immune response and the resultant cellular damage. This mechanistic understanding provides robust support for the therapeutic potential of the novel diaryl ketone scaffold in the ALI management.

### 2.5. Results of *in vivo* animal experiments

#### 2.5.1. Complete blood count

**Fig. 3:**
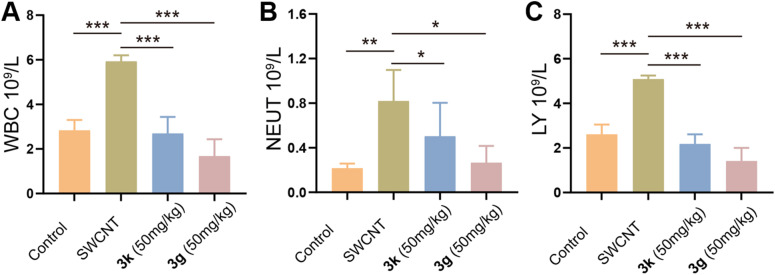
(A) White blood cell count; (B) neutrophil granulocyte count; (C) lymphocyte count. Data are expressed as mean ± SEM for each group of independent experiments (*n* = 3); *p < 0.05; **p < 0.01; ***p < 0.001 *vs.* model group.

To explore the immunomodulatory effects of 3g and 3k, we conducted an analysis of systemic inflammatory responses in mice with SWCNT-induced ALI by employing hematological profiling. During inflammatory responses, immune activation induces cytokine production and modulates leukocyte trafficking dynamics. Notably, neutrophil (NEUT) accumulation serves as a hallmark indicator of acute inflammation, whereas lymphocyte (LY) counts provide insights into systemic stress responses.^32,33^ Complete blood count (CBC) analysis revealed elevated LY levels in ALI model mice (*p* < 0.05 *vs.* control; Fig. 3), consistent with heightened immune activation. Notably, 3g or 3k treatment significantly reduced circulating LY populations (*p* < 0.01 *vs.* model), suggesting effective mitigation of pathological immune hyperactivity. Mechanistically, the proliferation of LY is initiated in response to inflammatory stimuli as a means to combat infection. The observed normalization of LY levels following treatment suggested that 3g and 3k can effectively attenuate inflammatory signaling pathways. Consequently, this leads to a diminished requirement for the continuous mobilization of LY. These findings highlight the anti-inflammatory and immunomodulatory properties of pyrrolo[2,1-*a*]isoquinoline derivatives in mitigating SWCNT-induced pulmonary injury.

#### 2.5.2. Histopathological changes in ALI mice

**Fig. 4:**
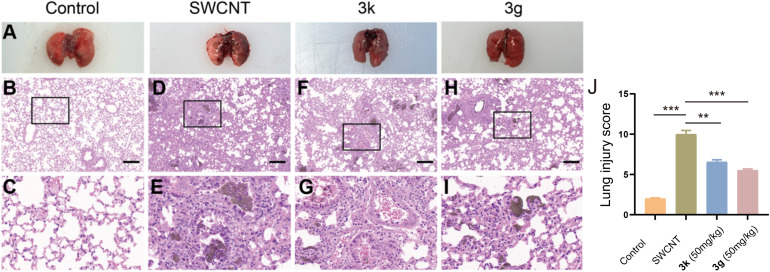
(A) Images of the lungs of the mice in each experimental group. Representative images of H&E staining: (B–I), (B) control group; (C) control group (×200); (D) model group; (E) model group (×200); (F) 3k treatment group; (G) 3k treatment group (×200); (H) 3g treatment group; (I) 3g treatment group (×200); (J) the histogram of lung injury scores. Data are expressed as mean ± SEM for each group of independent experiments (*n* = 3); *p < 0.05; **p < 0.01; ***p < 0.001 *vs.* model group.

To evaluate the histopathological impact of 3g and 3k on SWCNT-induced ALI, hematoxylin and eosin (H&E) staining was performed on murine lung tissues. As illustrated in Fig. 4A, the model group exhibited severe pulmonary edema, hemorrhage, and disrupted alveolar architecture compared to the histologically normal lungs of control mice (Fig. 4B and C). Pathological analysis revealed pronounced inflammatory cell infiltration, alveolar congestion, septal thickening, and interstitial neutrophil accumulation in the model group (Fig. 4D and E). Treatment with 3g and 3k significantly ameliorated these histopathological features, as evidenced by reduced tissue swelling, diminished congestion, and attenuated inflammatory cell recruitment (Fig. 4F–I). Notably, 3g demonstrated superior efficacy in mitigating structural damage and neutrophil infiltration compared to 3k, highlighting its potential as a lead therapeutic candidate.

#### 2.5.3. Effect of inflammatory factors in BALF

**Fig. 5:**
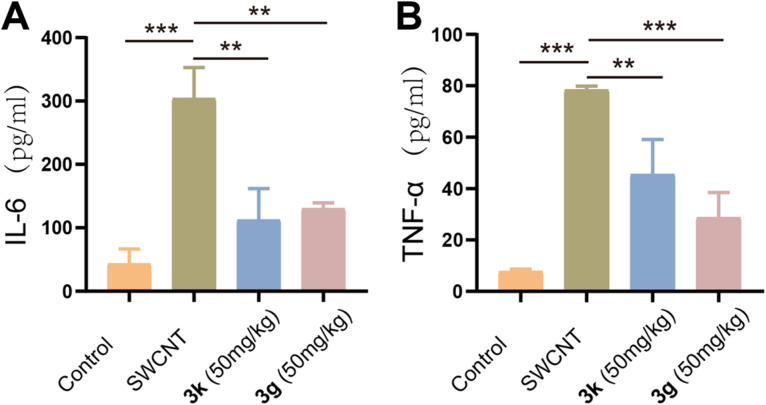
(A) IL-6 in BALF; (B) TNF-α in BALF. Data are expressed as mean ± SEM for each group of independent experiments (*n* = 3); *p < 0.05; **p < 0.01; ***p < 0.001 *vs.* model group.

It is well known that proinflammatory factors play a pivotal role in initiating inflammation during the initial stages of ALI, significantly exacerbating the severity of lung damage.^34^ To confirm the *in vivo* inhibitory effects of 3g and 3k, we examined the levels of pro-inflammatory cytokines in bronchoalveolar lavage fluid (BALF) from ALI mice. As shown in Fig. 5A and B, a significant elevation in the levels of TNF-α and IL-6 were observed in the BALF of model mice, in contrast to the control group. Notably, the administration of a 50 mg per kg dose of either 3g or 3k effectively suppressed the release of TNF-α and IL-6, thereby demonstrating the anti-inflammatory properties of pyrrolo[2,1-*a*]isoquinoline aryl ketones.

#### 2.5.4. Immunofluorescence results

**Fig. 6:**
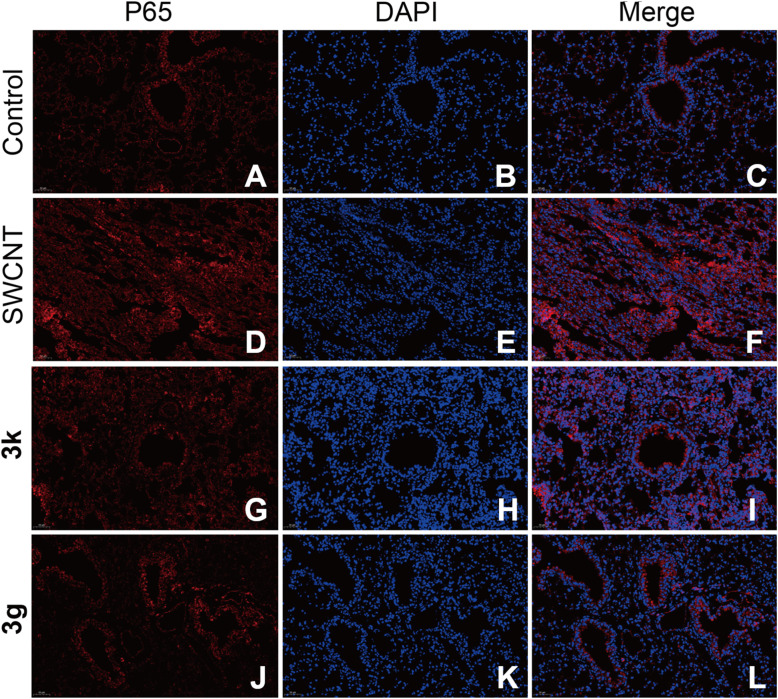
Immunofluorescence staining of mouse lung tissue in each group (200×). (A–C) control group; (D–F) model group; (G–I) 3k treatment group; (J–L) 3g treatment group; (A, D, G, J) p65 subunit staining (red); (B, E, H, K) DAPI staining (blue) indicating nuclei; (C, F, I, L) merged images demonstrating co-localization of p65 and DAPI signals.

To elucidate the mechanism underlying the modulation of NF-κB pathway, we employed immunofluorescence techniques to scrutinize the spatiotemporal dynamics of NF-κB p65 within lung tissues. The canonical NF-κB signaling cascade involves IκB kinase (IKK)-mediated phosphorylation of IκBα, triggering its ubiquitination and proteasomal degradation. This process releases the NF-κB p65/p50 heterodimer, enabling its nuclear translocation and subsequent initiation of pro-inflammatory mediator transcription.^35,36^ Immunofluorescence analysis revealed distinct subcellular localization patterns: in control group tissues, p65 exhibited predominant cytoplasmic retention (Fig. 6). Conversely, SWCNT-induced ALI mice displayed intensified p65 fluorescence with pronounced nuclear accumulation (*p* < 0.01 *vs.* control). Notably, both 3g or 3k treatments attenuated this pathological redistribution, as evidenced by reduced fluorescence intensity and suppressed nuclear translocation of p65. A comparative analysis further revealed that 3g exhibited significantly enhanced efficacy in inhibiting he nuclear translocation of p65 when compared to compared to 3k. This finding implies a pharmacological activity that is contingent upon the structural characteristics of the compounds.

#### 2.5.5. Effect on the NF-κB signaling pathway

The expression levels of phosphorylated IκB and p65 were meticulously examined. To further delve into and evaluate the impact of compounds 3g and 3k on the NF-κB signaling pathway within SWCNT-induced ALI murine models, western blotting analysis was used to comprehensively assess the activation status of the NF-κB pathway in lung tissues.

**Fig. 7:**
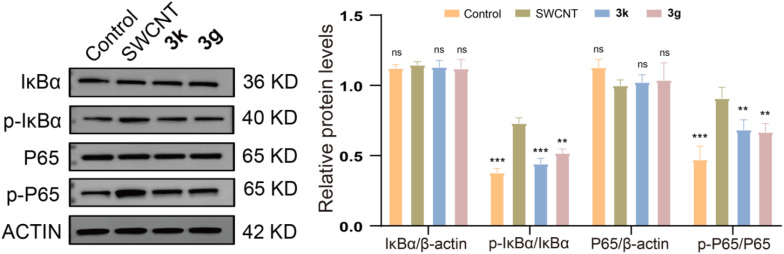
Compounds 3k and 3g inhibited SWCNT-induced activation of the NF-κB signaling pathway. Data are expressed as mean ± SEM for each group of independent experiments (*n* = 3); *p < 0.05; **p < 0.01; ***p < 0.001 *vs.* model group.

As shown in Fig. 7, the results demonstrated significant alterations in both expression and phosphorylation levels of NF-κB and its downstream regulators. Specifically, phosphorylated IκB and p65 levels were quantified, revealing a pronounced elevation in the model group compared to controls (*p* < 0.001). Notably, administration of 3g and 3k substantially suppressed these phosphorylation events, indicating effective modulation of NF-κB signaling. These findings suggested that 3g and 3k mitigate pulmonary inflammation by inhibiting NF-κB pathway activation, consequently attenuating pro-inflammatory cytokine release. Collectively, novel pyrrolo[2,1-*a*]isoquinoline aryl ketones represent promising anti-inflammatory candidates for SWCNT-induced pulmonary injury, functioning through NF-κB pathway regulation.

## 3. Conclusions

Through comprehensive approaches encompassing activity screening, structure–activity relationship (SAR) analysis, as well as animal and cellular investigations, pyrrolo[2,1-*a*]isoquinoline aryl ketone emerged as a novel scaffold for the development of anti-inflammatory drugs. Among them, 3g bearing a 4-F-phenyl and 3k with a 2-Me-phenyl showed notably potent anti-inflammatory activities, with IC_50_ values of 6.91 μM and 10.10 μM, respectively. Furthermore, 3g and 3k demonstrated a dose-dependent inhibition of LPS-induced TNF-α release. By suppressing the NF-κB pathway, 3g and 3k effectively mitigated excessive lung inflammation, consequently reducing the severity of ALI. Moreover, *in vivo* administration of 3g and 3k significantly mitigated carbon nanotube-induced ALI by effectively suppressing inflammatory responses. Thus, pyrrolo[2,1-*a*]isoquinoline aryl ketones emerge as highly promising scaffolds for the development of innovative anti-inflammatory therapeutics, demonstrating significant potential for the treatment of ALI.

## 4. Materials and methods

### 4.1. Chemistry

As shown in Scheme 1, a series of pyrrolo[2,1-*a*]isoquinoline phenyl ketones were synthesized by an acetylene substitute for [3 + 2] cycloaddition between MESF and isoquinolinium *N*-ylides in recent work.^26^ An oven reaction tube equipped with a magnetic stirring bar and drying tube was charged with isoquinolinium *N*-ylides (1, 1.0 mmol), K_2_CO_3_ (276 mg, 2.0 mmol, 2.0 equiv.), Cu_2_O (30 mol%, 43 mg), DMSO (5.0 mL) and MESF (3.0 mmol, 3.0 equiv., 420 mg). Then, the reaction mixture was stirred for 3 h at a temperature of 100 °C. Once the reaction was complete, the mixture was extracted thrice with ethyl acetate (3 × 20 mL), the organic phase was washed with brine (20 mL), and dried over anhydrous Na_2_SO_4_. The solvent was vaporized in vacuum and the residue was further purified by silica gel chromatography (eluent: a mixture of petroleum ether, CH_2_Cl_2_ and EtOAc) to afford the title product 3a–3p. The purity (relative content) of all compounds was determined by HPLC through area normalization method. All reaction materials and equipment, the ^1^H-NMR, ^13^C-NMR, HRMS and HPLC data and spectra for 3a–3p are listed in the SI.

### 4.2. Cell culture and treatment

The murine macrophage RAW264.7 cell line was obtained from the BeNa Culture Collection company. RAW264.7 cells were meticulously cultured in DMEM supplemented with 10% fetal bovine serum, 100 μg per mL streptomycin, and 100 units per mL penicillin. Cells were maintained at 37 °C in a humidified incubator containing 5% CO_2_.

### 4.3. Cell activity assay

RAW264.7 cells were seeded in 96-well plates and incubated with various compounds, each at a uniform concentration of 50 μM, for 24 h. Following this incubation, CCK8 was added to the 96-well plates and incubated at 37 °C for 1 h. The control group was cultivated solely in fresh medium. The absorbance at 450 nm was quantified using an enzyme marker.

Blank: cultured in fresh medium only.

Compound: treated with compounds or lipopolysaccharide (LPS).

### 4.4. Assay for NO production

The quantification of NO production in activated RAW264.7 cells was achieved by measuring the concentration of nitrite, which serves as a stable oxidative metabolite of NO, employing a previously established protocol.^37^ RAW264.7 cells were cultured overnight in 48-well plates at a cellular density of 5 × 10^4^ cells per milliliter. After exposure to various concentrations of the test compounds (6.25, 12.5, 25, 50, and 100 μM) for 1 h, cells were treated with LPS at a concentration of 500 ng mL^−1^ for an additional 24 h. Briefly, Griess reagents I and II were mixed with 50 μL of the culture medium supernatant, and the resultant mixture was analyzed spectrophotometrically at a wavelength of 540 nm using an automated microplate reader. The NO inhibition rate was calculated using the following formula: NO inhibition rate = [(OD_540_ − compound OD540)/(OD_540_ − blank OD540)] × 100%.

Control: treated with LPS only.

Compound: treated with LPS and compounds.

Blank: cultured with fresh medium only.

### 4.5. Determination of TNF-α and IL-6 concentrations

Based on the initial screening results, compounds 3g and 3k were selected for further experimentation. RAW264.7 cells were cultured in 24-well plates for 24 h. Cells were pretreated with 3g and 3k (5, 20, or 40 μM) for 1 h, followed by stimulation with LPS (0.5 μg mL^−1^) for another 24 h. The supernatant was collected for analysis. The levels of TNF-α and IL-6 released into the macrophage supernatant were quantified using ELISA kits sourced from Elabscience (Wuhan, China), according to the manufacturer's guidelines.

### 4.6. Experimental animals

Male C57BL/6 mice, weighing 18–22 g, were obtained from Charles River Co., Ltd (Beijing, China). Male mice can avoid potential interference from the estrogen cycle on metabolic and immune responses, reduce inter-individual variability, and enhance data stability, making them particularly suitable for preliminary mechanistic exploration studies. The animals were housed under controlled room temperature conditions and maintained on a regular 12:12-hour light/dark cycle, with ad libitum access to food and water. Prior to experimental use, the mice were acclimatized to the laboratory environment for a minimum of seven days. All animal experiments were conducted in accordance with the guidelines approved by the Institutional Animal Care and Use Committee of Anhui Medical University (Approval No. LLSC20240585).

Based on the results of the previous *in vitro* experiments, 3g and 3k were chosen for animal experiments. 3g was dissolved in 5% DMSO and 3k was dissolved in 0.5% sodium carboxymethyl cellulose (CMC-Na). Male C57BL/6 mice were randomly divided into four groups of ten animals each: CON, single-walled carbon nanotubes (SWCNT) model, 3k (50 mg kg^−1^) + SWCNT, 3g (50 mg kg^−1^) + SWCNT. The SWCNT model group was administered a one-time tracheal drip of 50 μL of SWCNT suspension containing 40 μg of SWCNT (approximately 1.86 mg per kg body weight) per mouse to model ALI. A blank tracheal drip of PBS was administered to the negative control. 3k + SWCNT and 3g + SWCNT groups were administered 3k or 3g of 50 mg per kg body weight once a day for 3d. The animals in the CON group received the same volume of 0.5% CMC-Na. The 1st dose was administered 1 h before modeling to avoid excessive damage to mice. The control and model groups were injected intraperitoneally with a blank solvent. The mice were euthanized on the last day, and bronchoalveolar lavage fluid (BALF), blood, and lung tissue samples were collected for further analysis.

All procedures were conducted under anesthesia to ensure minimal suffering and to reduce the number of animals used. No unexpected deaths were observed during the experiment.

### 4.7. Complete blood count

Blood samples were extracted from the eyeballs of each experimental mouse cohort to obtain comprehensive whole blood specimens. Complete blood count (CBC) and differential leukocyte count (DLC) were determined using an automatic blood cell analyzer (Mindray CAL 6000, China).

### 4.8. BALF analysis

The collected bronchoalveolar lavage fluid (BALF) was centrifuged to obtain the supernatant. The concentrations of TNF-α and IL-6 in BALF were determined using ELISA. The ELISA kits used for this purpose were sourced from Elabscience (Wuhan, China). The entire procedure adhered strictly to the instructions provided by the reagent company.

### 4.9. Histopathologic evaluation

Mouse lung tissues were isolated and stored in a 4% paraformaldehyde solution. Pathological tissues were observed using hematoxylin and eosin (HE) staining. In addition, immunofluorescence (IFA) was performed to assess the expression of the NF-κB pathway p65. Representative images of each sample were acquired (magnification ×200) and analyzed using the ImageJ software. Using the Matute-Bello scoring system, we performed blinded acute lung injury scoring and generated a lung injury score histogram.

### 4.10. Western blot analysis

Lung tissue samples were collected from each mouse. Total protein was extracted from lung tissues using RIPA lysis buffer. The protein concentration was accurately determined using the BCA method. Protein samples were separated by 8% sodium dodecyl sulfate-polyacrylamide gel electrophoresis (SDS-PAGE) and transferred onto nitrocellulose membranes. The primary antibody was incubated at 4 °C overnight, followed by incubation with a secondary antibody at room temperature for 2 h. ECL chemiluminescent reagent was used to visualize the protein bands, and the gray values of the bands were analyzed using ImageJ software.

### 4.11. Statistical analysis

Statistical analyses adhering to a normal distribution are presented as mean ± standard deviation (SD) and were conducted using SPSS software (version 22.0). Comparisons among multiple groups for quantitative data were performed by one-way analysis of variance (ANOVA) using GraphPad Prism 8.0.2 software. Dunnett's multiple comparison test was used for pairwise comparisons between multiple groups (*p* < 0.05).

## Conflicts of interest

The authors declare that they have no known competing financial interests or personal relationships that could influence the work reported in this study.

## Supplementary Material

RA-015-D5RA05055C-s001

## Data Availability

All data generated or analysed during this study are included in this published article and its supplementary information (SI) file. Supplementary information: the characterization data of all compounds and the copies of representative ^1^H and ^13^C NMR spectra. See DOI: https://doi.org/10.1039/d5ra05055c.
