# Supplementary material for: Novel pyrrolo[2,1-a]isoquinoline aryl ketones attenuate carbon nanotube-induced acute lung injury through NF-κB pathway inhibition
Source: RSC Adv. 2025 Nov 6;15(50):43003–12. doi: 10.1039/d5ra05055c (PMC12590356; doi:10.1039/d5ra05055c)
Supplement: RA-015-D5RA05055C-s001 [file RA-015-D5RA05055C-s001.pdf]

Supplementary Information (SI) for RSC Advances.
This journal is © The Royal Society of Chemistry 2025

Supporting Information

Novel pyrrolo[2,1-*a*]isoquinoline aryl ketones attenuate carbon nanotube-induced acute lung injury through NF-κB pathway inhibition

Guo-Liang Qiu,[1] Jing Zhang,[2] Zongze Yao,[2] Wei Shao,[4] Jiahong Ma,[3] Hua-Li Qin,[3] Wenjian Tang[2,4]

[1] Department of Pharmacy, Anhui No.2 Provincial People's Hospital, Hefei 230041, China

[2] Anhui Province Key Laboratory of Occupational Health, Anhui No.2 Provincial People's Hospital, Hefei 230041, China

[3] School of Chemistry, Chemical Engineering and Life Science, Wuhan University of Technology, Wuhan 430070, China

[4] School of Pharmacy, Anhui Medical University, Hefei 230032, China

Table of contents

1. General information S2

2. General procedure for preparation of **3a–3q**...S2

3. The preparation of **3g** and **3k** as samples for animal experiments..........................S2

4. Characterization data of **3a–3q**..S3

5. Raw data of Western blotting...S11

6. The copies of NMR spectra of **3a–3q**...S22

7. The copies of HPLC spectra of **3a–3q**..S54

1. General Information

All reactions were carried out under an air atmosphere unless otherwise specified. Oil bath was used for the heating reactions. NMR spectra were recorded in $CDCl_3$ on a 500 MHz (for ^{1}H), 471 MHz (for ^{19}F), 126 MHz (for ^{13}C) spectrometer. All chemical shifts are reported in ppm relative to TMS (0 ppm) as an internal standard. The following abbreviations were used to explain the multiplicities: *s* = singlet, *d* = doublet, *t* = triplet, *q* = quartet, *m* = multiplet. The coupling constants were reported in Hertz (Hz). All reagents used in the reactions were all purchased from commercial sources and used without further purification. The product spots on the thin layer chromatography (TLC) were visualized under ultraviolet light (254 nm or 365 nm).

2. General procedure for preparation of **3a–3q**

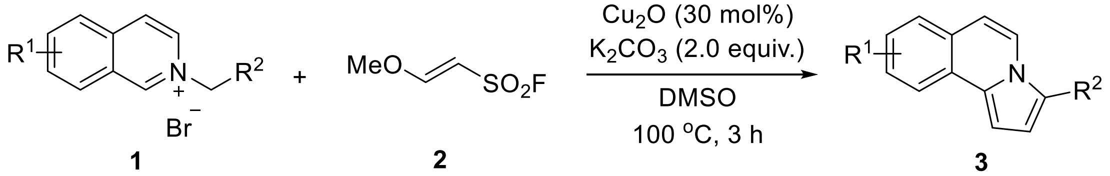

An oven-dried reaction tube equipped with a magnetic stirring bar was charged with isoquinolinium *N*-ylides (**1**, 1.0 mmol), Cu_2O (30 mol%, 43.0 mg), K_2CO_3 (2.0 mmol, 2.0 equiv., 276.0 mg), DMSO (5.0 mL) and 2-methoxyethene-1-sulfonyl fluoride (MESF, 3.0 mmol, 3.0 equiv., 420.0 mg). Then the mixture was stirred at 100 °C for 3 h. After the reaction was completed, the mixture was extracted with ethyl acetate (3 × 20 mL) and the combined organic layers were further washed with brine, and dried over anhydrous sodium sulfate. The solvent was concentrated under reduced pressure and the residue was further purified by flash silica gel chromatography using a mixture of petroleum ether, dichloromethane and ethyl acetate as eluent to afford the title products **3a–3q**.

3. The preparation of **3g** and **3k** as samples for animal experiments

An oven-dried reaction tube equipped with a magnetic stirring bar was charged with isoquinolinium *N*-ylides (**1**, 10.0 mmol), Cu_2O (30 mol%, 429.0 mg), K_2CO_3 (20.0

mmol, 2.0 equiv., 2.76 g), DMSO (45.0 mL) and 2-methoxyethene-1-sulfonyl fluoride (MESF, 50.0 mmol, 30 equiv., 4.20 g). Then the mixture was stirred at 60 °C for 24 h. After the reaction was completed, the mixture was extracted with ethyl acetate (3 × 150 mL) and the combined organic layers were further washed with brine, and dried over anhydrous sodium sulfate. The solvent was concentrated under reduced pressure and the residue was further purified by flash silica gel chromatography using a mixture of petroleum ether and ethyl acetate as eluent to afford products **3g** and **3k**, their yields were 72% and 81%, respectively.

4. Characterization data of **3a–3q**

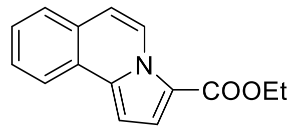

3a

Ethyl pyrrolo[2,1-a]isoquinoline-3-carboxylate (**3a**). White solid, 187 mg, 78% yield. Purified by column chromatography on silica gel using petroleum ether / ethyl acetate = 10:1 (v/v) as eluent, purity, 98.84%. **^{1}H NMR** (500 MHz, $CDCl_3$) δ 9.23 (d, J = 7.5 Hz, 1H), 8.11 (d, J = 8.0 Hz, 1H), 7.66 (d, J = 7.5 Hz, 1H), 7.55-7.48 (m, 3H), 7.00 (d, J = 5.0 Hz, 2H), 4.40 (q, J = 7.0 Hz, 2H), 1.43 (t, J = 7.0 Hz, 3H). **^{13}C NMR** (126 MHz, $CDCl_3$) δ 161.5, 135.4, 127.9, 127.6, 127.3, 126.9, 125.2, 125.0, 123.2, 120.6, 116.7, 112.6, 101.0, 60.0, 14.6.

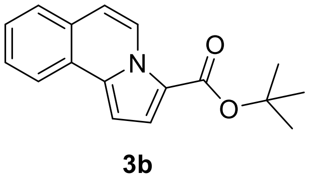

3b

Tert-butyl pyrrolo[2,1-a]isoquinoline-3-carboxylate (**3b**). White solid, 200 mg, 75% yield. Purified by column chromatography on silica gel using petroleum ether / ethyl acetate = 10:1 (v/v) as eluent, purity, 98.02%. **^{1}H NMR** (500 MHz, $CDCl_3$) δ 9.22 (d, J = 7.5 Hz, 1H), 8.11 (d, J = 8.0 Hz, 1H), 7.65 (d, J = 7.5 Hz, 1H), 7.54-7.43 (m, 3H), 6.97 (d, J = 8.0 Hz, 2H), 1.65 (s, 9H). **^{13}C NMR** (126 MHz, $CDCl_3$) δ 161.2, 135.1,

127.8, 127.6, 127.2, 126.9, 125.4, 125.1, 123.1, 120.5, 118.0, 112.4, 100.7, 80.7, 28.7.

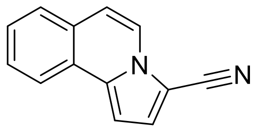

3c

Pyrrolo[2,1-a]isoquinoline-3-carbonitrile (**3c**). White solid, 115 mg, 60% yield. Purified by column chromatography on silica gel using petroleum ether / ethyl acetate = 10:1 (v/v) as eluent, purity, 99.89%. **^{1}H NMR** (500 MHz, $CDCl_3$) δ 8.03 (d, J = 8.0 Hz, 1H), 7.97 (d, J = 7.5 Hz, 1H), 7.62 (d, J = 8.0 Hz, 1H), 7.56-7.47 (m, 2H), 7.25 (d, J = 4.0 Hz, 1H), 6.97 (d, J = 7.0 Hz, 1H), 6.90 (d, J = 4.0 Hz, 1H). **^{13}C NMR** (126 MHz, $CDCl_3$) δ 134.3, 128.5, 127.9, 127.7, 127.4, 125.1, 123.1, 122.6, 121.9, 113.8, 113.7, 101.5, 97.8.

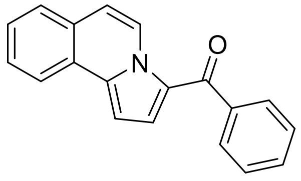

3d

Phenyl(pyrrolo[2,1-a]isoquinolin-3-yl)methanone (**3d**). Yellow solid, 190 mg, 70% yield. Purified by column chromatography on silica gel using petroleum ether / dichloromethane = 1:1 (v/v) as eluent, purity, 99.99%. **^{1}H NMR** (500 MHz, $CDCl_3$) δ 9.62 (d, J = 7.5 Hz, 1H), 8.18 (d, J = 7.5 Hz, 1H), 7.86 (d, J = 7.5 Hz, 2H), 7.73 (d, J = 7.0 Hz, 1H), 7.59-7.49 (m, 5H), 7.32 (d, J = 4.5 Hz, 1H), 7.12 (d, J = 7.5 Hz, 1H), 7.05 (d, J = 4.0 Hz, 1H). **^{13}C NMR** (126 MHz, $CDCl_3$) δ 185.5, 140.7, 137.1, 131.2, 129.2, 129.0, 128.3, 128.1, 127.8, 127.0, 126.0, 125.9, 124.8, 123.7, 113.5, 102.0. Note: In the ^{13}C NMR spectrum of **3k**, theoretically, there should be seventeen peaks. Due to the compact overlaying, it is difficult to specify the overlaying peaks.

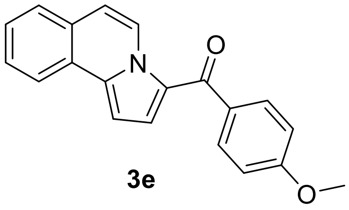

3e

(4-Methoxyphenyl)(pyrrolo[2,1-a]isoquinolin-3-yl)methanone (**3e**). Yellow solid, 151 mg, 50 % yield. Purified by column chromatography on silica gel using petroleum ether / dichloromethane = 1:1 (v/v) as eluent, purity, 98.81%. ^{1}**H NMR** (500 MHz, $CDCl_3$) δ 9.54 (d, J = 8.0 Hz, 1H), 8.17 (d, J = 7.5 Hz, 1H), 7.89-7.86 (m, 2H), 7.72-7.71 (m, 1H), 7.59-7.52 (m, 2H), 7.32 (d, J = 4.5 Hz, 1H), 7.09 (d, J = 7.5 Hz, 1H), 7.05 (d, J = 4.5 Hz, 1H), 7.02-6.99 (m, 2H), 3.90 (s, 3H). ^{13}**C NMR** (126 MHz, $CDCl_3$) δ 184.6, 162.4, 136.7, 133.2, 131.5, 129.0, 128.0, 127.8, 127.0, 126.0, 125.3, 124.9, 123.7, 113.6, 113.3, 101.8, 55.6. Note: In the ^{13}C NMR spectrum of **3m**, theoretically, there should be eighteen peaks. Due to the compact overlaying, it is difficult to specify the overlaying peaks.

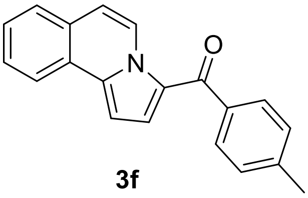

3f

Pyrrolo[2,1-a]isoquinolin-3-yl(p-tolyl)methanone (**3f**). Yellow solid, 257 mg, 90% yield. Purified by column chromatography on silica gel using petroleum ether / dichloromethane = 1:1 (v/v) as eluent, purity, 95.08%. ^{1}**H NMR** (500 MHz, $CDCl_3$) δ 9.59 (d, J = 7.5 Hz, 1H), 8.17 (d, J = 7.5 Hz, 1H), 7.77 (d, J = 8.0 Hz, 2H), 7.73-7.71 (m, 1H), 7.59-7.53 (m, 2H), 7.33-7.30 (m, 3H), 7.11 (d, J = 8.0 Hz, 1H), 7.05 (d, J = 4.0 Hz, 1H), 2.46 (s, 3H). ^{13}**C NMR** (126 MHz, $CDCl_3$) δ 185.5, 141.8, 138.0, 136.9, 129.4, 129.0, 129.0, 128.1, 127.8, 127.0, 126.0, 125.8, 125.0, 124.9, 123.7, 113.4, 101.9, 21.7.

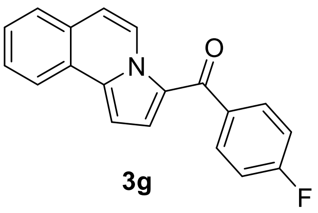

3g

(4-Fluorophenyl)(pyrrolo[2,1-a]isoquinolin-3-yl)methanone (**3g**). Yellow solid, 119 mg, 41% yield. Purified by column chromatography on silica gel using petroleum ether / dichloromethane = 1:1 (v/v) as eluent, purity, 99.99%. **^{1}H NMR** (500 MHz, $CDCl_3$) δ 9.57 (d, J = 7.0 Hz, 1H), 8.19 (d, J = 7.5 Hz, 1H), 7.89-7.87 (m, 2H), 7.75-7.73 (m, 1H), 7.61-7.55 (m, 2H), 7.29 (d, J = 4.5 Hz, 1H), 7.20-7.17 (m, 2H), 7.14 (d, J = 7.5 Hz, 1H), 7.07 (d, J = 4.5 Hz, 1H). **^{13}C NMR** (126 MHz, $CDCl_3$) δ 183.9, 164.7 (d, J = 252.0 Hz), 137.1, 136.8 (d, J = 3.7 Hz), 131.5 (d, J = 8.2 Hz), 129.0, 128.1, 127.8, 127.0, 125.8, 125.7, 124.7, 124.5, 123.7, 115.3 (d, J = 21.8 Hz), 113.5, 102.0.

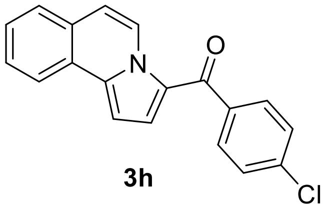

3h

(4-Chlorophenyl)(pyrrolo[2,1-a]isoquinolin-3-yl)methanone (**3h**). Yellow solid, 122 mg, 40% yield. Purified by column chromatography on silica gel using petroleum ether / dichloromethane = 1:1 (v/v) as eluent, purity, 98.90%. **^{1}H NMR** (500 MHz, $CDCl_3$) δ 9.58 (d, J = 7.5 Hz, 1H), 8.19 (d, J = 7.5 Hz, 1H), 7.80 (d, J = 8.5 Hz, 2H), 7.75-7.73 (m, 1H), 7.61-7.56 (m, 2H), 7.48 (d, J = 8.5 Hz, 2H), 7.28 (d, J = 4.5 Hz, 1H), 7.14 (d, J = 7.5 Hz, 1H), 7.07 (d, J = 4.5 Hz, 1H). **^{13}C NMR** (126 MHz, $CDCl_3$) δ 184.0, 165.9, 139.1, 137.5, 137.4, 130.6, 129.1, 128.6, 128.3, 128.0, 127.1, 125.9, 124.7, 124.5, 123.8, 113.7, 102.3.

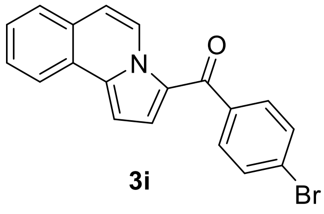

3i

(4-Bromophenyl)(pyrrolo[2,1-a]isoquinolin-3-yl)methanone (**3i**). Yellow solid, 122 mg, 48% yield. Purified by column chromatography on silica gel using petroleum ether / dichloromethane = 1:1 (v/v) as eluent, purity, 99.99%. **^{1}H NMR** (500 MHz, $CDCl_3$) δ 9.58 (d, J = 7.5 Hz, 1H), 8.20-8.18 (m, 1H), 7.75-7.71 (m, 3H), 7.66-7.63 (m, 2H), 7.61-7.55 (m, 2H), 7.28 (d, J =4.5 Hz, 1H), 7.14 (d, J = 7.5 Hz, 1H), 7.06 (d, J = 4.5 Hz, 1H). **^{13}C NMR** (126 MHz, $CDCl_3$) δ 184.1, 139.5, 137.4, 131.6, 130.8, 129.1, 128.3, 128.0, 127.1, 125.9, 125.9, 124.7, 124.5, 123.8, 113.7, 102.3. Note: In the ^{13}C NMR spectrum of **3q**, theoretically, there should be seventeen peaks. Due to the compact overlaying, it is difficult to specify the overlaying peaks.

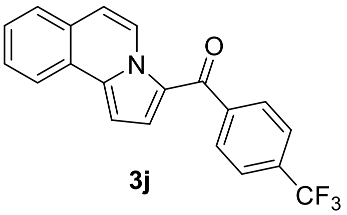

3j

Pyrrolo[2,1-a]isoquinolin-3-yl(4-(trifluoromethyl)phenyl)methanone (**3j**). Yellow solid, 110 mg, 32% yield. M.p. 236-238 °C. Purified by column chromatography on silica gel using petroleum ether / dichloromethane = 1:1 (v/v) as eluent, purity, 98.19%. **^{1}H NMR** (500 MHz, $CDCl_3$) δ 9.64 (d, J = 7.5 Hz, 1H), 8.23-8.21 (m, 1H), 7.96 (d, J = 8.0 Hz, 2H), 7.80-7.77 (m, 3H), 7.65-7.59 (m, 2H), 7.29 (d, J = 4.0 Hz, 1H), 7.20(d, J = 7.5 Hz, 1H), 7.10 (d, J = 5.0 Hz, 1H). **^{13}C NMR** (126 MHz, $CDCl_3$) δ 183.9, 144.0, 137.8, 132.9, 132.6, 129.4, 129.3, 128.5, 128.1, 127.2, 126.4, 125.9, 125.4 (q, J = 3.7 Hz), 125.1, 124.7, 124.4, 123.9, 122.9, 114.0, 102.6. **HRMS-ESI** (m/z) calcd. for $[C_{20}H_{13}F_3NO]^+$ ($[M+H]^+$): 340.0944, found: 340.0951.

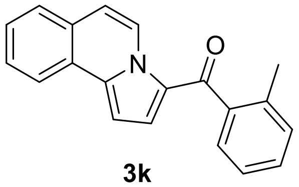

3k

Pyrrolo[2,1-a]isoquinolin-3-yl(o-tolyl)methanone (**3k**). Yellow solid, 177 mg, 62% yield. M.p. 87-89 °C. Purified by column chromatography on silica gel using petroleum

ether / dichloromethane = 1:1 (v/v) as eluent, purity, 99.85%. **^{1}H NMR** (500 MHz, $CDCl_3$) δ 9.73 (d, J = 7.5 Hz, 1H), 8.18 (d, J = 8.0 Hz, 1H), 7.75 (d, J = 7.0 Hz, 1H), 7.60-7.55 (m, 2H), 7.45 (d, J = 7.5 Hz, 1H), 7.39 (t, J = 7.5 Hz, 1H), 7.31-7.27(m, 2H), 7.15 (d, J = 7.5 Hz, 1H), 7.05 (d, J = 4.5 Hz, 1H), 7.01 (d, J = 4.5 Hz, 1H), 2.43 (s, 3H). **^{13}C NMR** (126 MHz, $CDCl_3$) δ 187.5, 140.7, 137.3, 136.2, 130.8, 129.6, 129.2, 128.3, 128.2, 127.9, 127.1, 126.5, 126.1, 125.6, 125.2, 124.8, 123.8, 113.7, 102.2, 19.8. **HRMS-ESI** (m/z) calcd. for $[C_{20}H_{16}NO]^+$ ($[M+H]^+$): 286.1227, found: 286.1234.

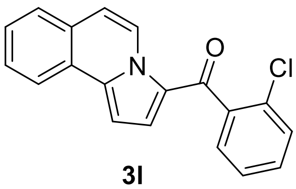

3l

(2-Chlorophenyl)(pyrrolo[2,1-a]isoquinolin-3-yl)methanone (**3l**). Yellow solid, 116 mg, 38% yield. Purified by column chromatography on silica gel using petroleum ether / dichloromethane = 1:1 (v/v) as eluent, purity, 99.83%. **^{1}H NMR** (500 MHz, $CDCl_3$) δ 9.69 (d, J = 7.5 Hz, 1H), 8.18-8.16 (m, 1H), 7.75-7.74 (m, 1H), 7.60-7.56 (m, 2H), 7.50-7.48 (m, 2H), 7.43-7.40 (m, 1H), 7.38-7.35 (m, 1H), 7.17 (d, J = 7.5 Hz, 1H), 7.03-7.00 (m, 2H). **^{13}C NMR** (126 MHz, $CDCl_3$) δ 183.3, 140.0, 137.8, 131.5, 130.6, 130.1, 129.3, 129.2, 128.4, 128.0, 127.1, 126.6, 126.5, 126.0, 124.7, 124.6, 123.9, 113.9, 102.7.

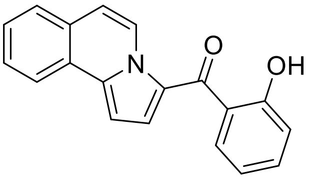

3m

(2-Hydroxyphenyl)(pyrrolo[2,1-a]isoquinolin-3-yl)methanone (**3m**). Yellow solid, 161 mg, 56% yield. M.p. 142-144 °C. Purified by column chromatography on silica gel using petroleum ether / ethyl acetate = 10:1 (v/v) as eluent, purity, 98.51%. **^{1}H NMR** (500 MHz, $CDCl_3$) δ 11.57 (s, 1H), 9.31 (d, J = 7.5 Hz, 1H), 8.18 (d, J = 8.0 Hz, 1H), 7.90 (d, J = 8.0 Hz, 1H), 7.73 (d, J = 7.0 Hz, 1H), 7.61-7.55 (m, 2H), 7.49-7.44 (m, 2H), 7.11-7.06 (m, 3H), 6.96 (t, J = 7.5 Hz, 1H). **^{13}C NMR** (126 MHz, $CDCl_3$) δ 187.1, 162.0,

137.6, 134.6, 132.1, 129.1, 128.4, 128.0, 127.1, 126.1, 125.8, 124.8, 124.2, 123.8, 121.5, 118.8, 118.2, 113.6, 102.6. **HRMS-ESI** (m/z) calcd. for $[C_{19}H_{14}NO_2]^+$ ($[M+H]^+$): 288.1020, found: 288.1015.

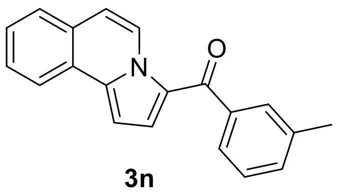

3n

Pyrrolo[2,1-a]isoquinolin-3-yl(m-tolyl)methanone (**3n**). Yellow solid, 185 mg, 65% yield. M.p. 123-125 °C. Purified by column chromatography on silica gel using petroleum ether / dichloromethane = 1:1 (v/v) as eluent, purity, 99.84%. **^{1}H NMR** (500 MHz, $CDCl_3$) δ 9.61 (d, J = 7.0 Hz, 1H), 8.19-8.18 (m, 1H), 7.74-7.72 (m, 1H), 7.66-7.63 (m, 2H), 7.60-7.54 (m, 2H), 7.40-7.37 (m, 2H), 7.32(d, J = 4.0 Hz, 1H), 7.12 (d, J = 7.5 Hz, 1H), 7.05 (d, J = 4.5 Hz, 1H), 2.46 (s, 3H). **^{13}C NMR** (126 MHz, $CDCl_3$) δ 185.8, 140.8, 138.1, 137.0, 132.0, 129.8, 129.1, 128.1, 127.9, 127.1, 126.5, 126.0, 124.9, 124.9, 123.8, 113.5, 102.0, 21.6. Note: In the ^{13}C NMR spectrum of **3v**, theoretically, there should be twenty peaks. Due to the compact overlaying, it is difficult to specify the overlaying peaks. **HRMS-ESI** (m/z) calcd. for $[C_{20}H_{16}NO]^+$ ($[M+H]^+$): 286.1227, found: 286.1225.

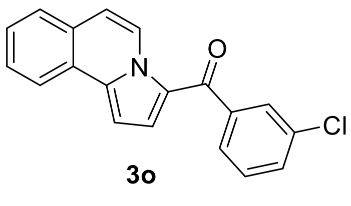

3o

(3-Chlorophenyl)(pyrrolo[2,1-a]isoquinolin-3-yl)methanone (**3o**). Yellow solid, 128 mg, 42% yield. Purified by column chromatography on silica gel using petroleum ether / dichloromethane = 1:1 (v/v) as eluent, purity, 99.38%. **^{1}H NMR** (500 MHz, $CDCl_3$) δ 9.59 (d, J = 7.5 Hz, 1H), 8.21-8.19 (m, 1H), 7.82 (t, J = 2.0 Hz, 1H), 7.76-7.71 (m, 2H), 7.62-7.56 (m, 2H), 7.54-7.52 (m, 1H), 7.44 (t, J = 7.5 Hz, 1H), 7.30 (d, J = 5.0 Hz, 1H), 7.16 (d, J = 7.5 Hz, 1H), 7.07 (d, J = 4.5 Hz, 1H). **^{13}C NMR** (126 MHz, $CDCl_3$) δ 183.7, 142.4, 137.6, 134.5, 131.2, 129.6, 129.2, 129.2, 128.4, 128.0, 127.3, 127.1, 126.2, 125.9,

124.7, 124.4, 123.9, 113.8, 102.4.

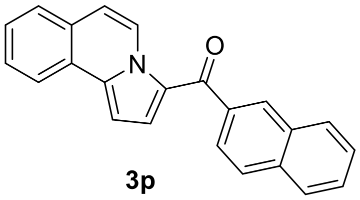

3p

Naphthalen-2-yl(pyrrolo[2,1-a]isoquinolin-3-yl)methanone (**3p**). Yellow solid, 167 mg, 52% yield. Purified by column chromatography on silica gel using petroleum ether / dichloromethane = 1:1 (v/v) as eluent, purity, 98.65%. **^{1}H NMR** (500 MHz, $CDCl_3$) δ 9.65 (d, J = 7.5 Hz, 1H), 8.36 (s, 1H), 8.22-8.20 (m, 1H), 7.98-7.93 (m, 4H), 7.76-7.74 (m, 1H), 7.62-7.56 (m, 4H), 7.39 (d, J = 5.0 Hz, 1H), 7.16 (d, J = 7.5 Hz, 1H), 7.09 (d, J = 4.5 Hz, 1H). **^{13}C NMR** (126 MHz, $CDCl_3$) δ 185.5, 138.0, 137.2, 134.9, 132.6, 130.0, 129.3, 129.1, 128.2, 128.2, 128.0, 127.9, 127.8, 127.1, 126.8, 126.2, 126.0, 125.9, 125.1, 124.9, 123.8, 113.6, 102.2.

Raw data of Western blotting:

P-IKB alpha and ACTIN

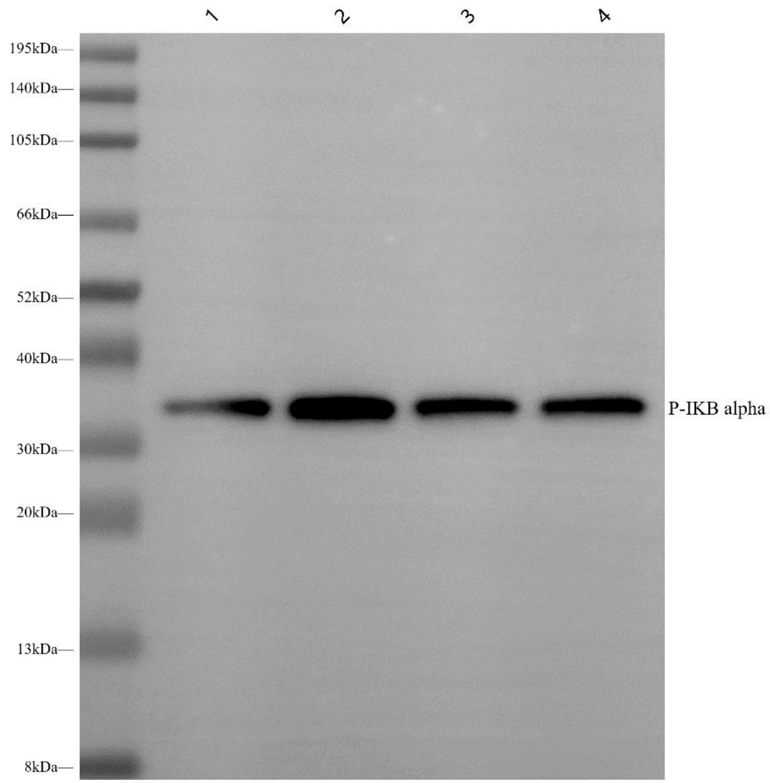

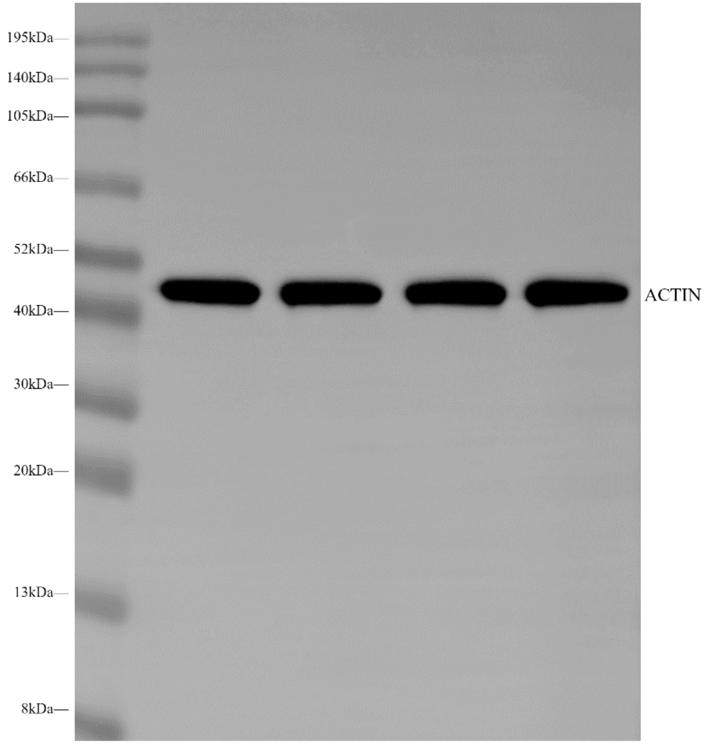

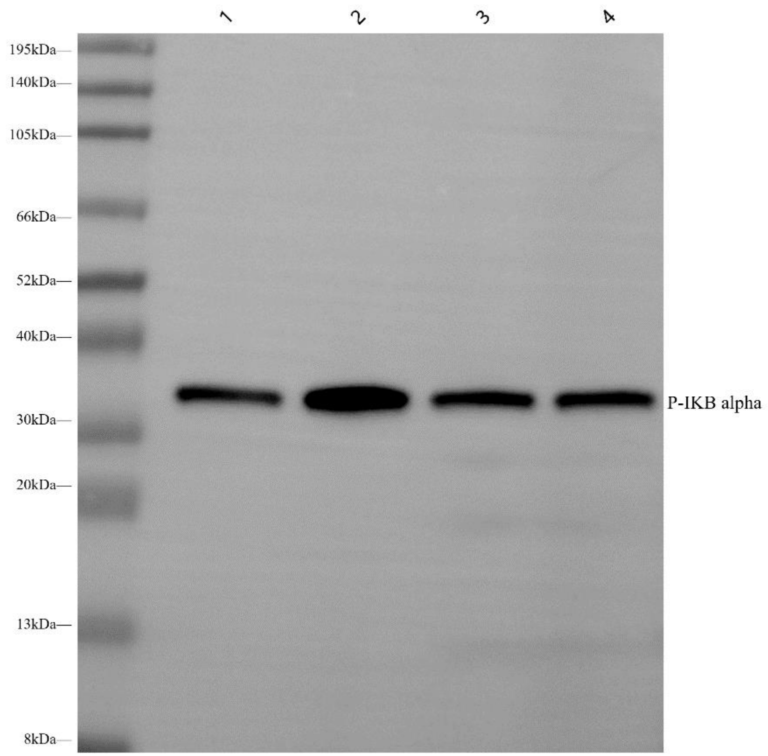
1
2
3
4
195kDa
140kDa
105kDa
66kDa
52kDa
40kDa
30kDa
20kDa
13kDa
8kDa
P-IKB alpha

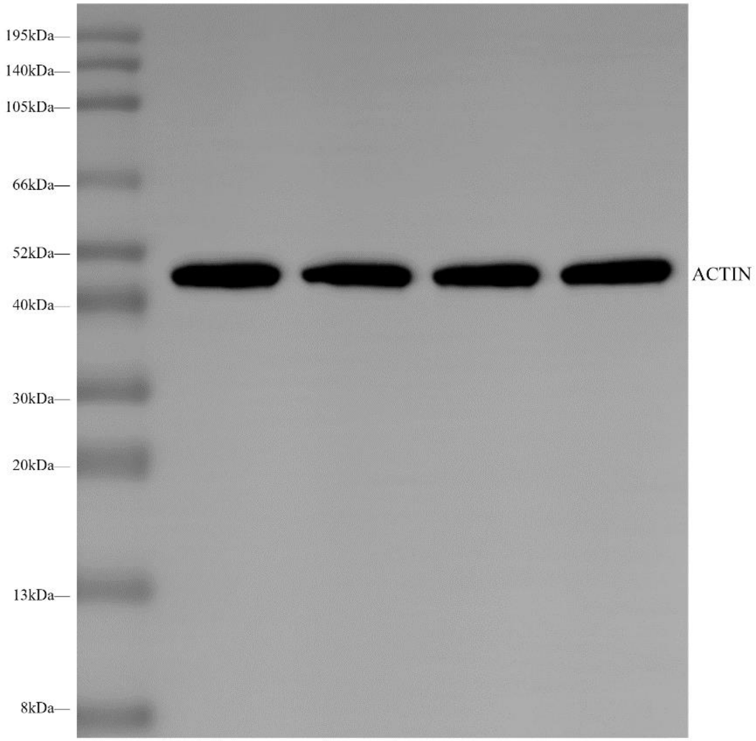
195kDa
140kDa
105kDa
66kDa
52kDa
40kDa
30kDa
20kDa
13kDa
8kDa
ACTIN

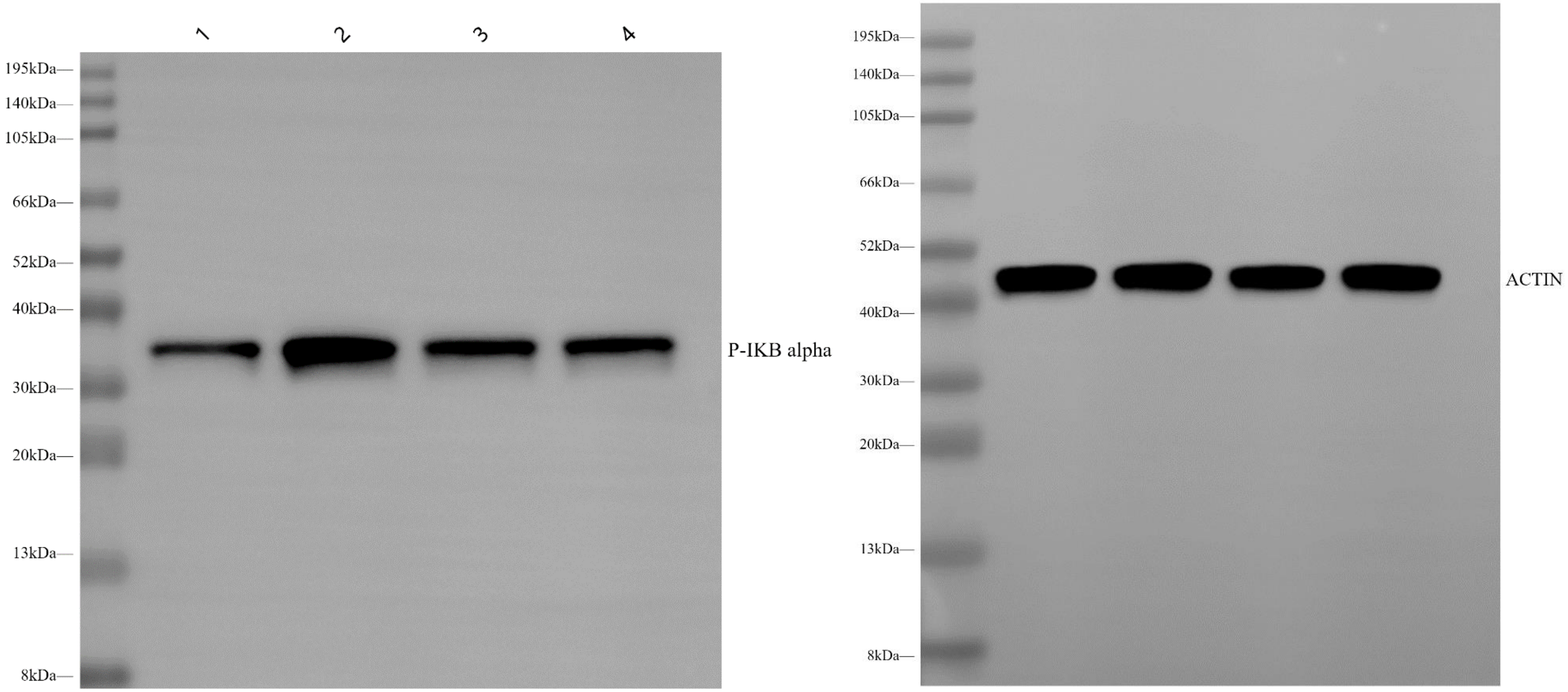
1
2
3
4
195kDa
140kDa
105kDa
66kDa
52kDa
40kDa
30kDa
20kDa
13kDa
8kDa
P-IKB alpha
195kDa
140kDa
105kDa
66kDa
52kDa
40kDa
30kDa
20kDa
13kDa
8kDa
ACTIN

Raw data: P-P65, P65, IKB alpha and ACTIN

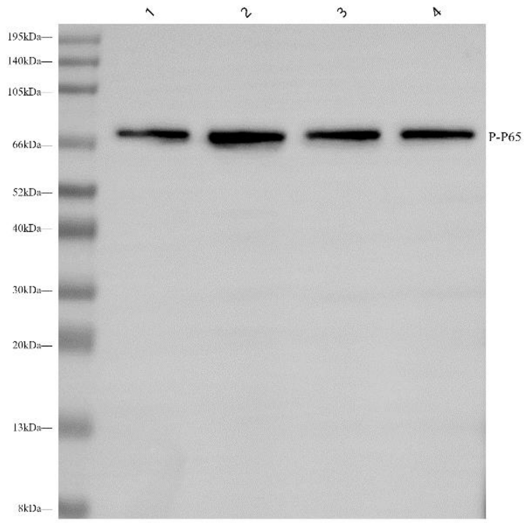

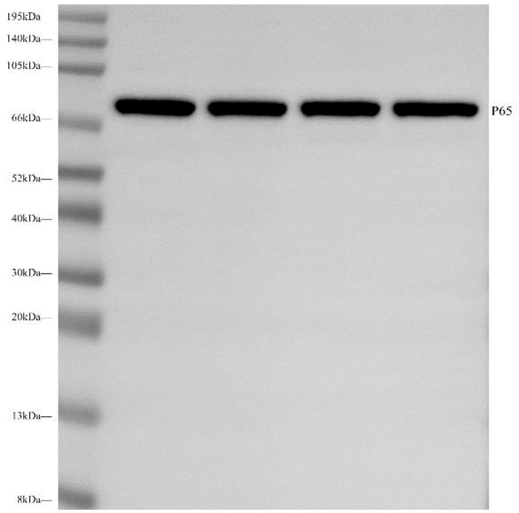

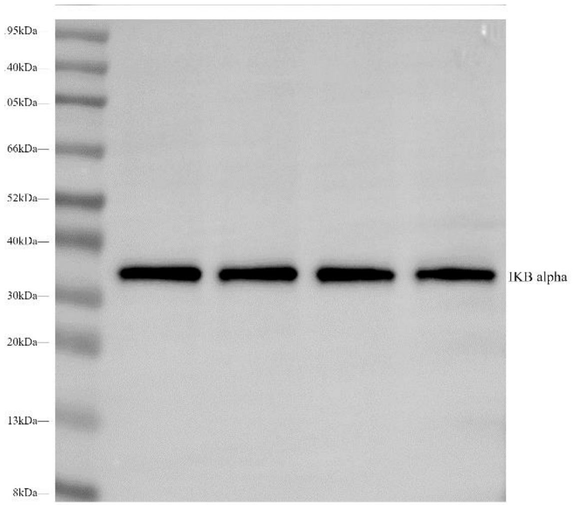

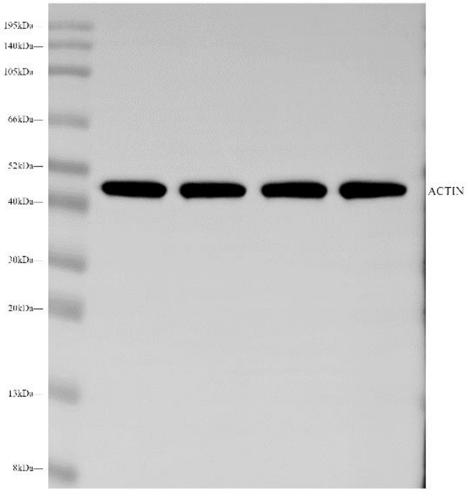

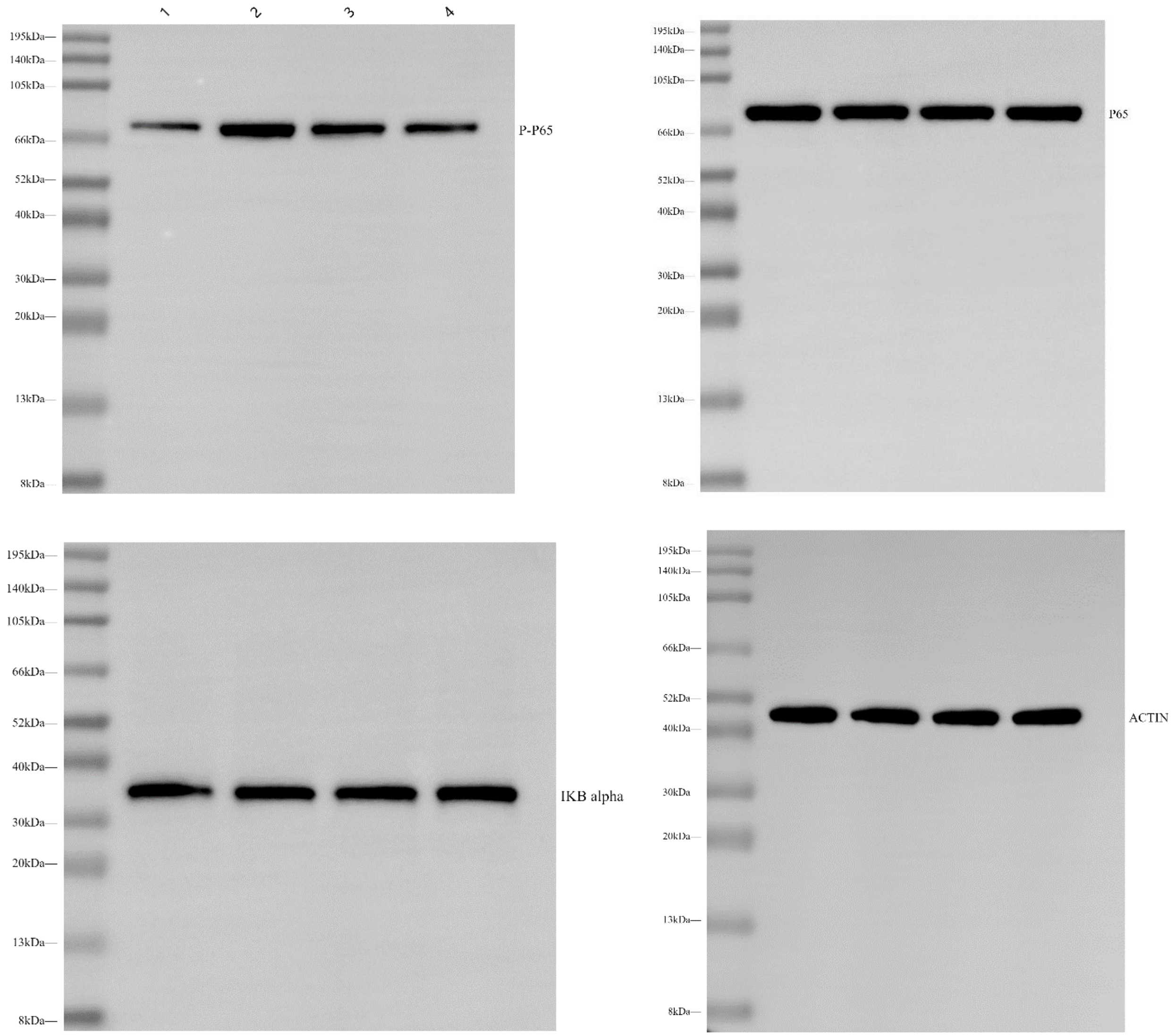
1
2
3
4
195kDa
140kDa
105kDa
66kDa
52kDa
40kDa
30kDa
20kDa
13kDa
8kDa
P-P65
P65
IKB alpha
105Da
ACTIN

S15

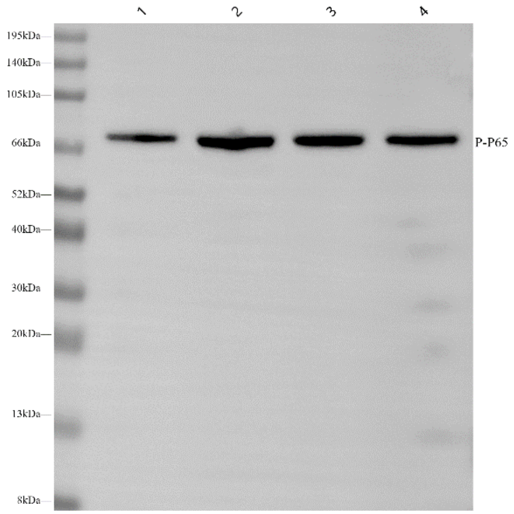
1
2
3
4
195kDa
140kDa
105kDa
66kDa
52kDa
40kDa
30kDa
20kDa
13kDa
8kDa
P-P65

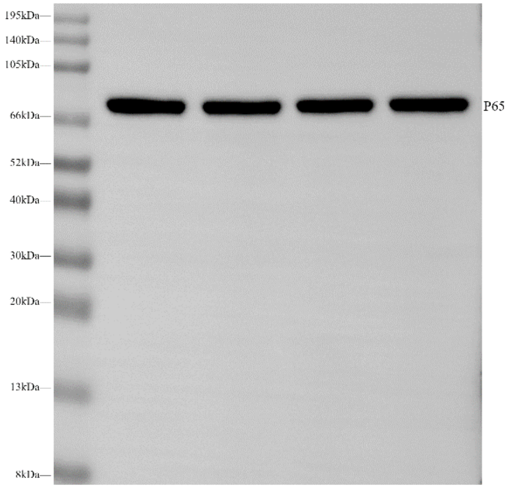
195kDa
140kDa
105kDa
66kDa
52kDa
40kDa
30kDa
20kDa
13kDa
8kDa
P65

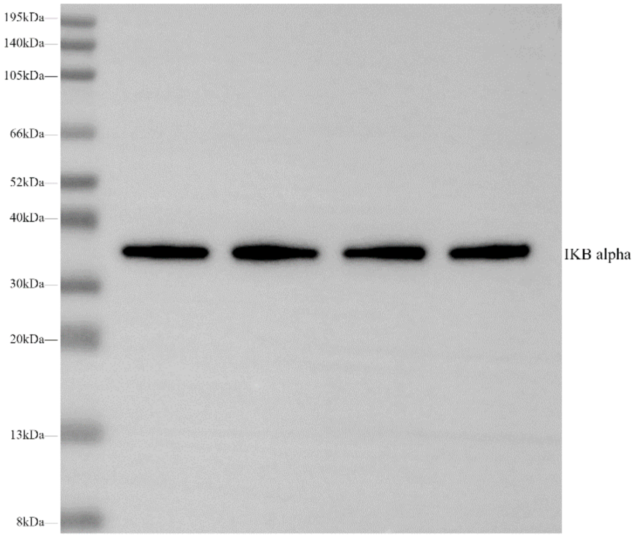
195kDa
140kDa
105kDa
66kDa
52kDa
40kDa
30kDa
20kDa
13kDa
8kDa
IKB alpha

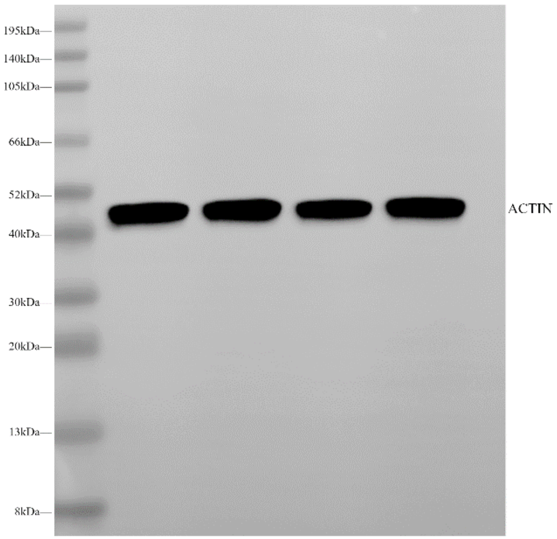
195kDa
140kDa
105kDa
66kDa
52kDa
40kDa
30kDa
20kDa
13kDa
8kDa
ACTIN

S16

Raw data: P-IKB alpha (n = 3)

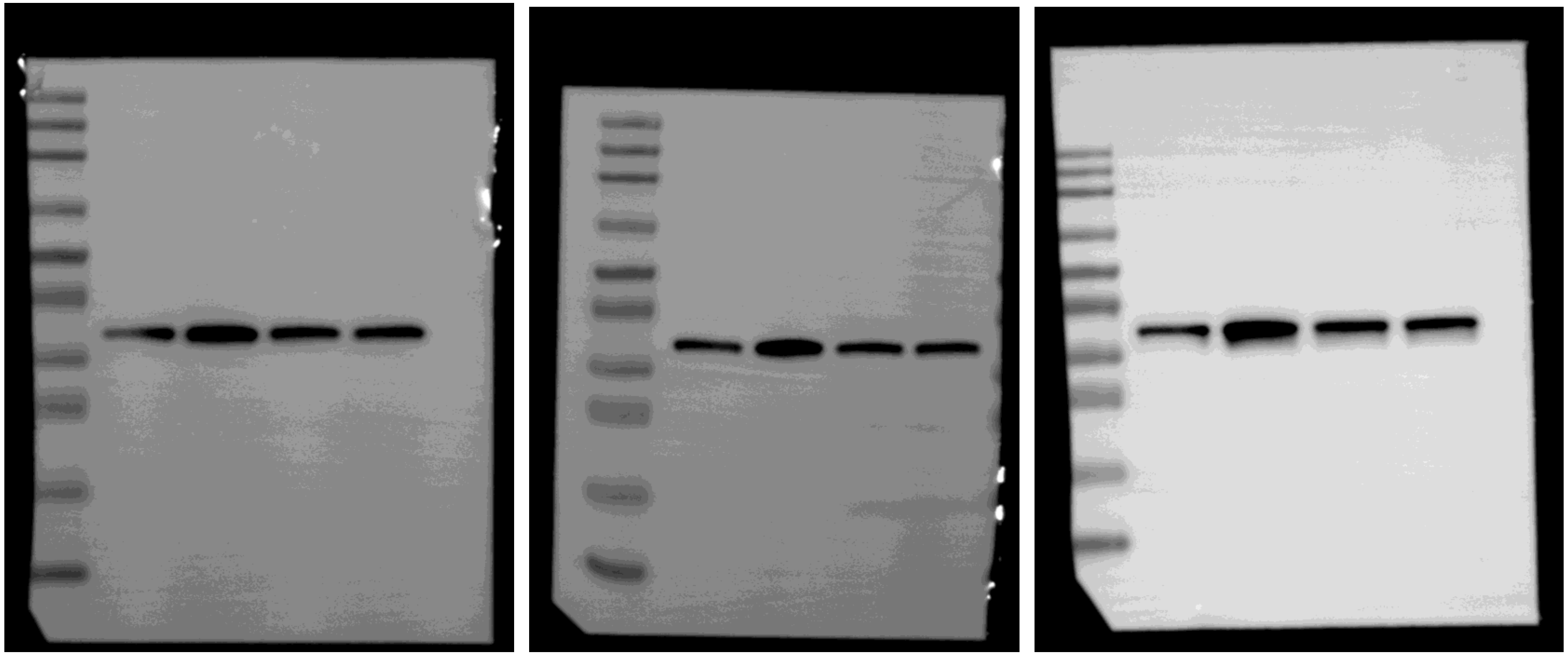

Raw data: IKB alpha (n = 3)

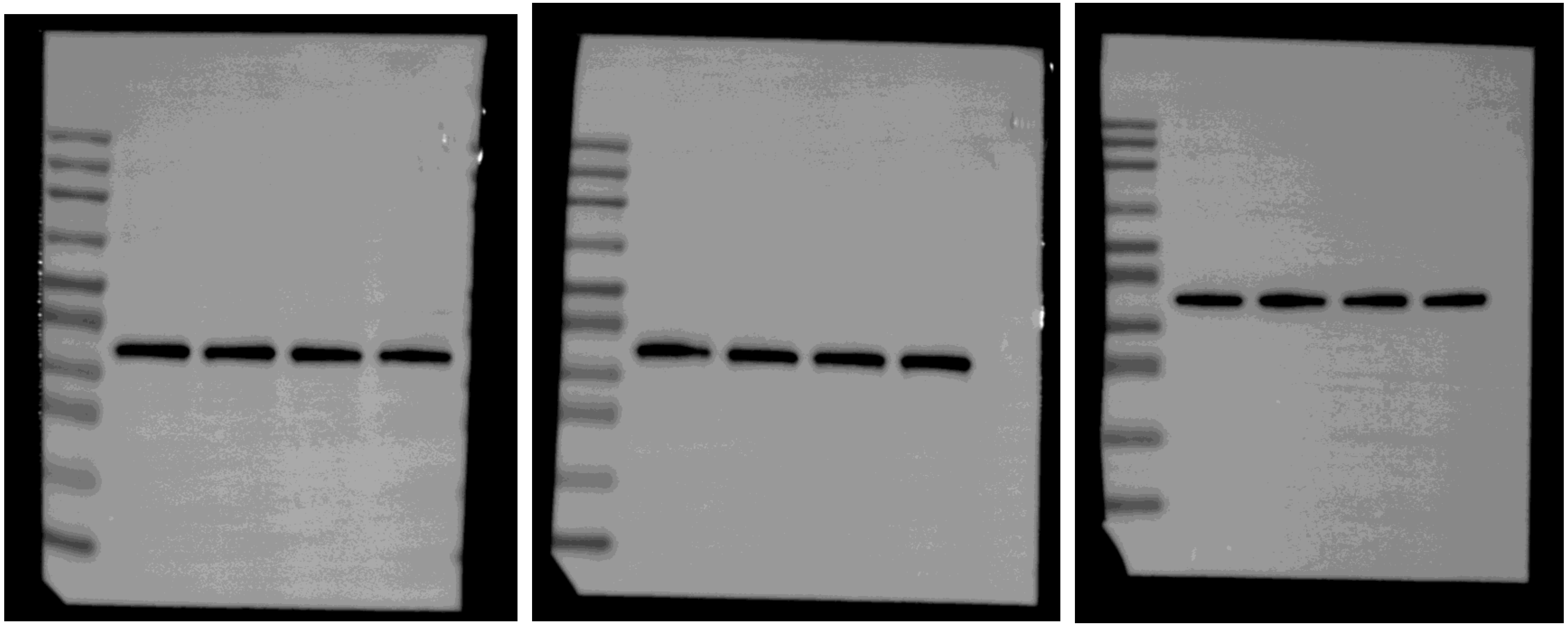

Raw data: P65 (n = 3)

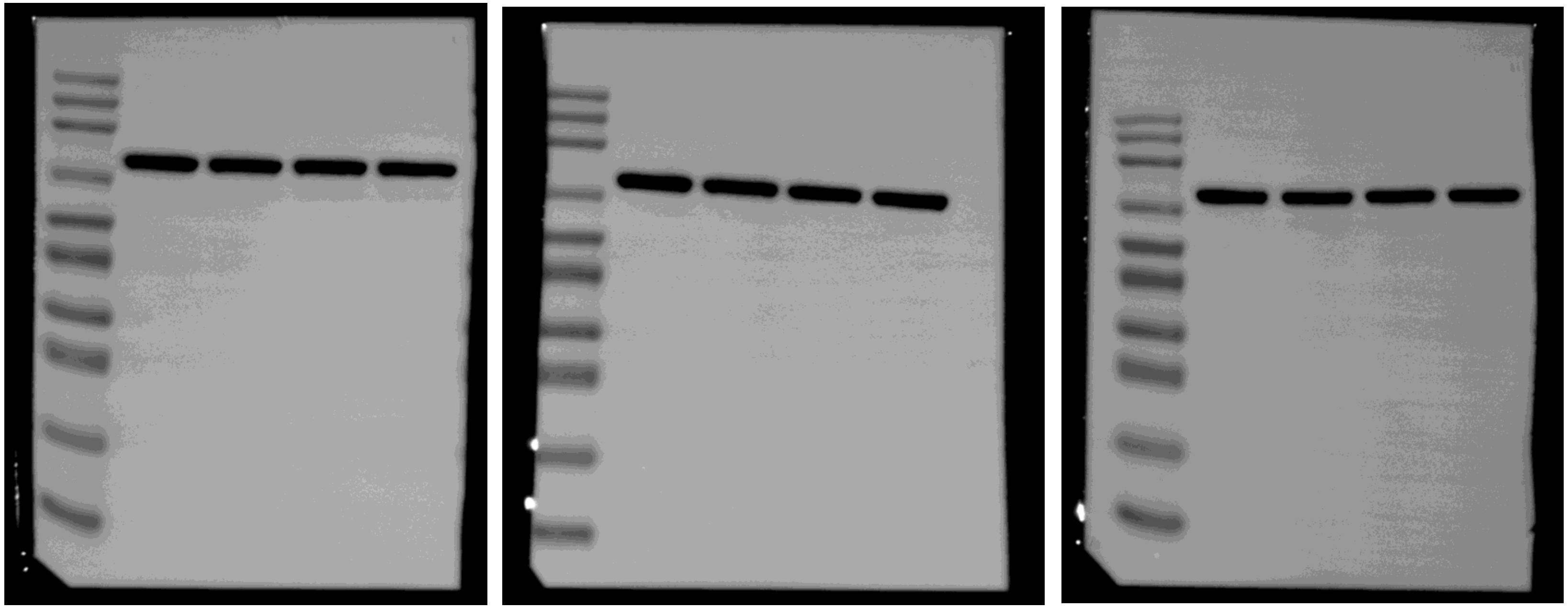

Raw data: P-P65 (n = 3)

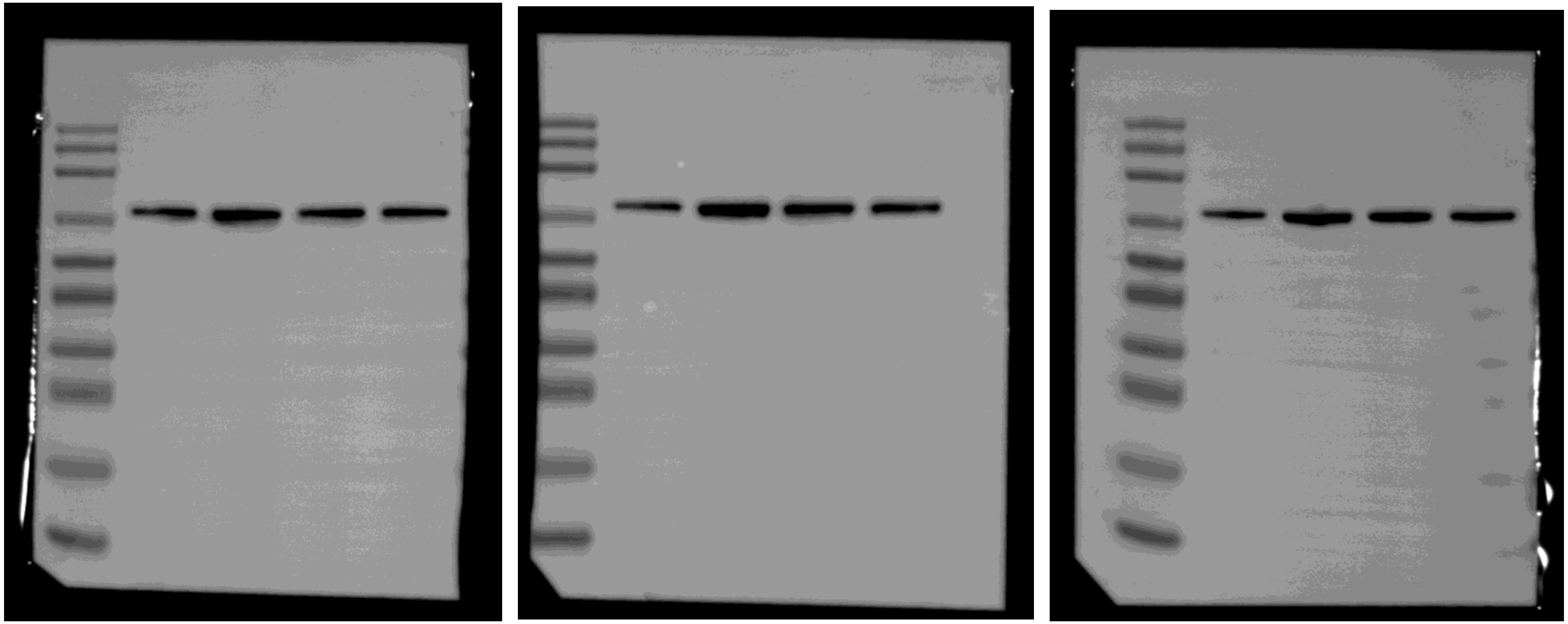

Raw data: ACTIN (n = 3)

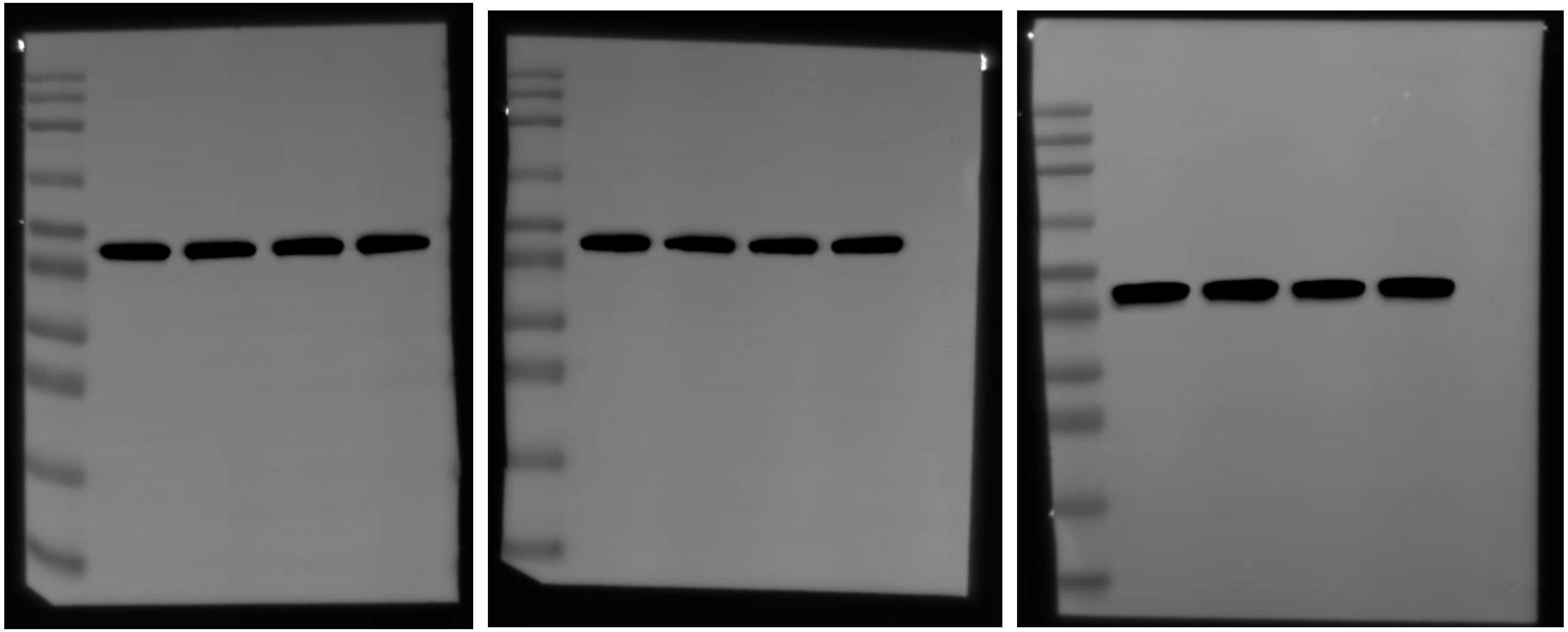

7. NMR and HPLC spectra

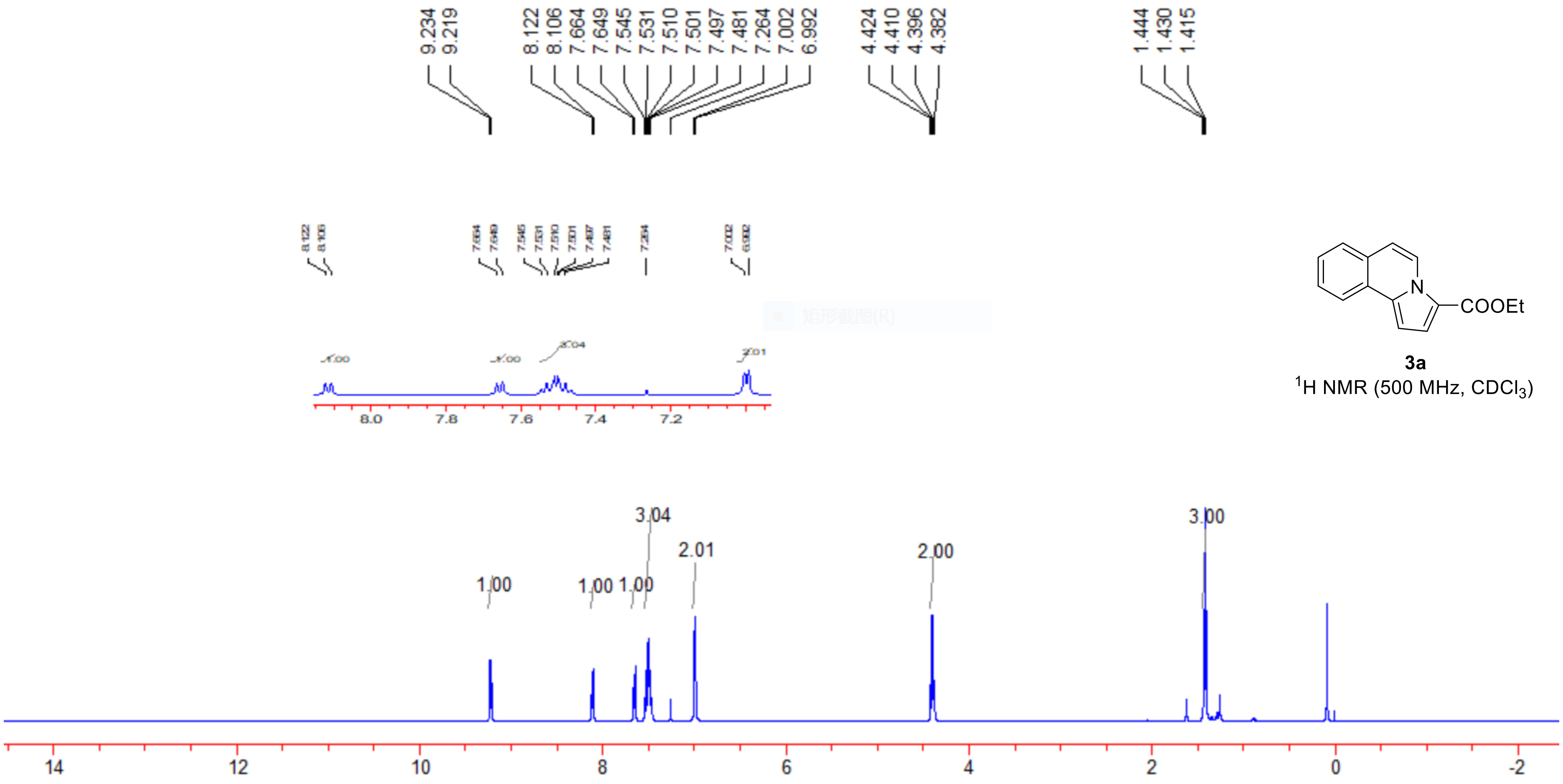

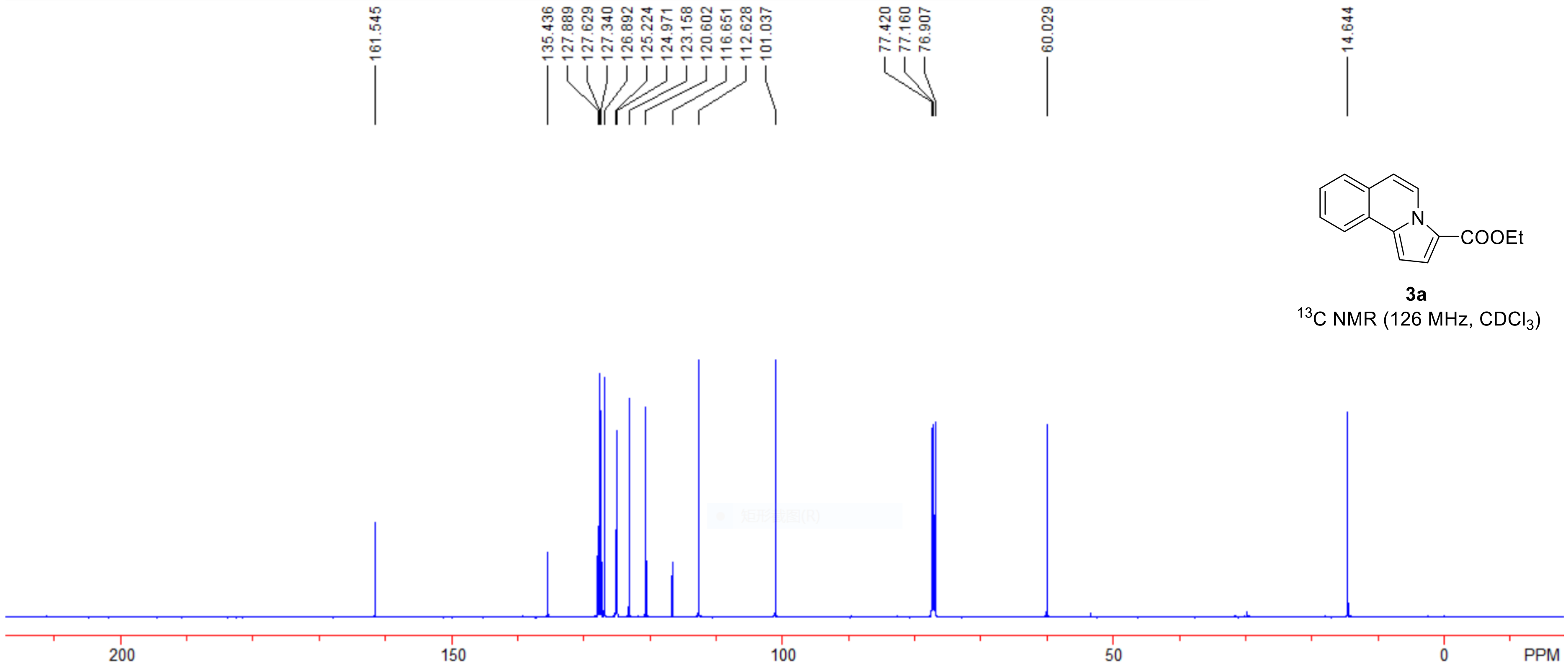

3a

^{13}C NMR (126 MHz, $CDCl_3$)

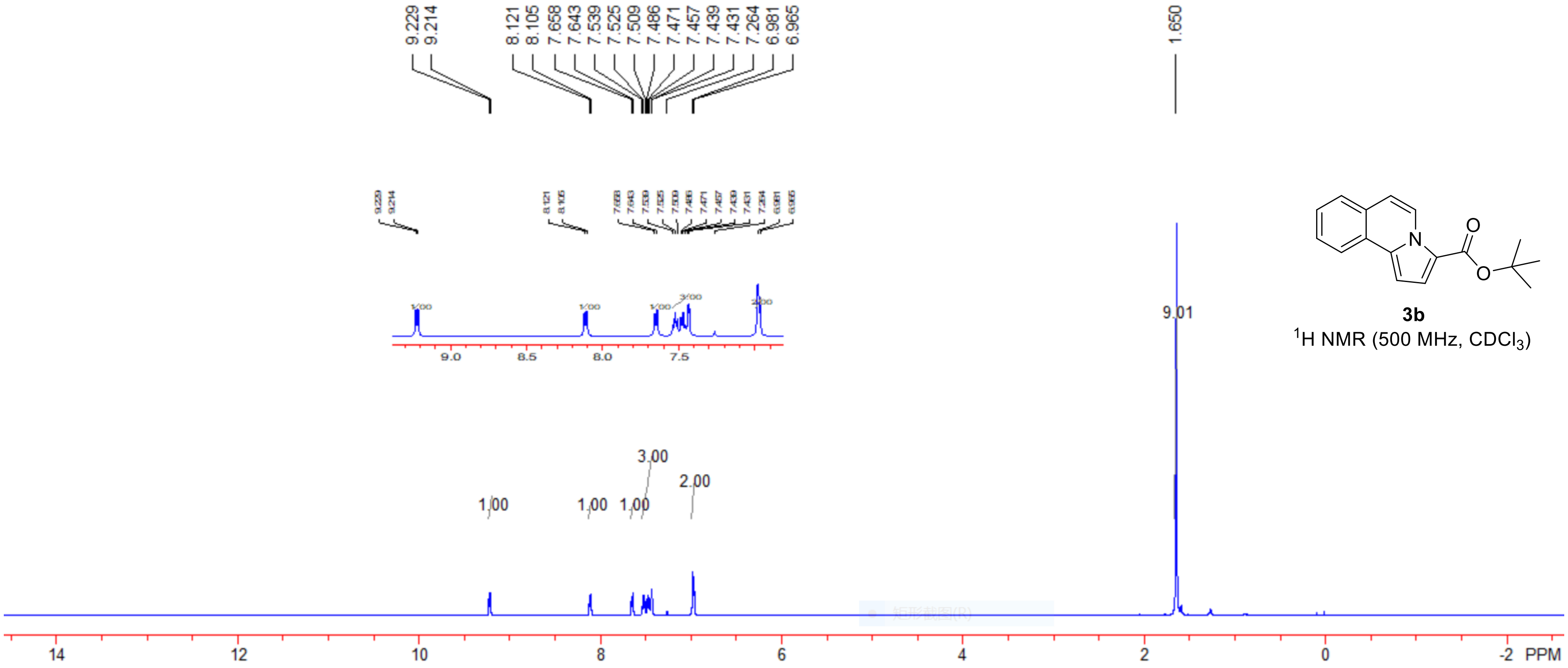

3b

^{1}H NMR (500 MHz, $CDCl_3$)

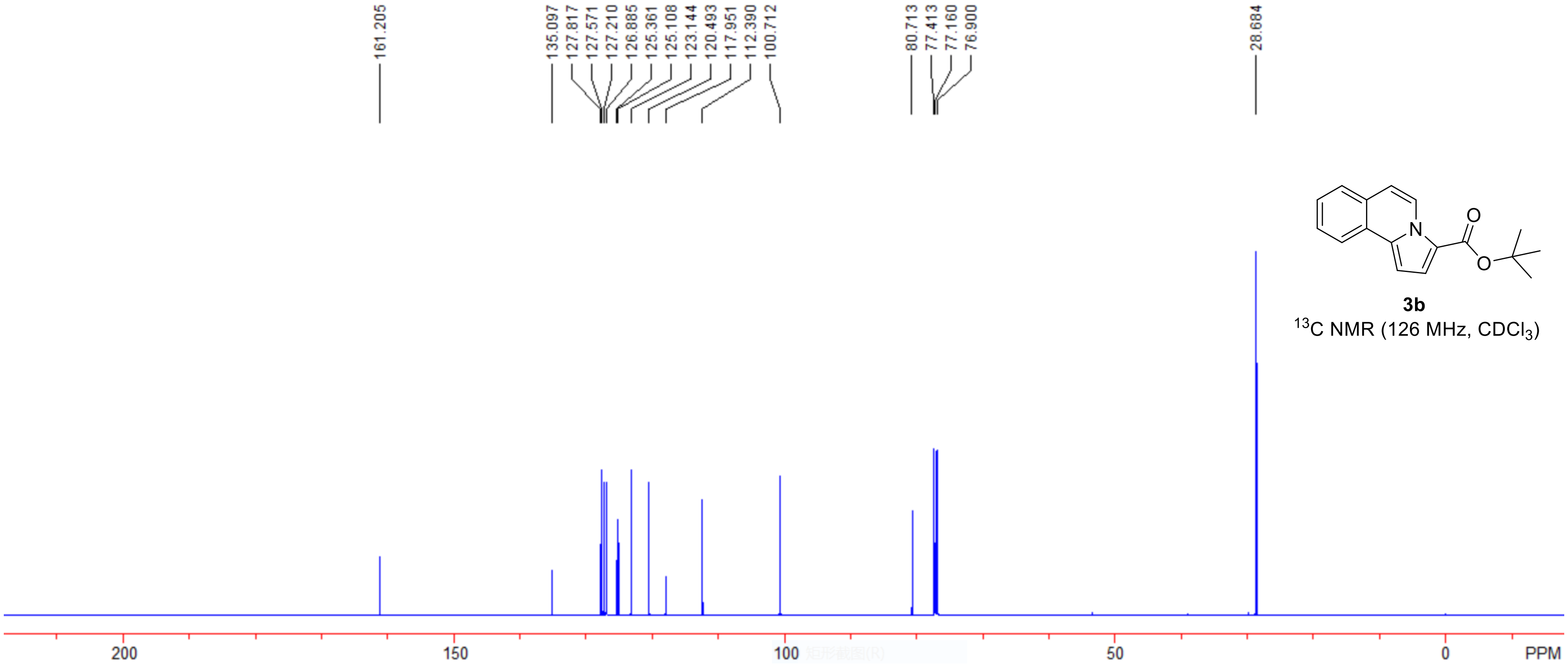

3b

^{13}C NMR (126 MHz, $CDCl_3$)

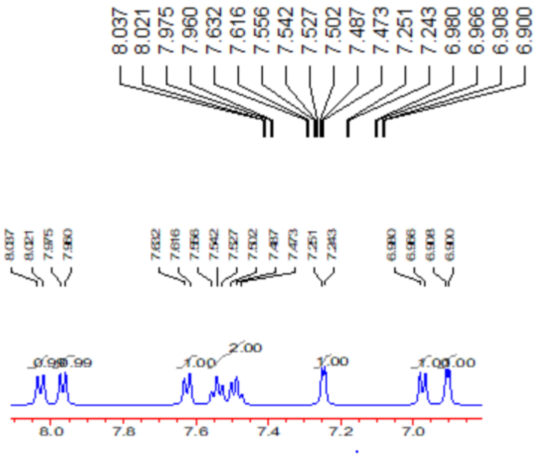

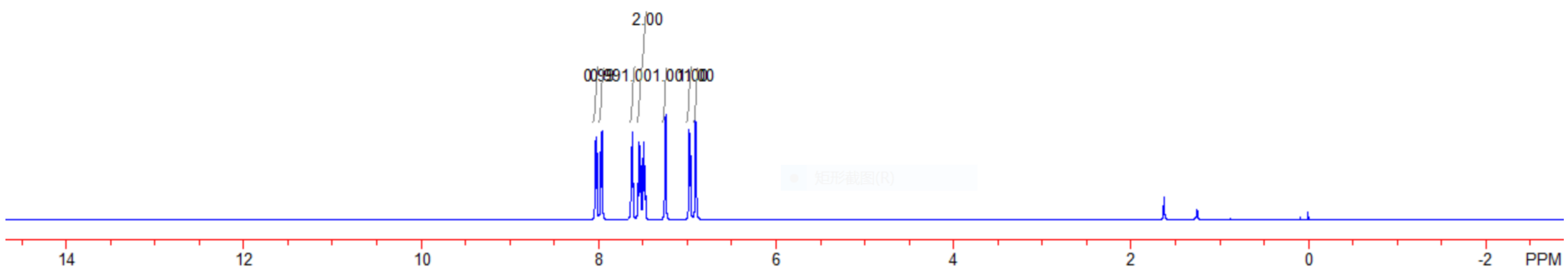

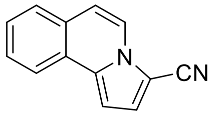

3c
^{1}H NMR (500 MHz, $CDCl_3$)

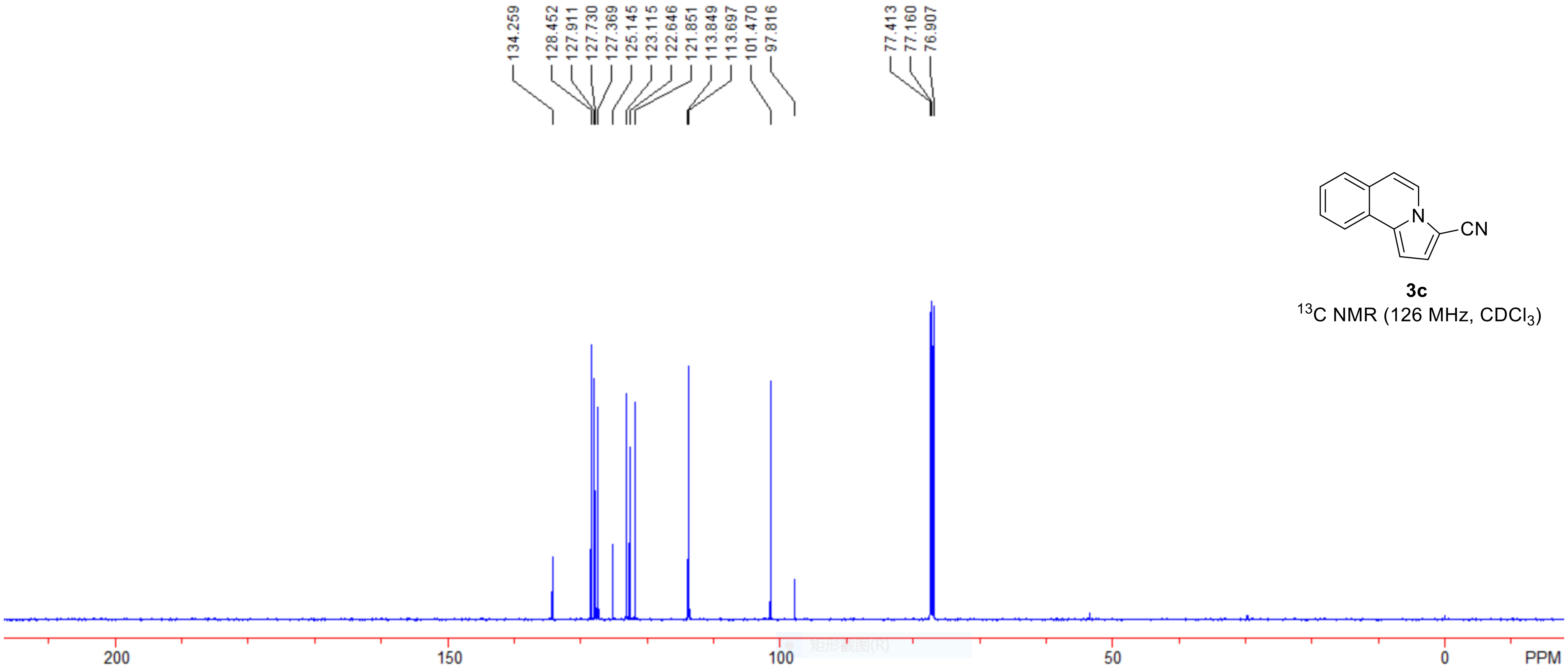

3c

^{13}C NMR (126 MHz, $CDCl_3$)

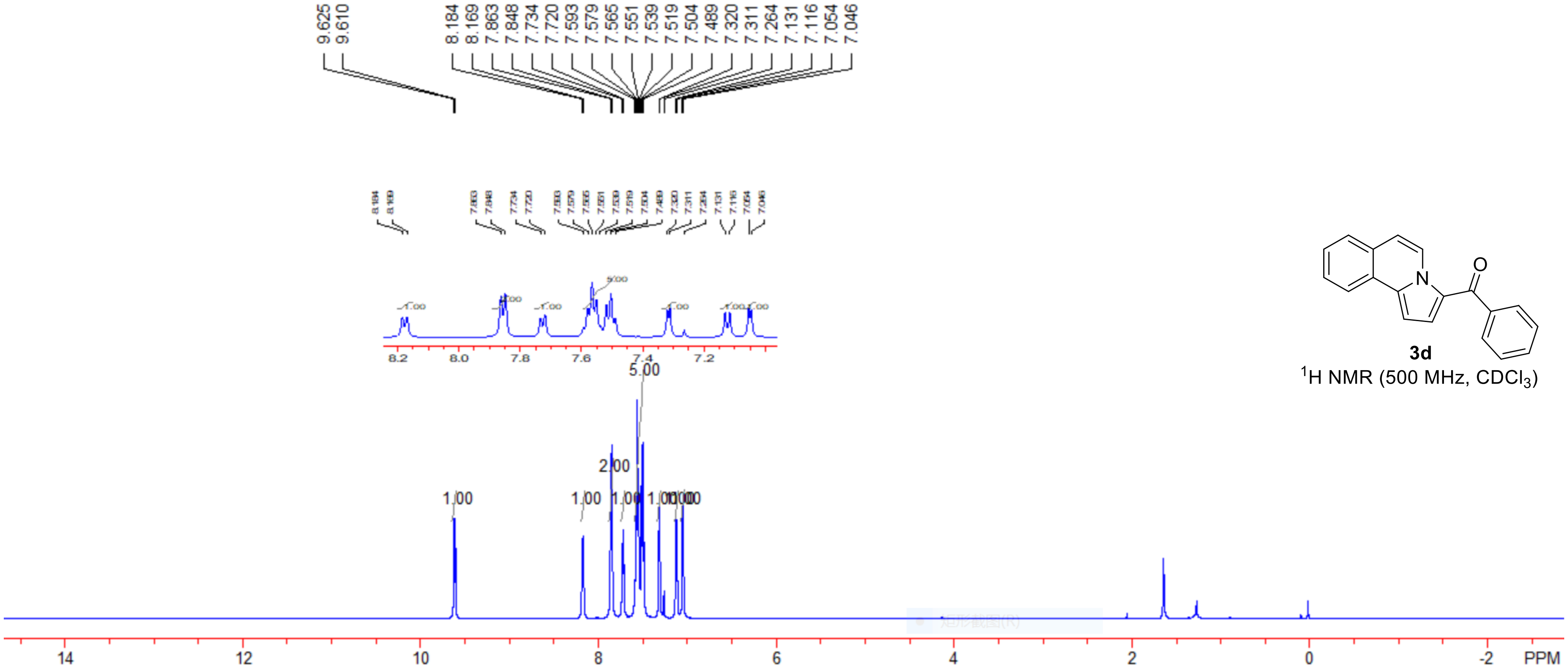

3d

^{1}H NMR (500 MHz, $CDCl_3$)

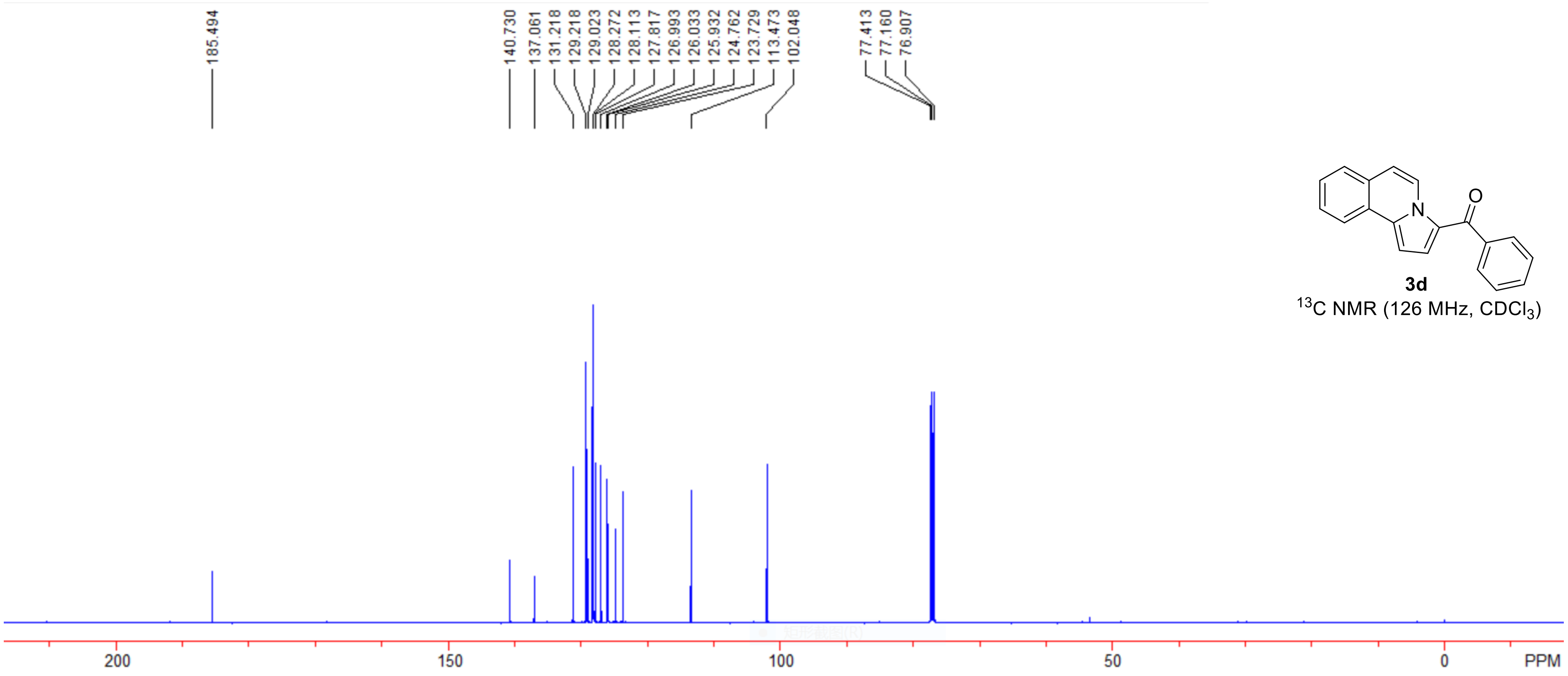

3d

^{13}C NMR (126 MHz, $CDCl_3$)

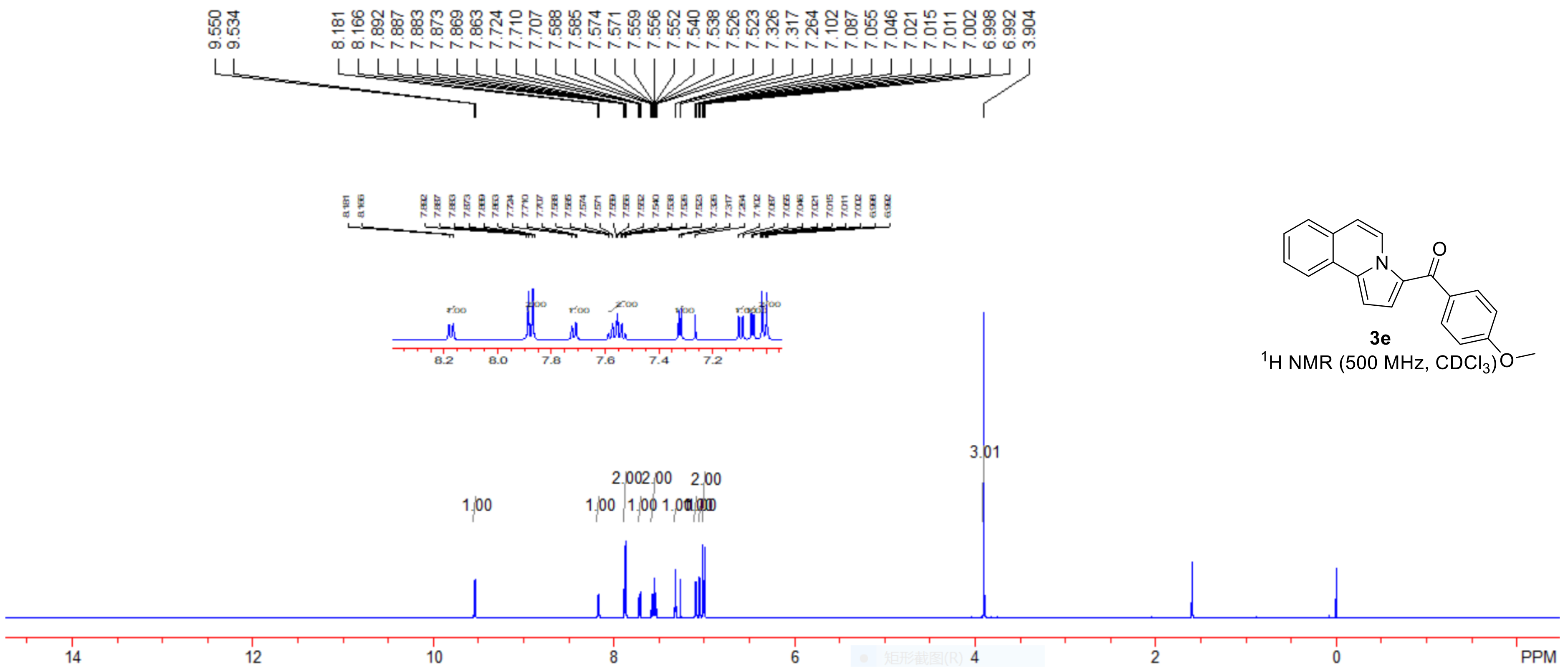
3e
^{1}H NMR (500 MHz, $CDCl_3$)
PPM

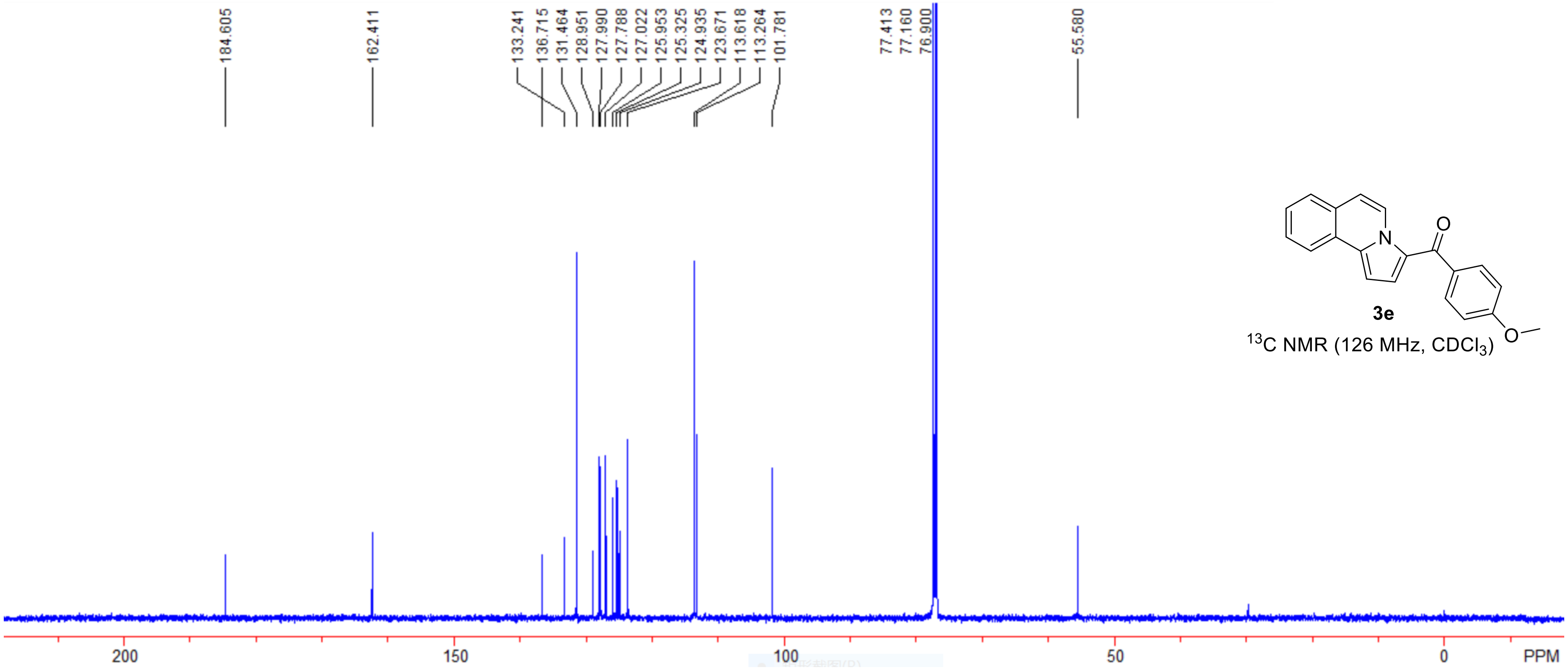
184.605
162.411
133.241
136.715
131.464
128.951
127.990
127.788
127.022
125.953
125.325
124.935
123.671
113.618
113.264
101.781
77.413
77.160
76.900
55.580
3e
^{13}C NMR (126 MHz, $CDCl_3$)
200
150
100
50
0
PPM

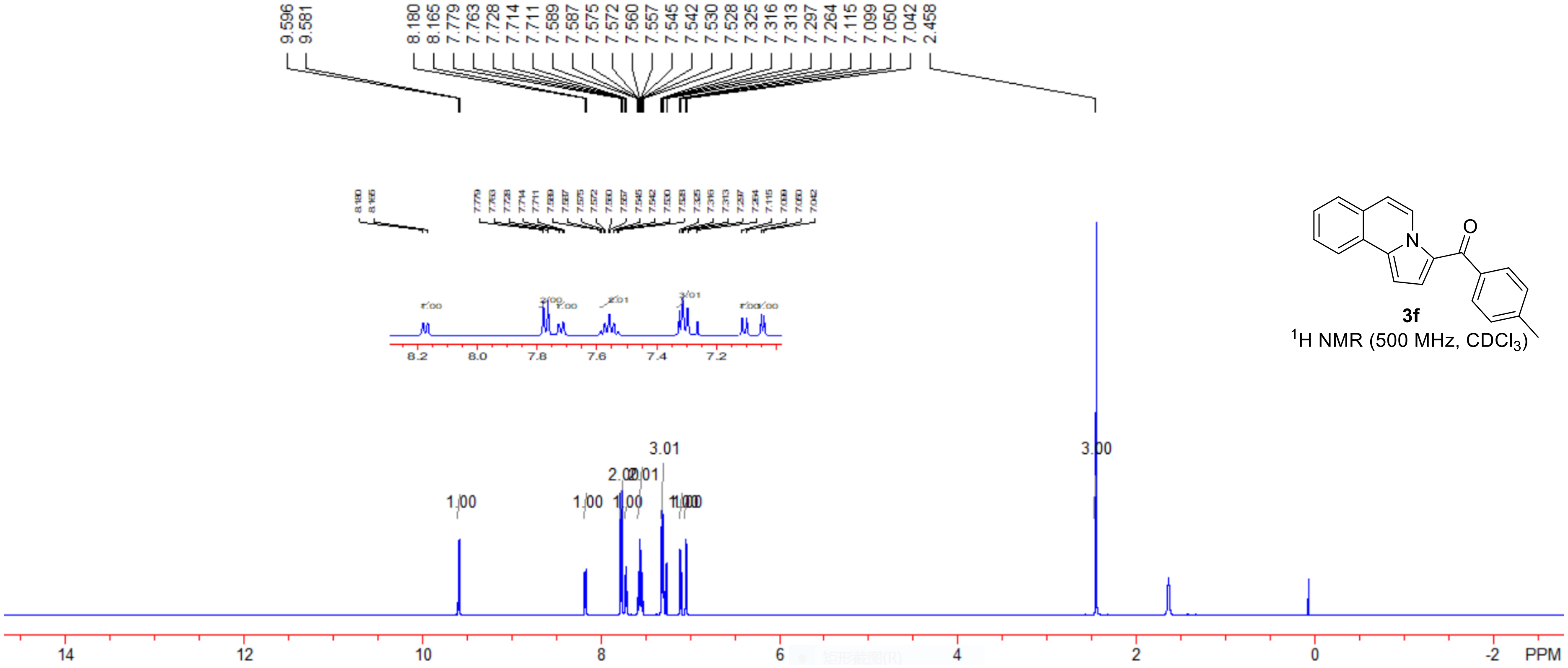
9.596
9.581
8.180
8.165
7.779
7.763
7.728
7.714
7.711
7.589
7.587
7.575
7.572
7.560
7.557
7.545
7.542
7.530
7.528
7.325
7.316
7.313
7.297
7.264
7.115
7.099
7.050
7.042
2.458
3f
^{1}H NMR (500 MHz, CDCl$_3$)
PPM

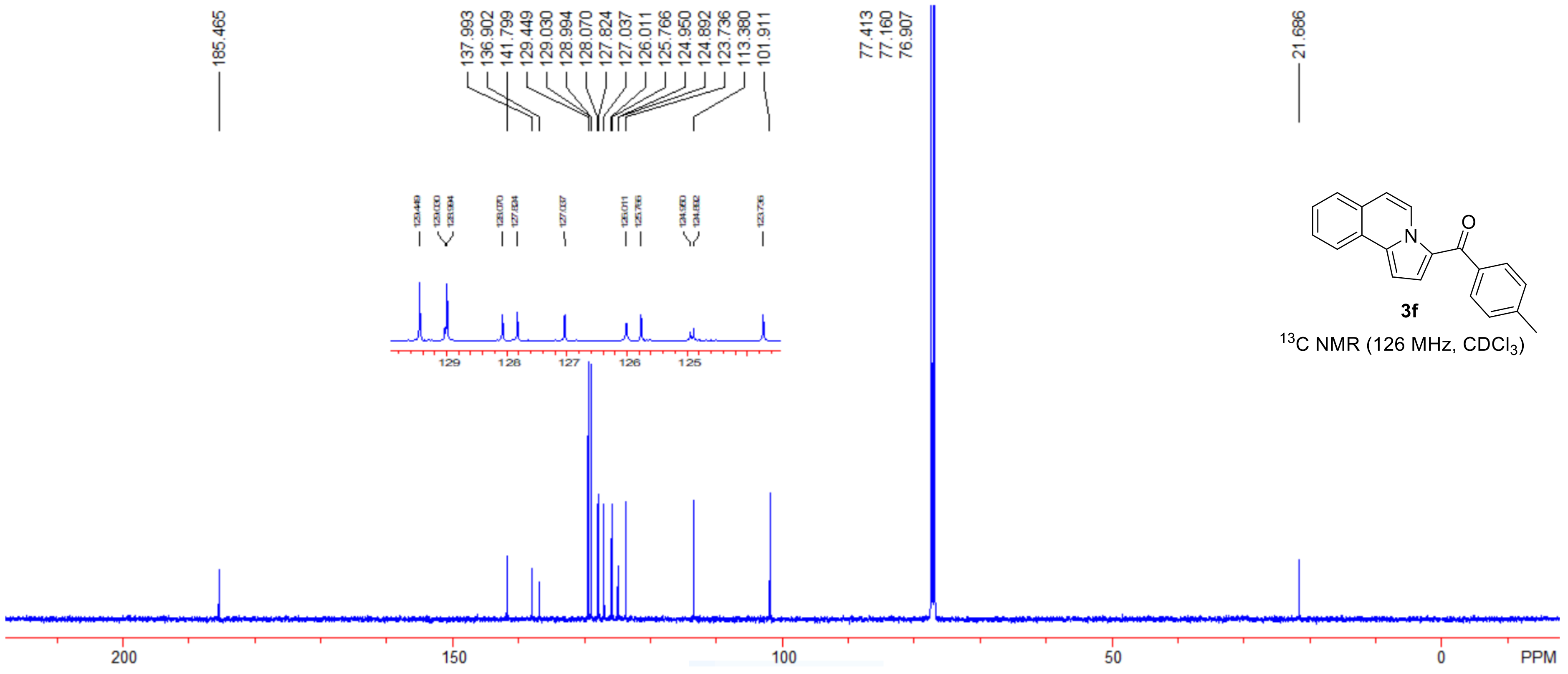
185.465
137.993
136.902
141.799
129.449
129.030
128.994
128.070
127.824
127.037
126.011
125.766
124.950
124.892
123.736
113.380
101.911
77.413
77.160
76.907
21.686
3f
^{13}C NMR (126 MHz, $CDCl_3$)
200
150
100
50
0
PPM

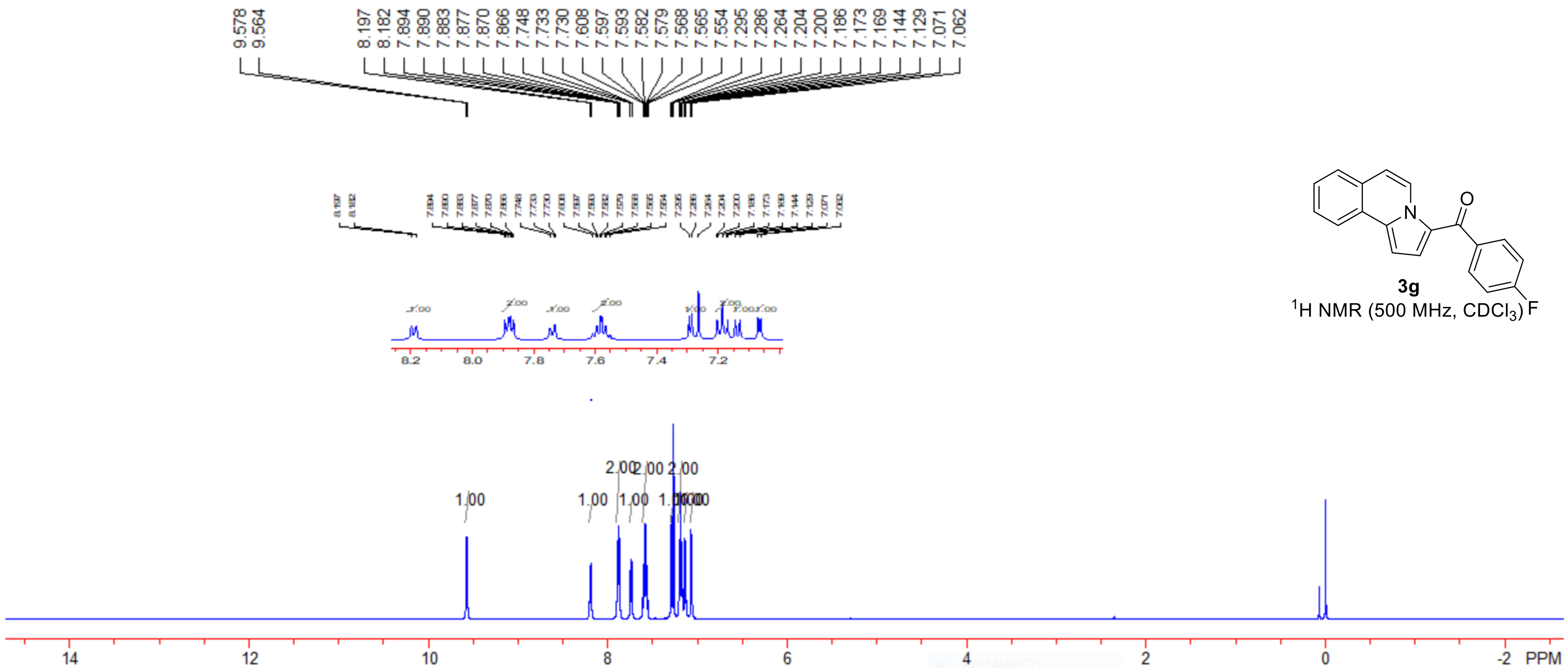

3g

^{1}H NMR (500 MHz, $CDCl_3$)

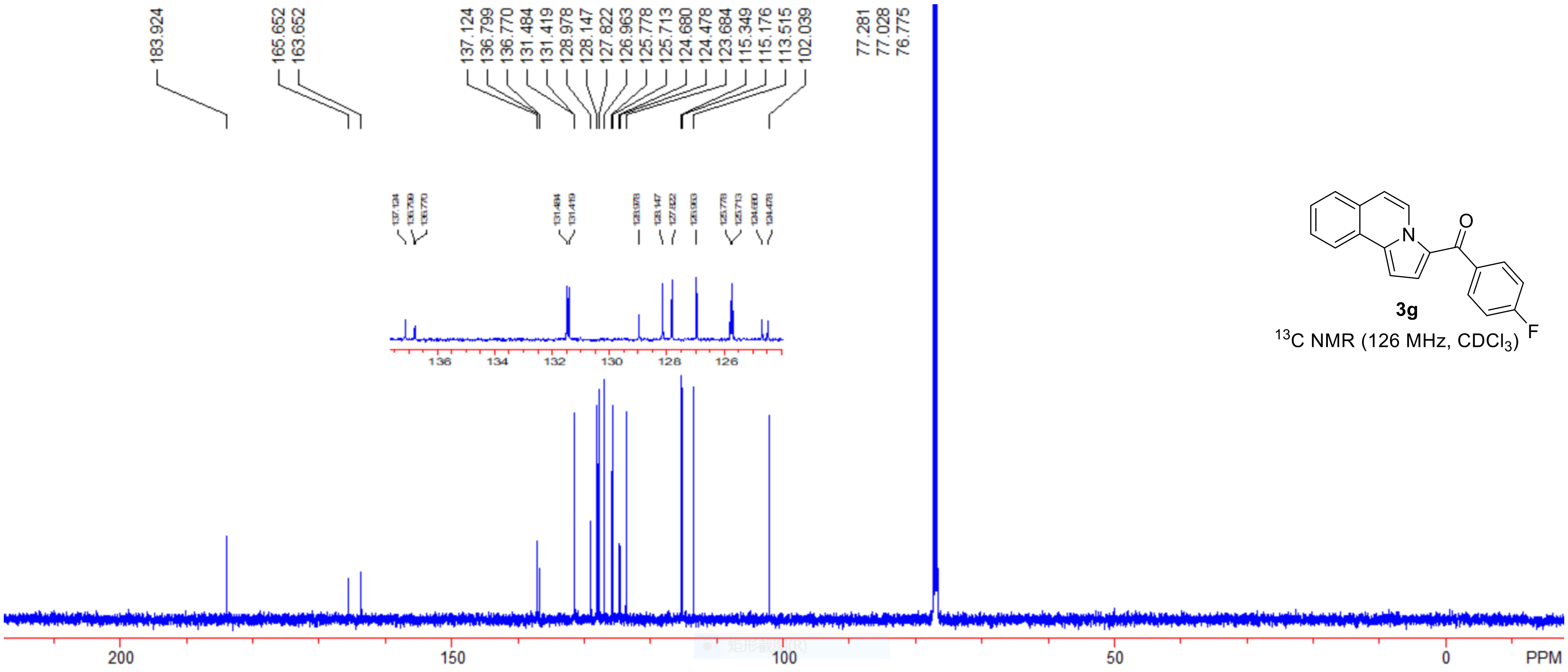

3g

^{13}C NMR (126 MHz, $CDCl_3$)

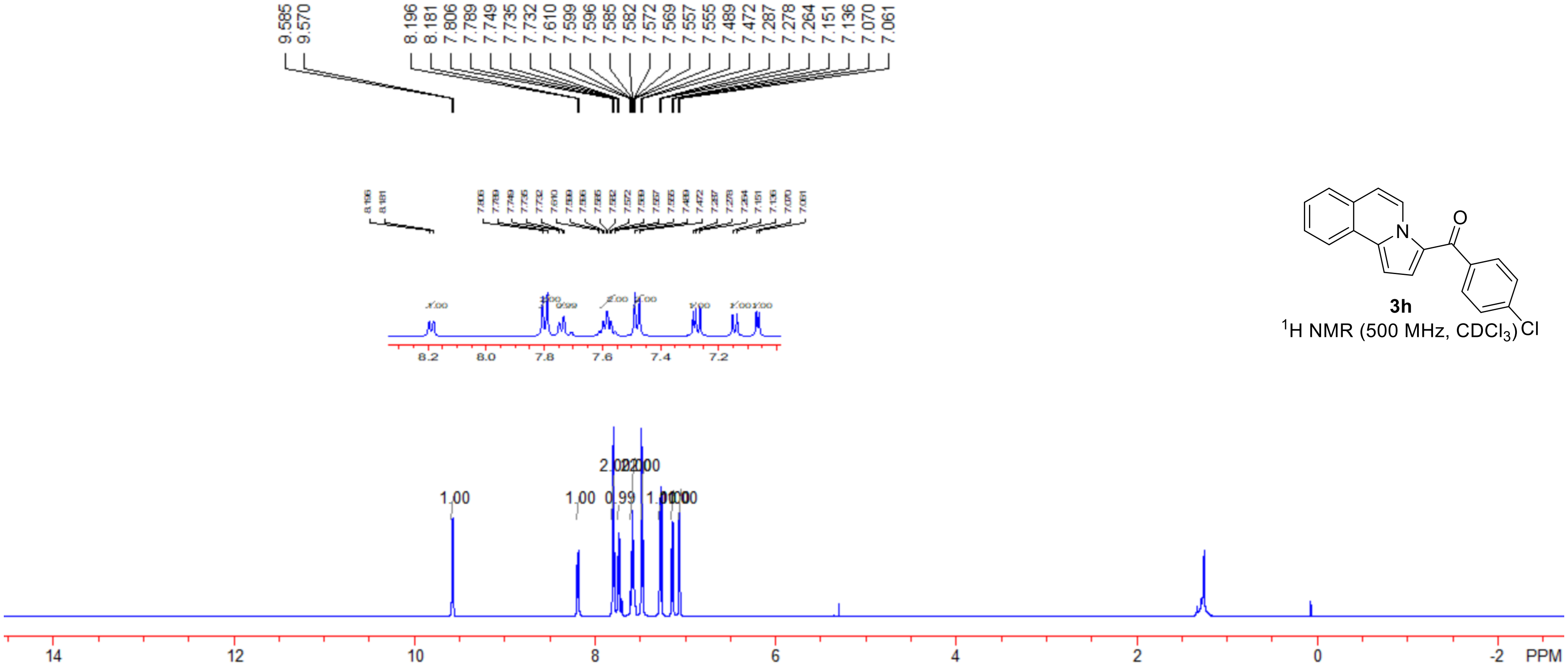

3h

^{1}H NMR (500 MHz, $CDCl_3$)

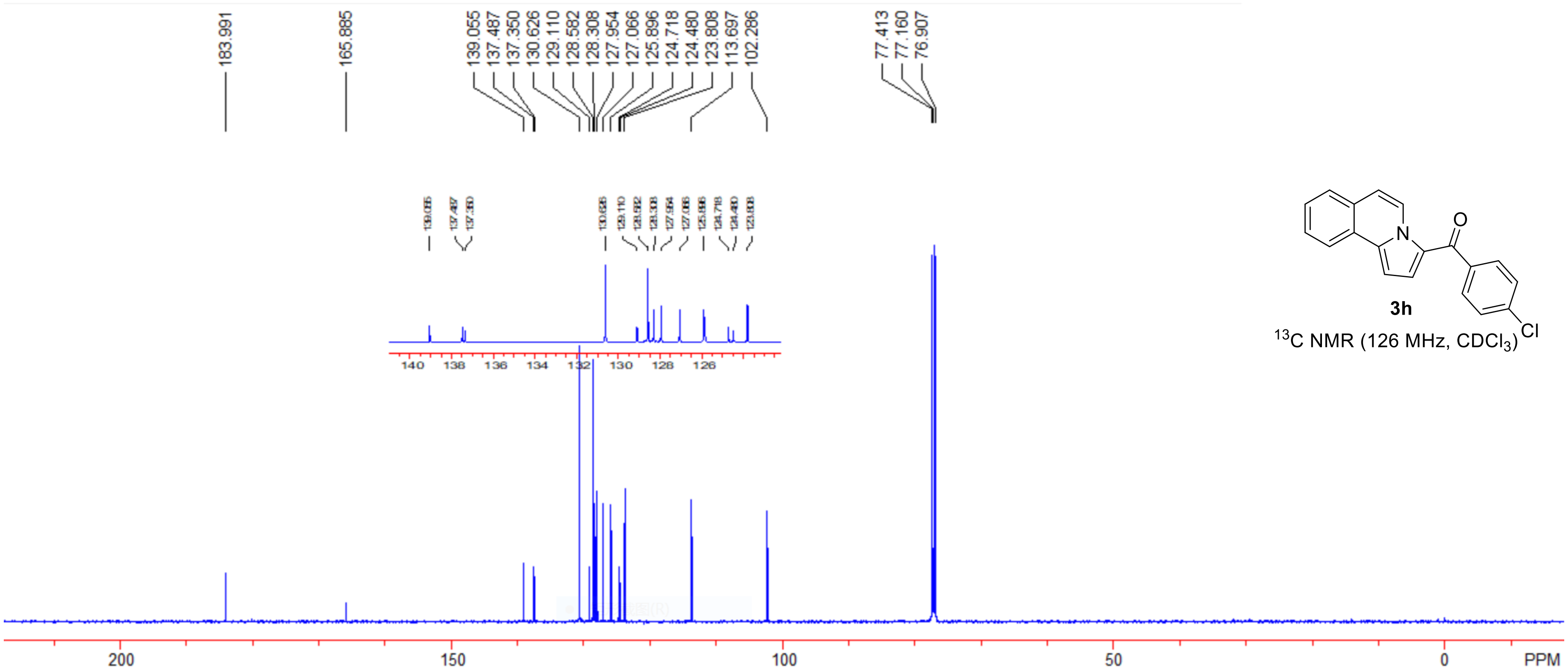

3h

^{13}C NMR (126 MHz, $CDCl_3$)

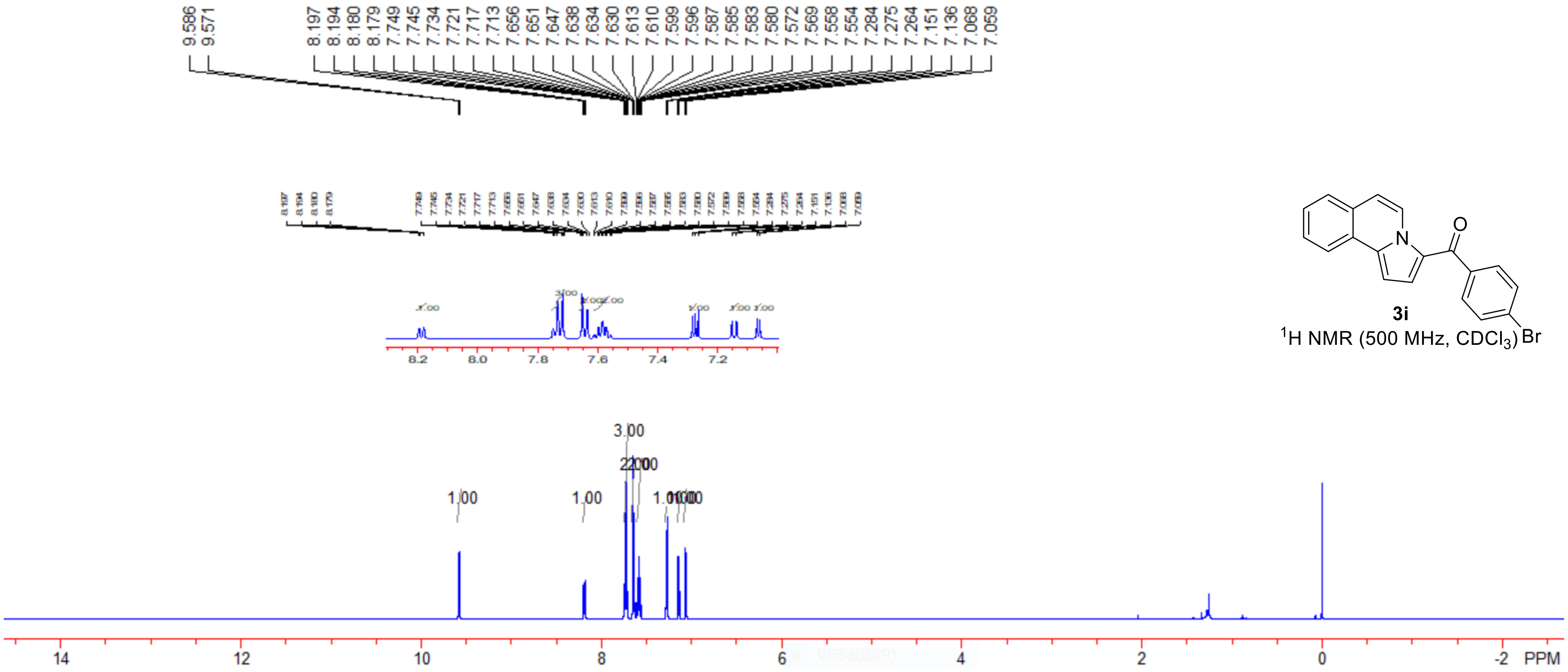

3i

^{1}H NMR (500 MHz, $CDCl_3$)

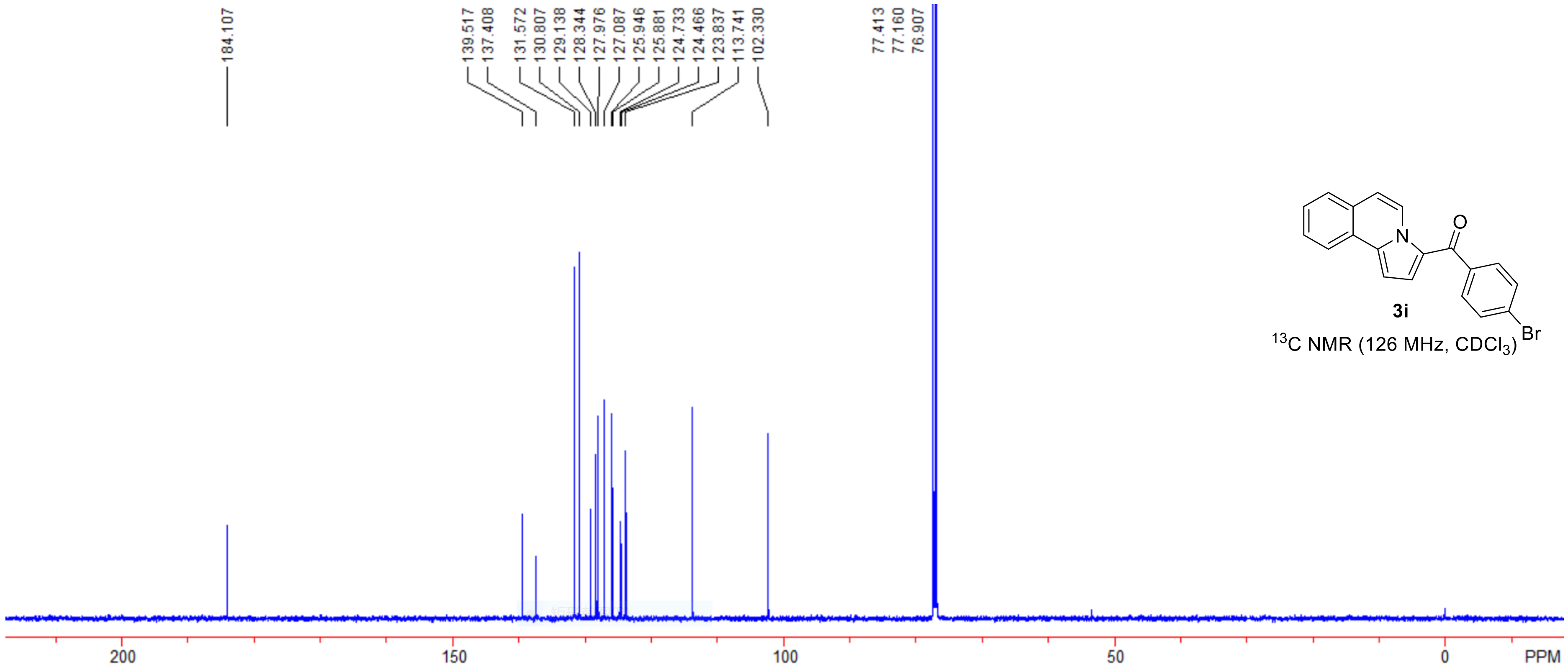

3i

^{13}C NMR (126 MHz, $CDCl_3$)

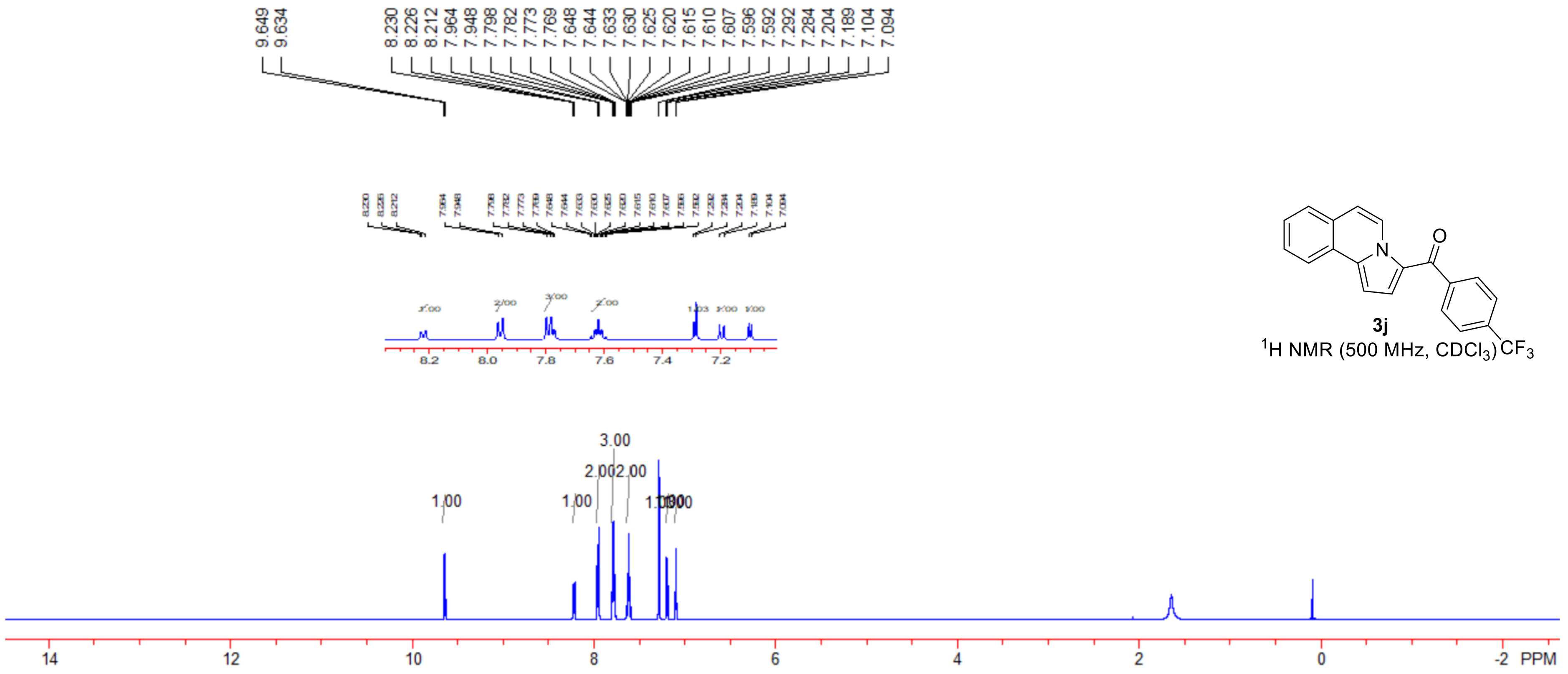
9.649
9.634
8.230
8.226
8.212
7.964
7.948
7.798
7.782
7.773
7.769
7.648
7.644
7.633
7.630
7.625
7.620
7.615
7.610
7.607
7.596
7.592
7.292
7.284
7.204
7.189
7.104
7.094
3j
^{1}H NMR (500 MHz, CDCl$_3$)
CF$_3$
PPM

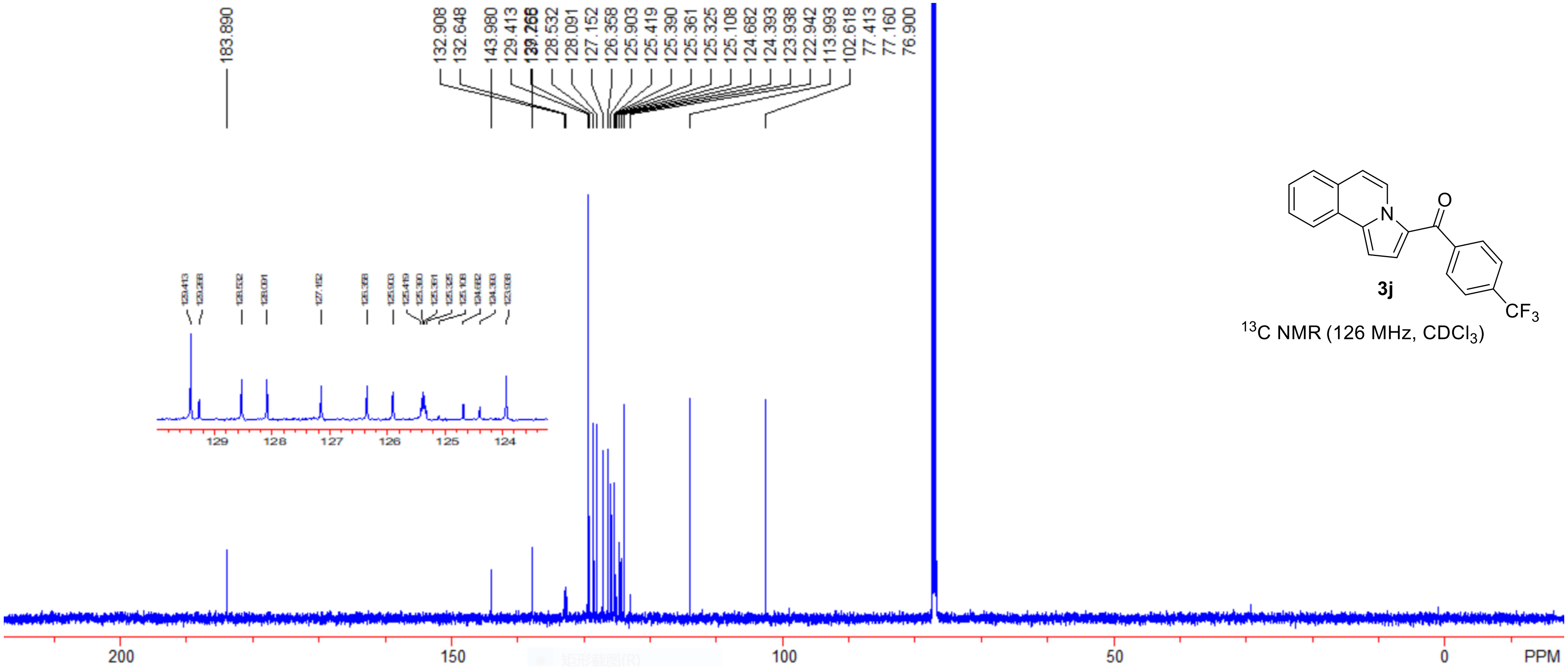

3j

^{13}C NMR (126 MHz, $CDCl_3$)

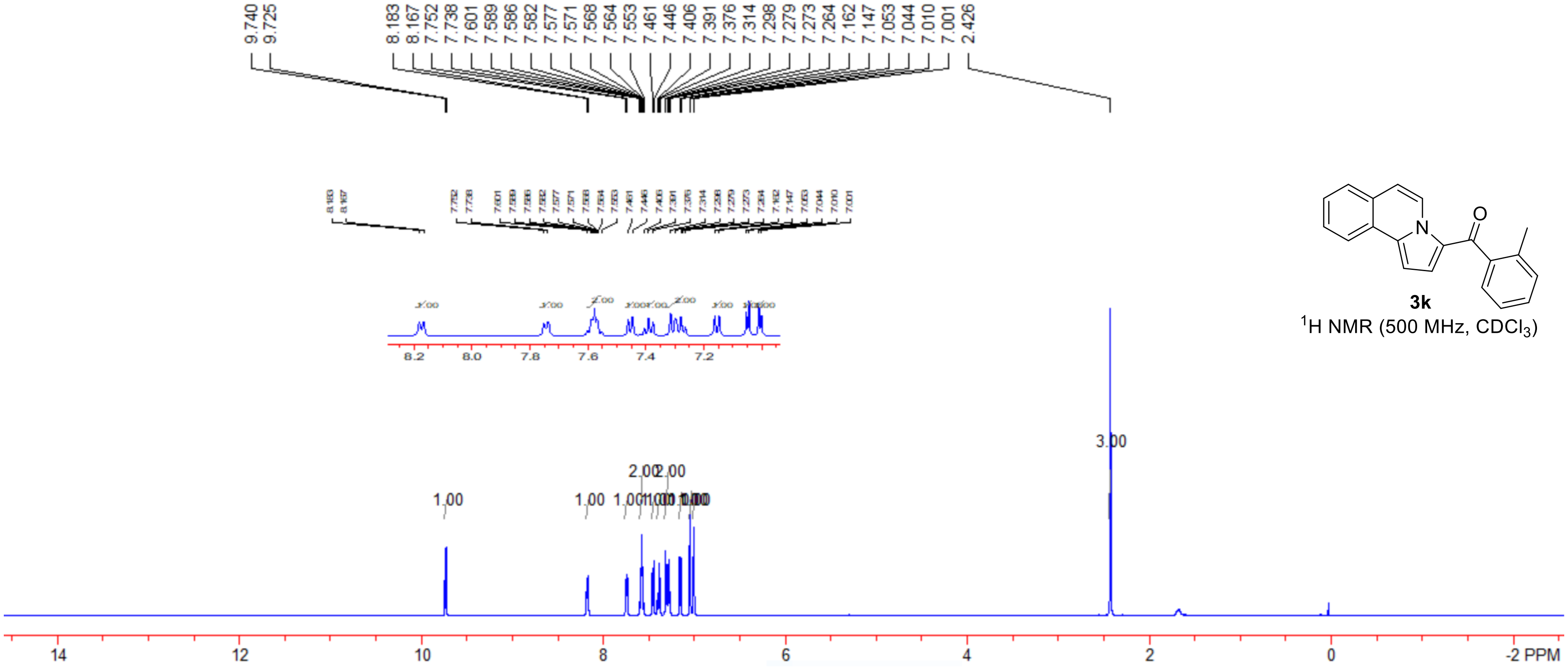

3k

^{1}H NMR (500 MHz, $CDCl_3$)

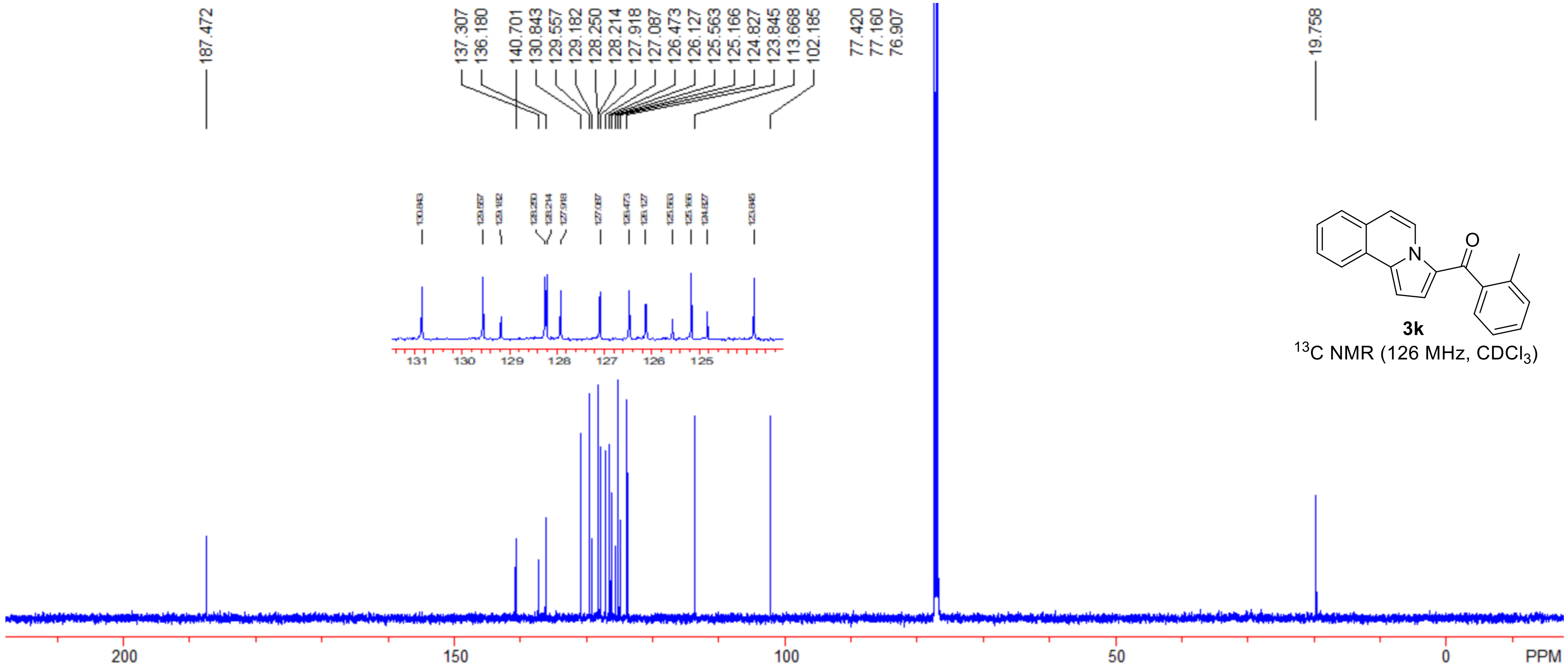

3k

^{13}C NMR (126 MHz, $CDCl_3$)

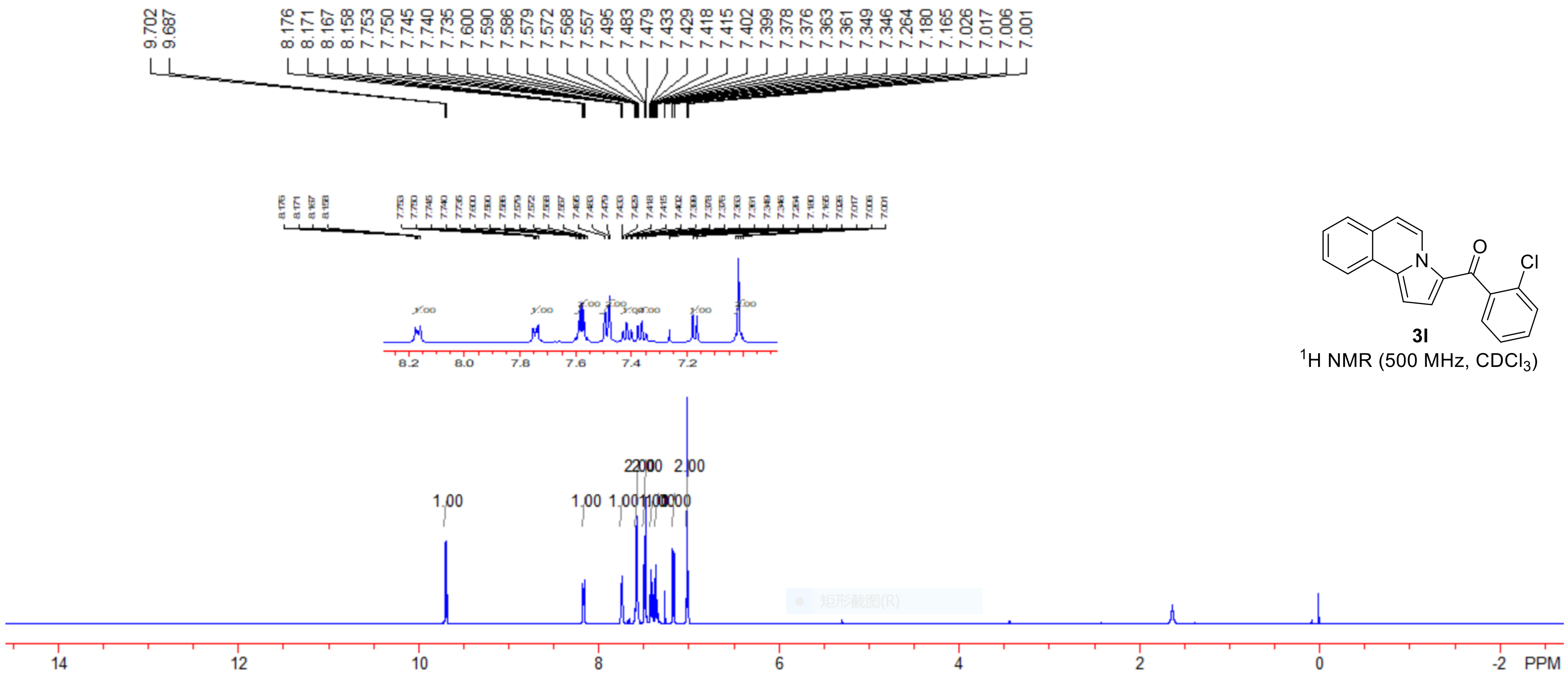

3l

^1H NMR (500 MHz, $CDCl_3$)

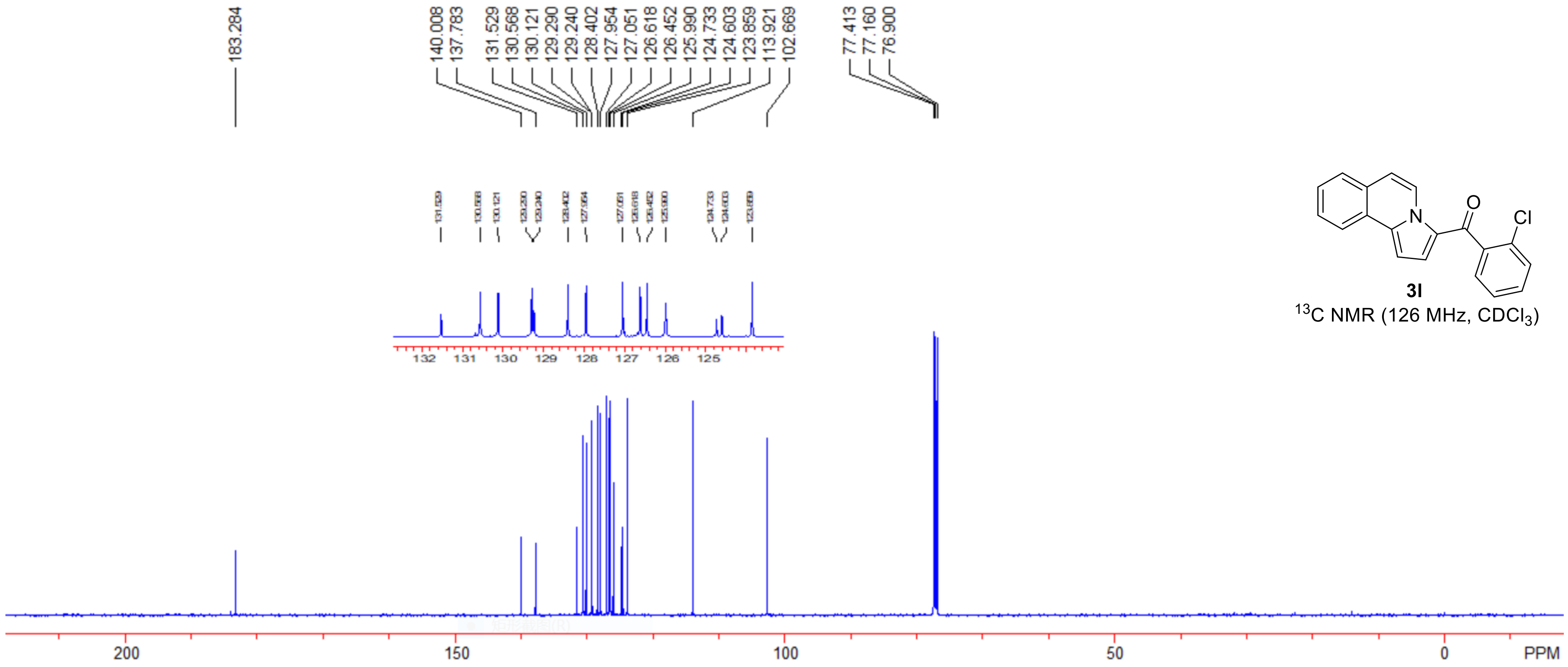

3l

^{13}C NMR (126 MHz, $CDCl_3$)

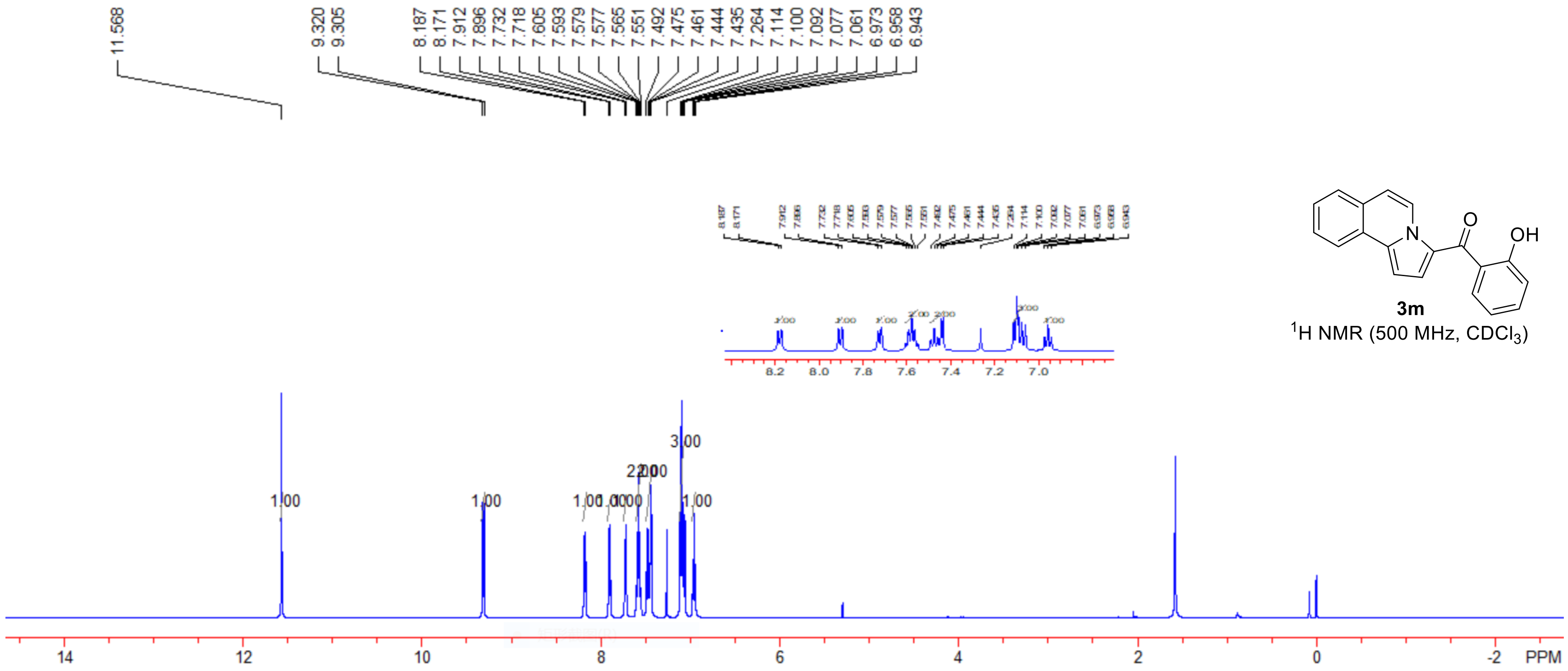

3m

^{1}H NMR (500 MHz, $CDCl_3$)

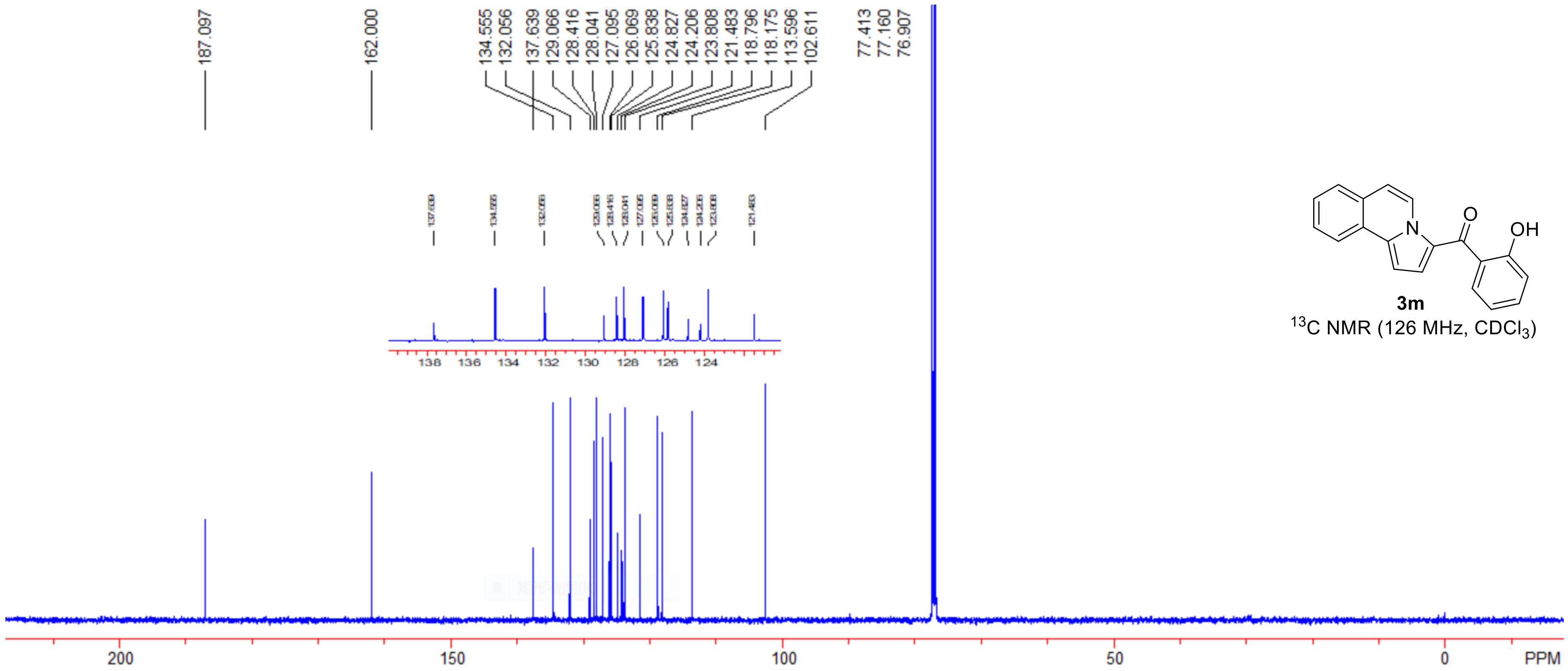

3m

^{13}C NMR (126 MHz, $CDCl_3$)

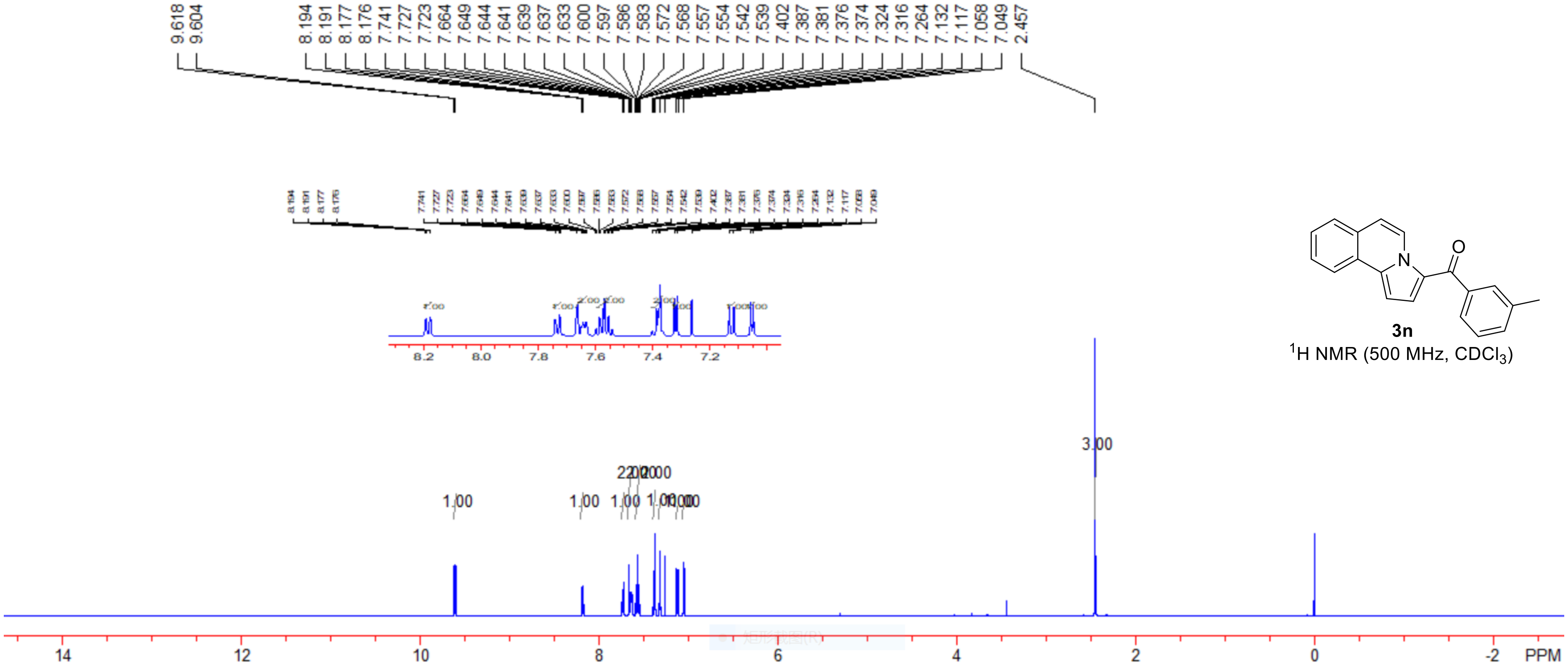

3n

^{1}H NMR (500 MHz, $CDCl_3$)

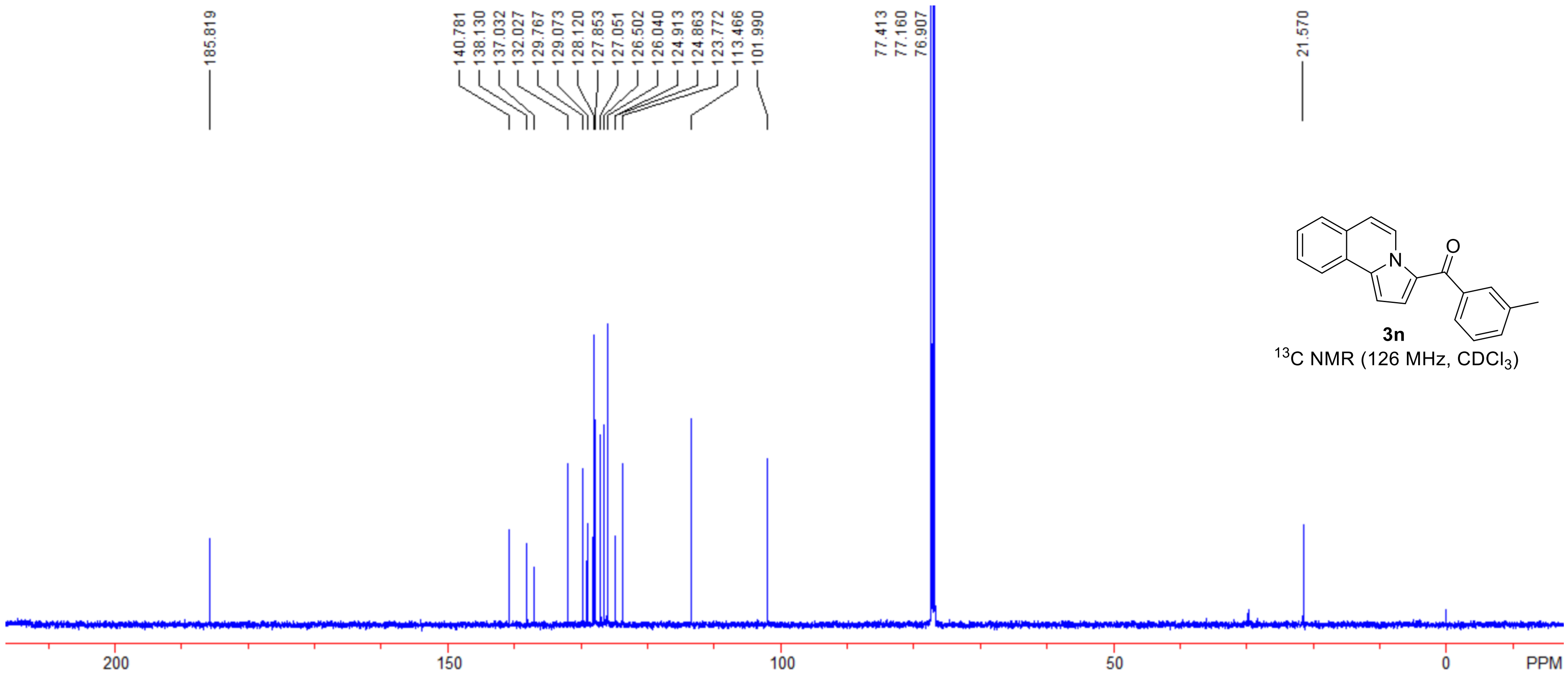
185.819
140.781
138.130
137.032
132.027
129.767
129.073
128.120
127.853
127.051
126.502
126.040
124.913
124.863
123.772
113.466
101.990
77.413
77.160
76.907
21.570
O
N
3n
^{13}C NMR (126 MHz, $CDCl_3$)
200
150
100
50
0
PPM

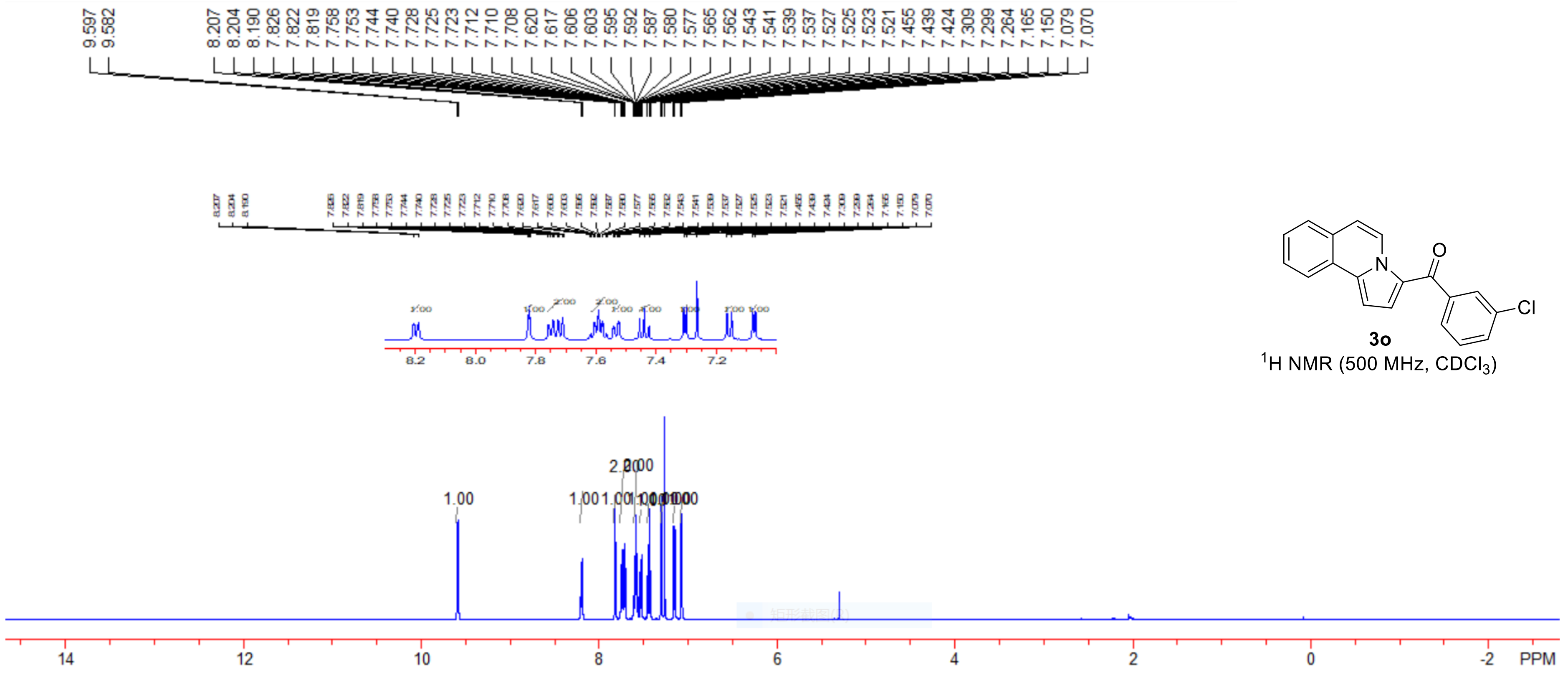

3o

^{1}H NMR (500 MHz, $CDCl_3$)

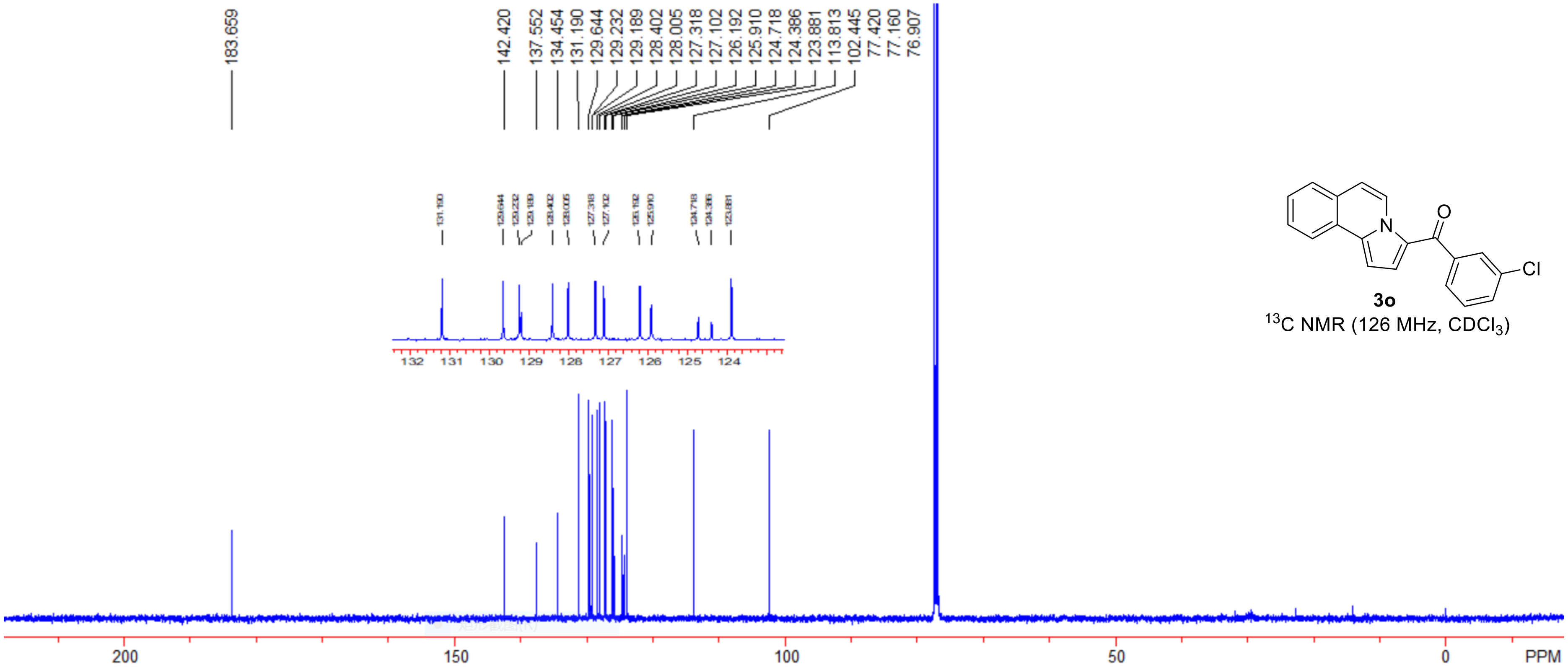

3o

^{13}C NMR (126 MHz, $CDCl_3$)

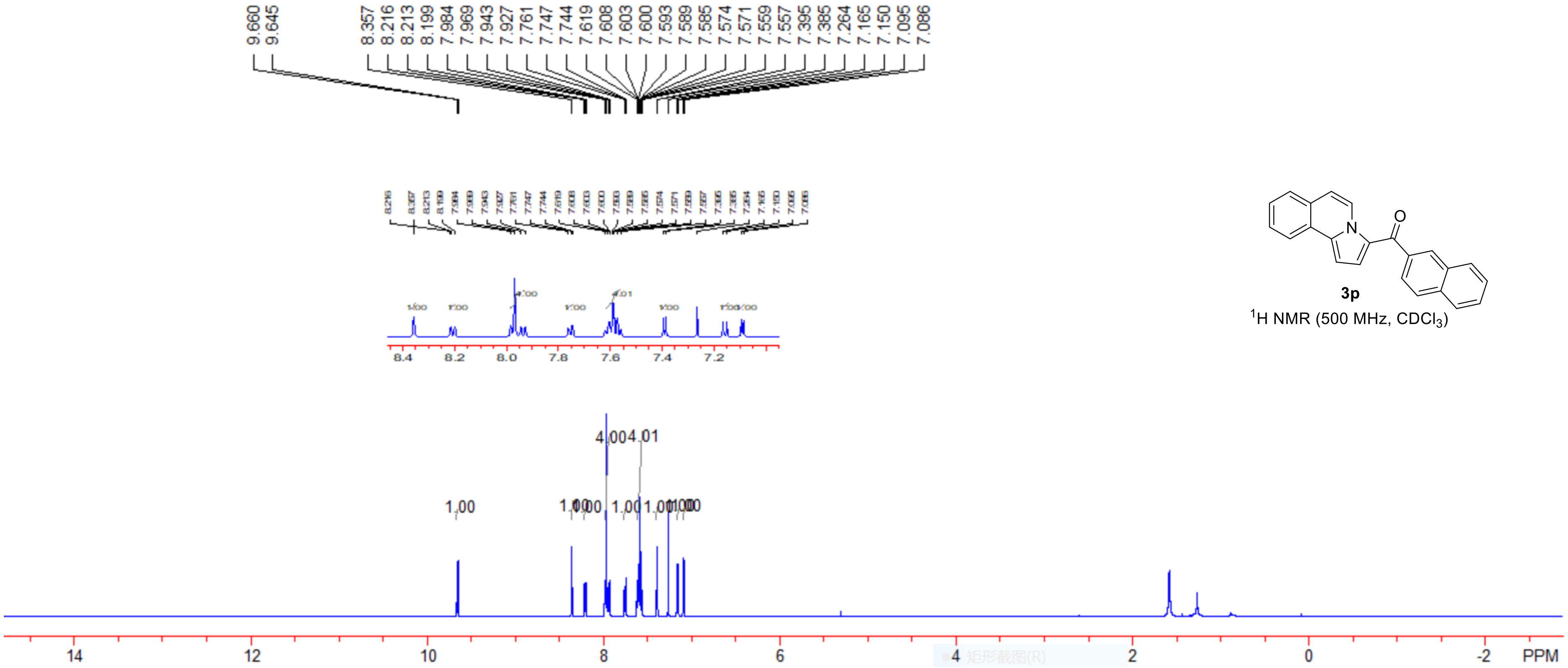

3p

^{1}H NMR (500 MHz, $CDCl_3$)

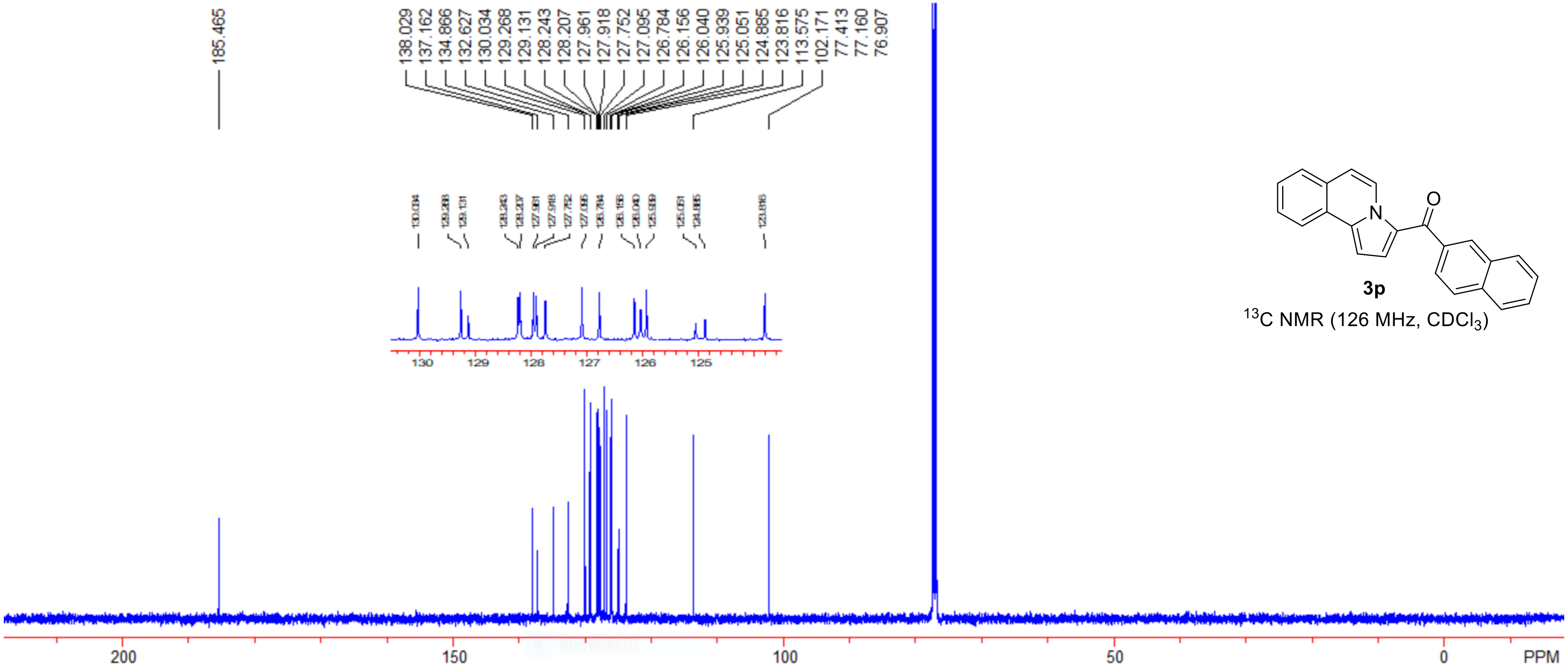

3p

^{13}C NMR (126 MHz, $CDCl_3$)

The purity of compounds **3a–3q** by HPLC.

Compounds	RT (min)	Area (%)
**3a**	9.058	98.84
**3b**	9.694	98.02
**3c**	8.676	99.89
**3d**	7.195	99.99
**3e**	9.083	98.81
**3f**	8.030	95.08
**3g**	9.509	99.99
**3h**	9.130	98.90
**3i**	8.508	99.99
**3j**	8.991	98.19
**3k**	7.709	99.85
**3l**	8.012	99.83
**3m**	8.491	98.51
**3n**	8.030	99.84
**3o**	9.175	99.38
**3p**	9.439	98.65

3a

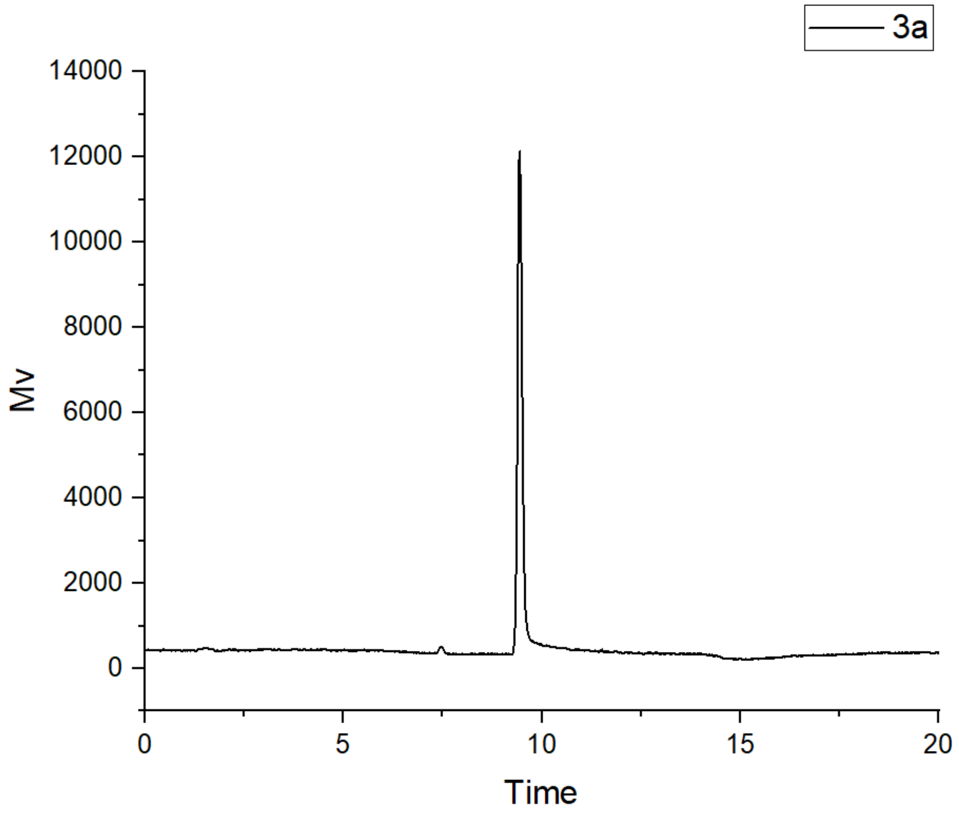
3a
14000
12000
10000
8000
6000
4000
2000
0
Mv
0
5
10
15
20
Time

3b

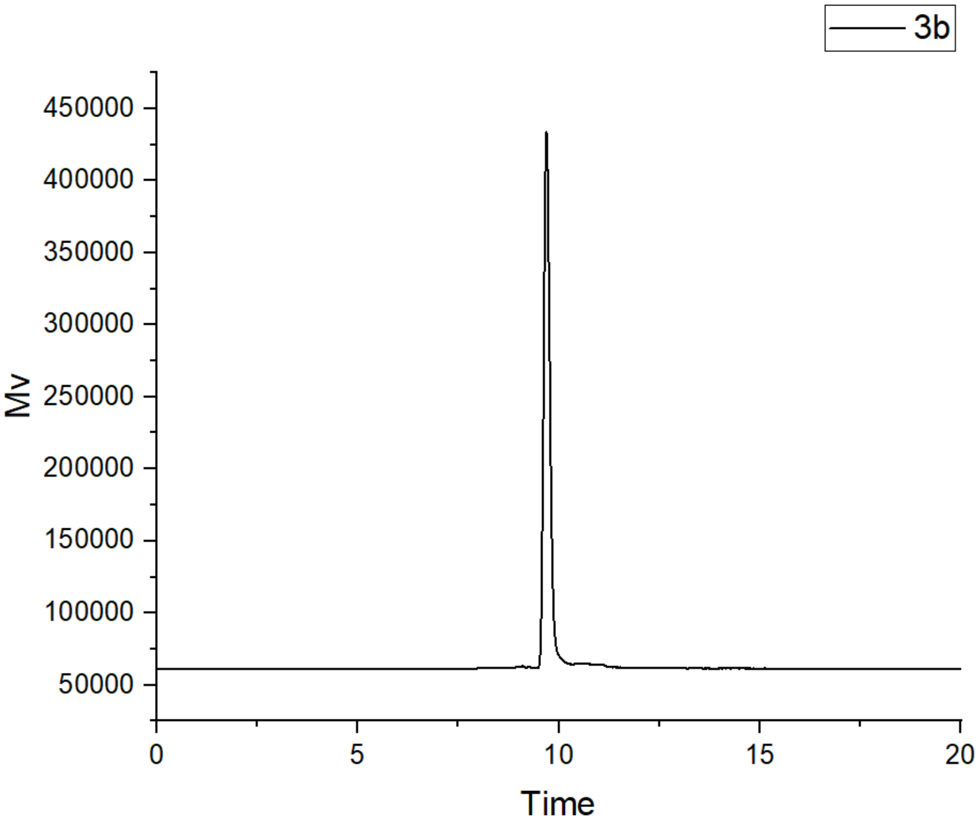
3b
450000
400000
350000
300000
250000
200000
150000
100000
50000
Mv
0
5
10
15
20
Time

3c

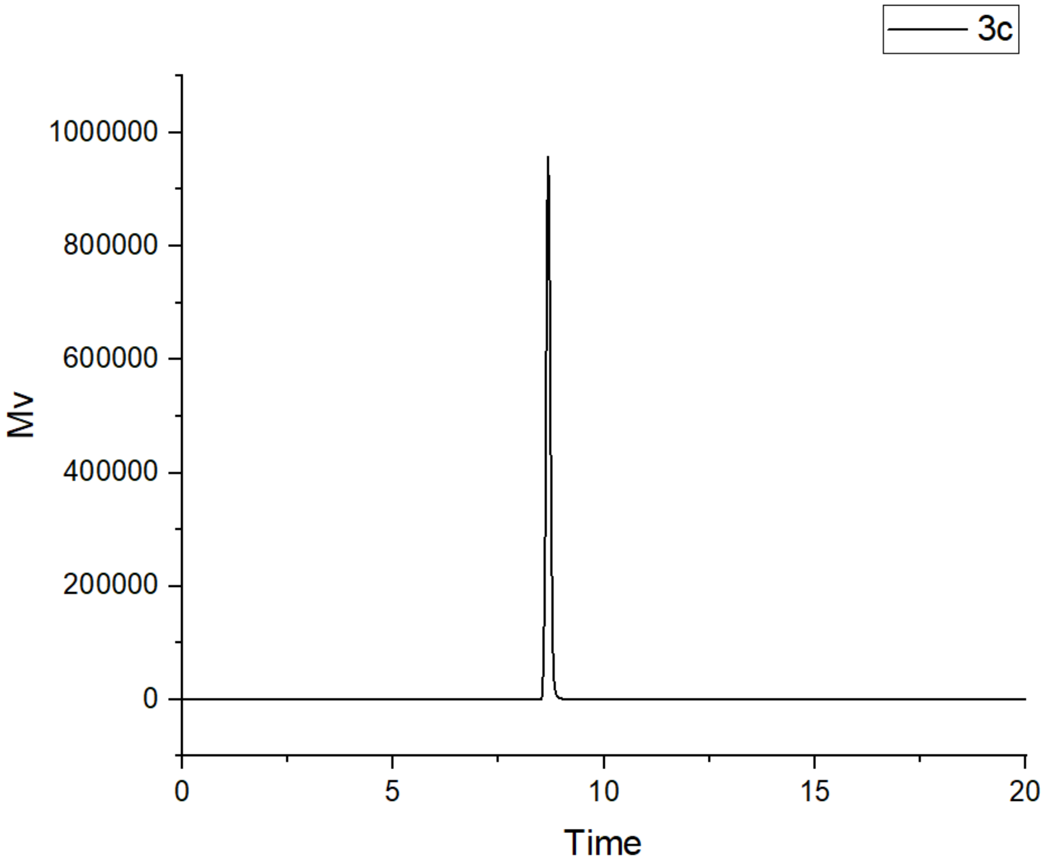
3c
1000000
800000
600000
400000
200000
0
Mv
0
5
10
15
20
Time

3d

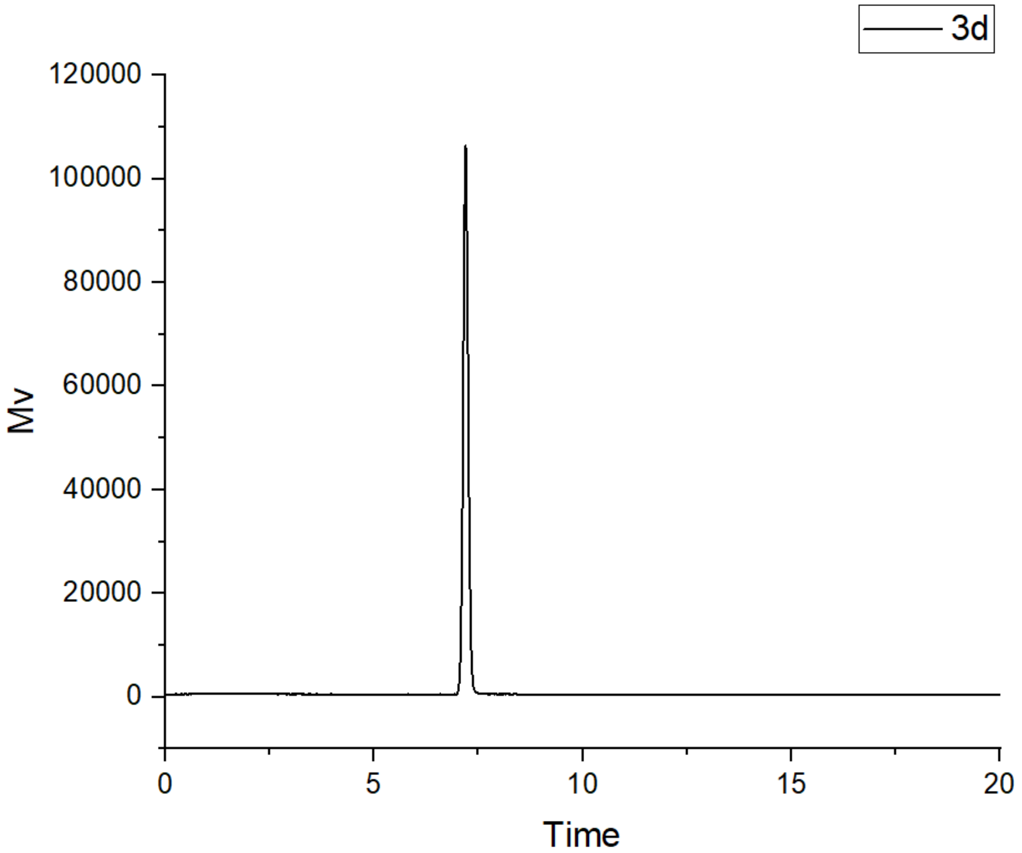
3d
120000
100000
80000
60000
40000
20000
0
Mv
0
5
10
15
20
Time

3e

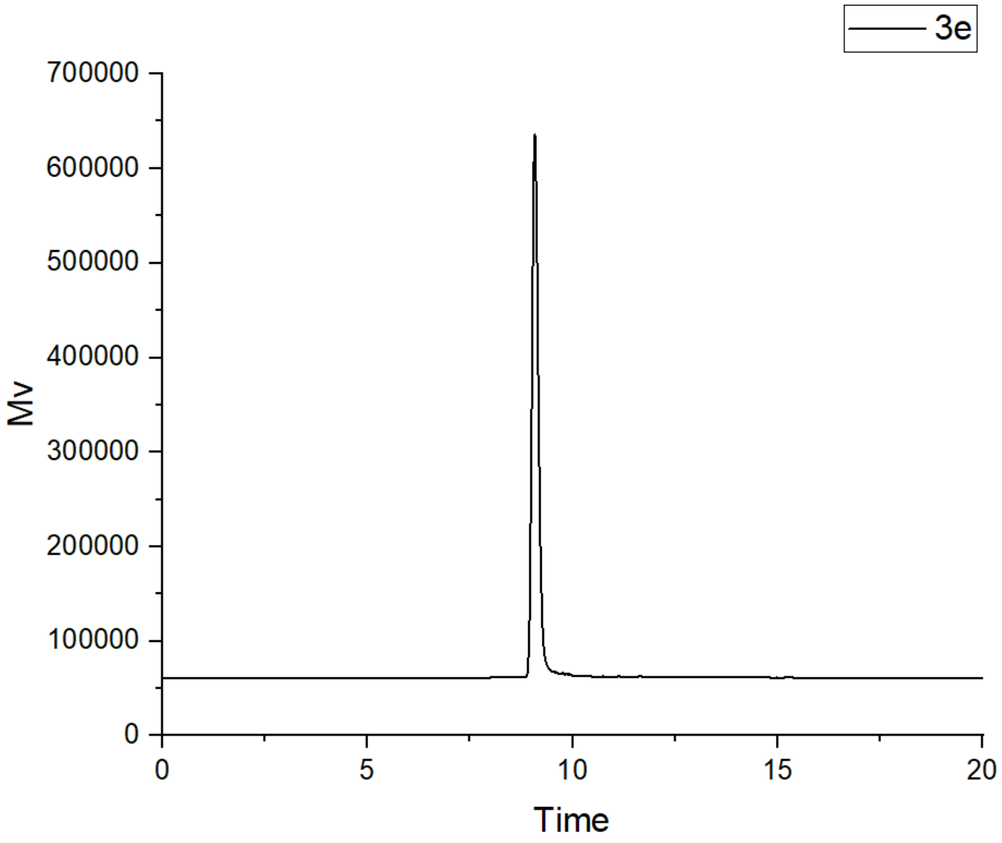

3e
700000
600000
500000
400000
300000
200000
100000
0
Mv
0
5
10
15
20
Time

3f

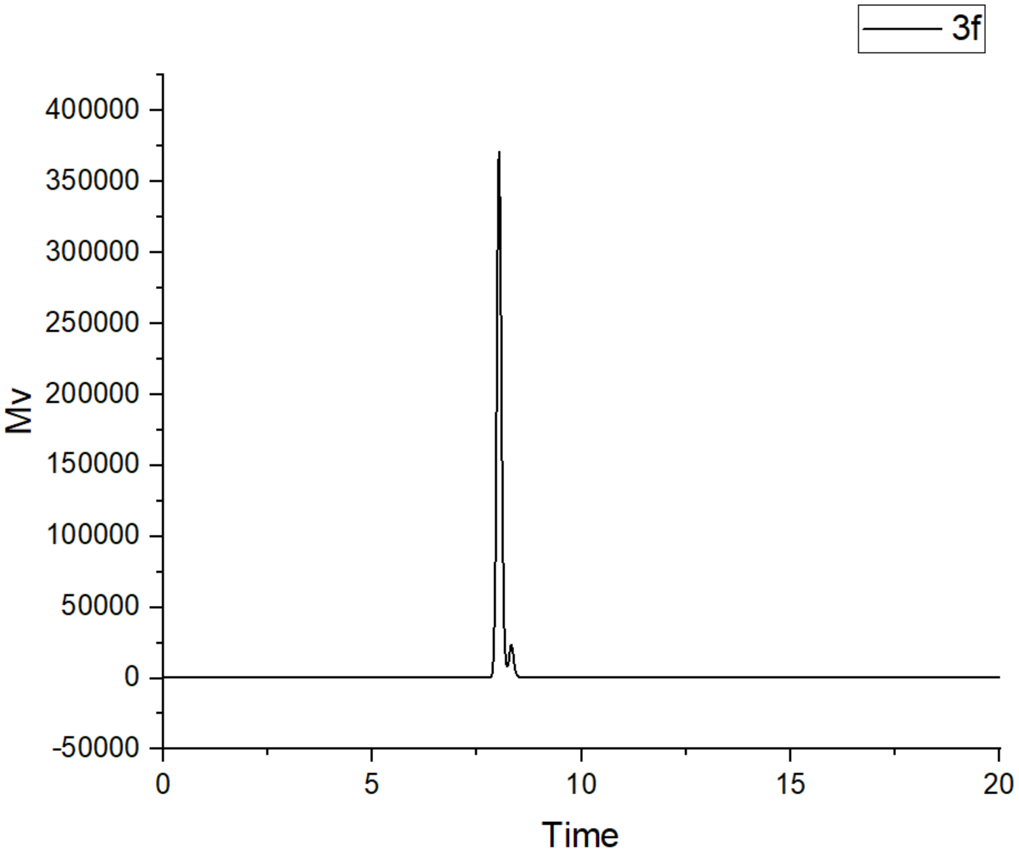

3f
400000
350000
300000
250000
200000
150000
100000
50000
0
-50000
Mv
0
5
10
15
20
Time

3g

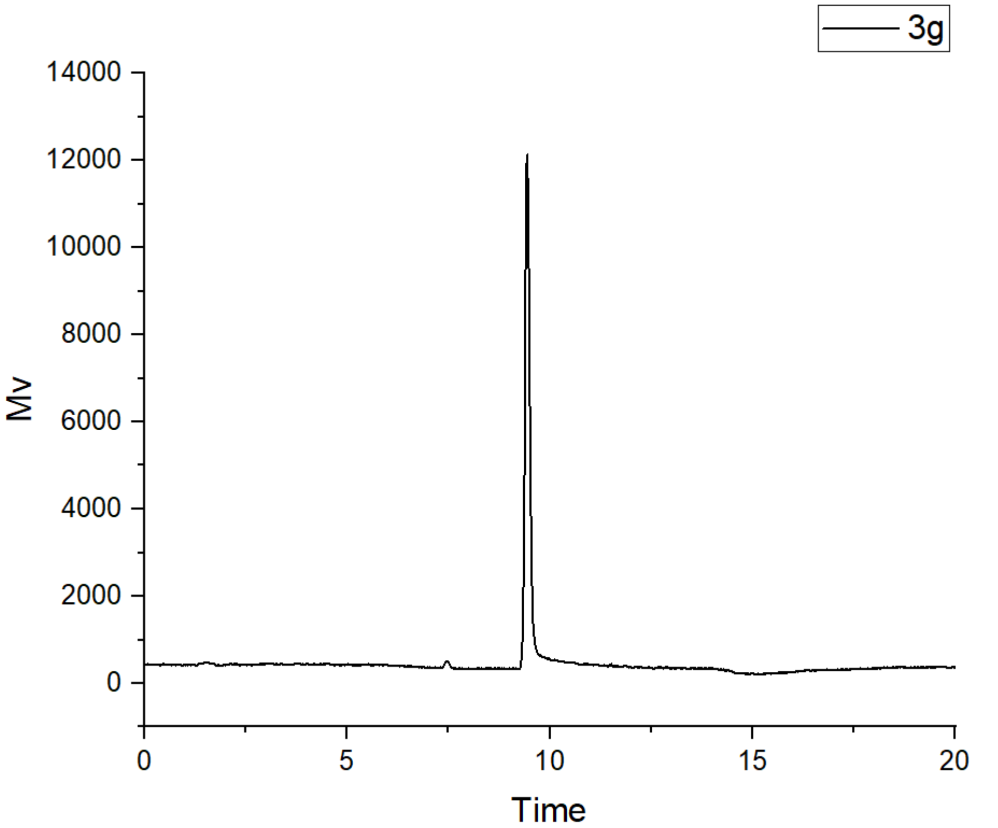
3g
14000
12000
10000
8000
6000
4000
2000
0
Mv
0
5
10
15
20
Time

3h

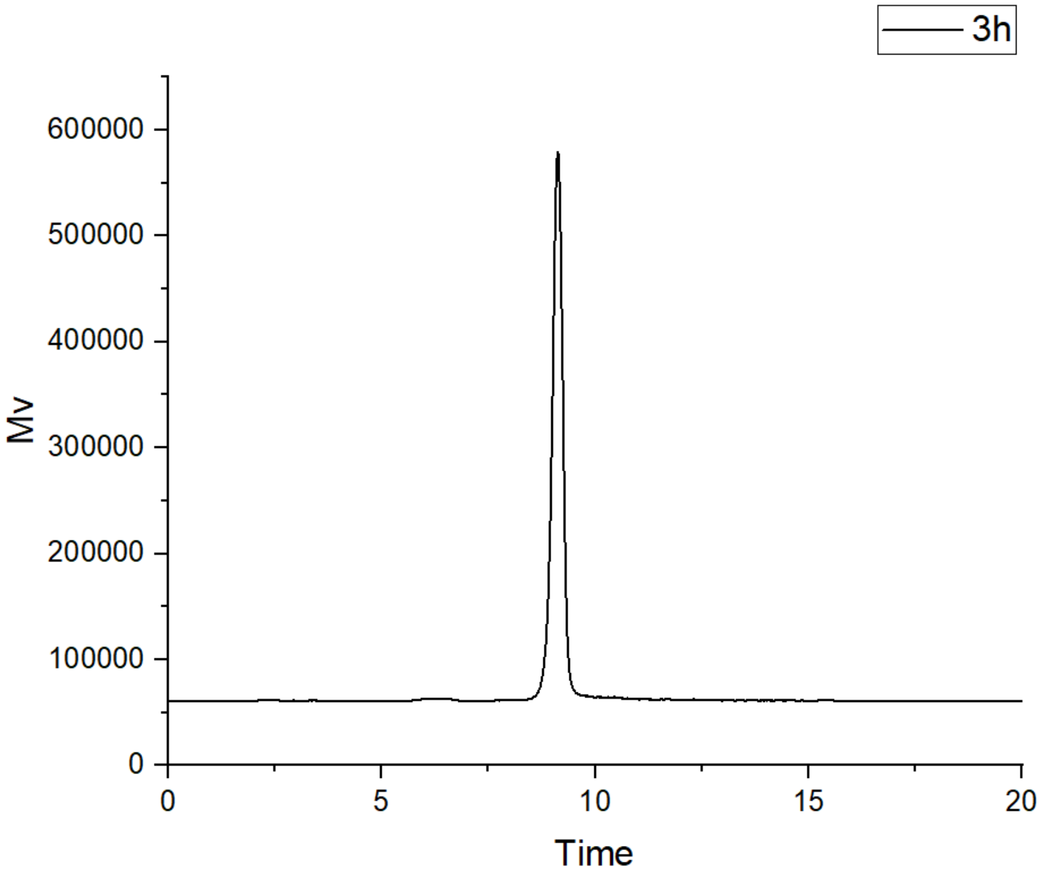
3h
600000
500000
400000
300000
200000
100000
0
Mv
0
5
10
15
20
Time

3i

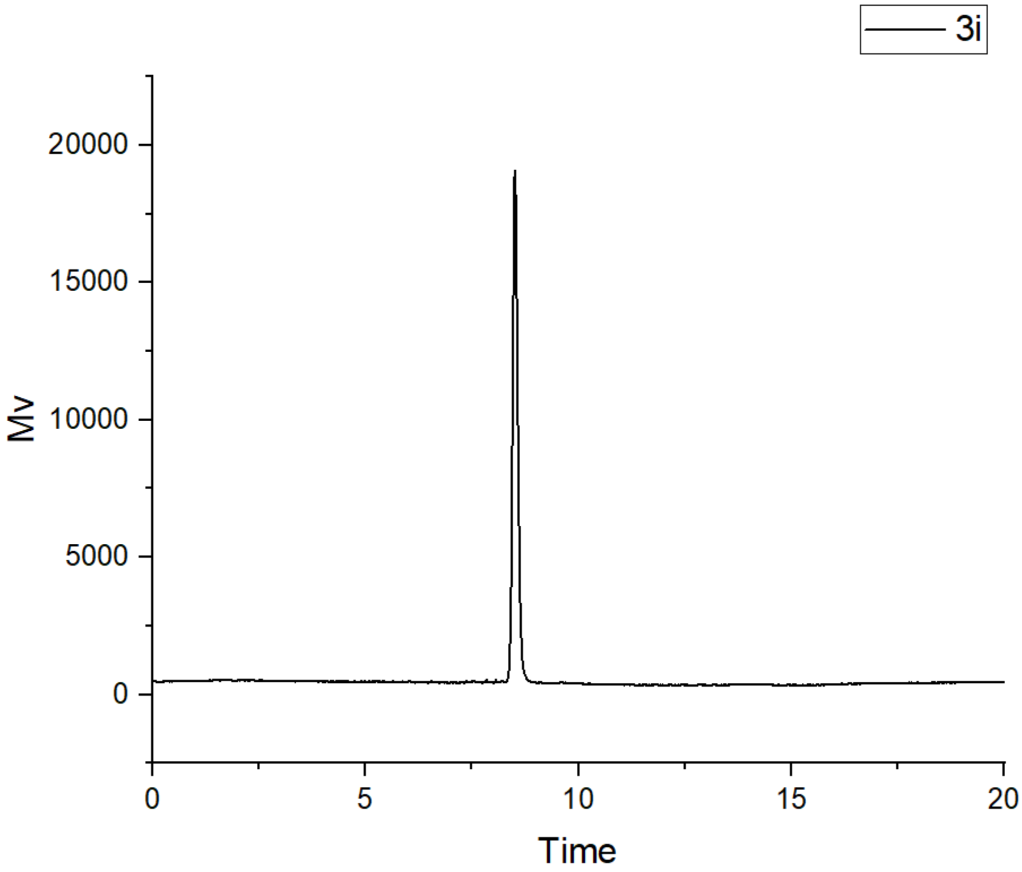
3i
20000
15000
10000
5000
0
Mv
0
5
10
15
20
Time

3j

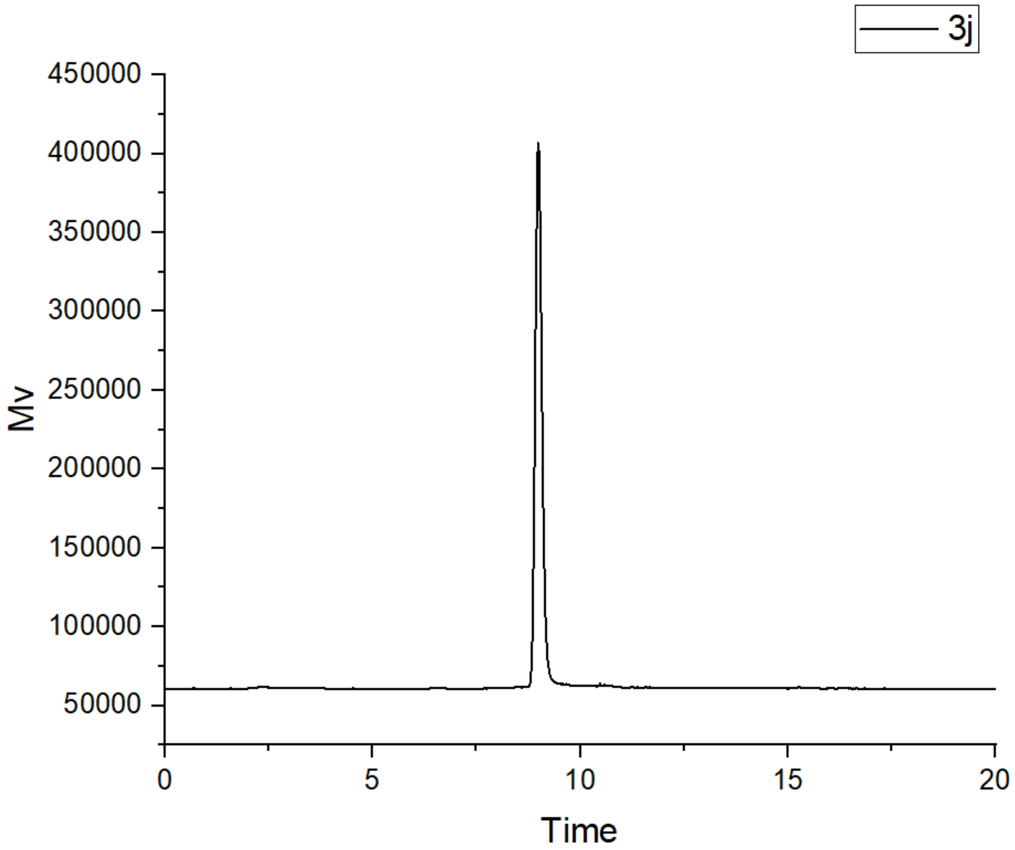
3j
450000
400000
350000
300000
250000
200000
150000
100000
50000
Mv
0
5
10
15
20
Time

3k

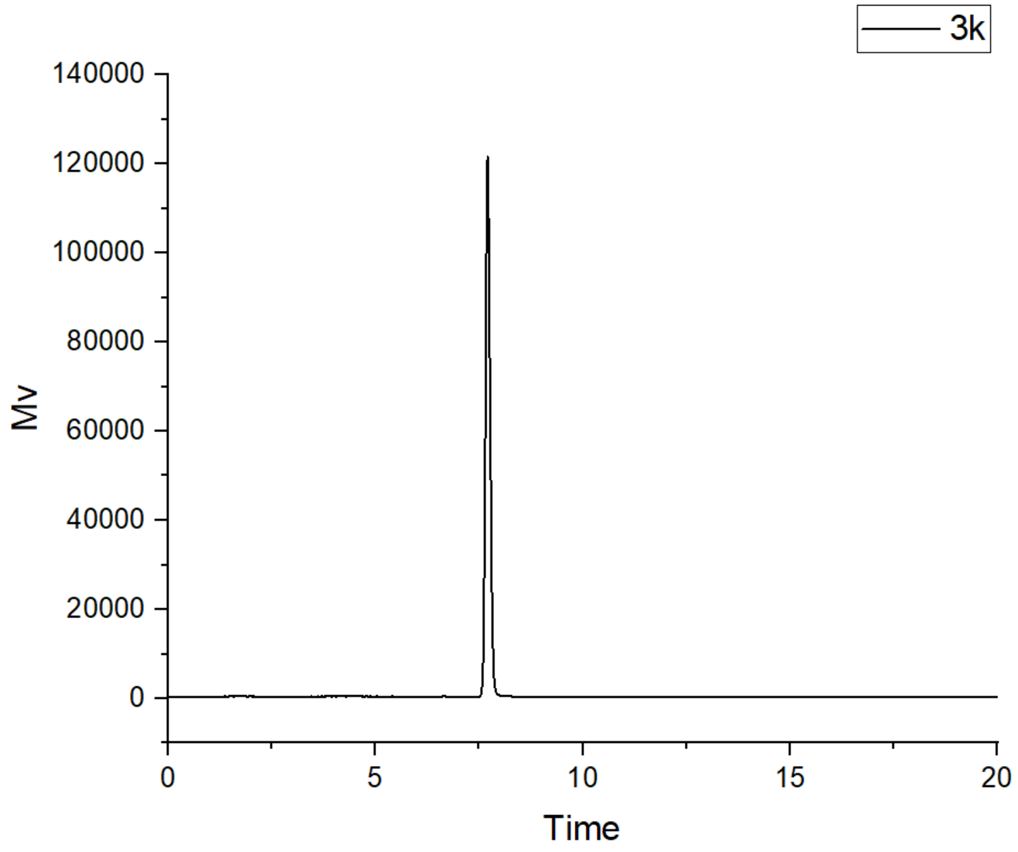
3k
140000
120000
100000
80000
60000
40000
20000
0
Mv
0
5
10
15
20
Time

3l

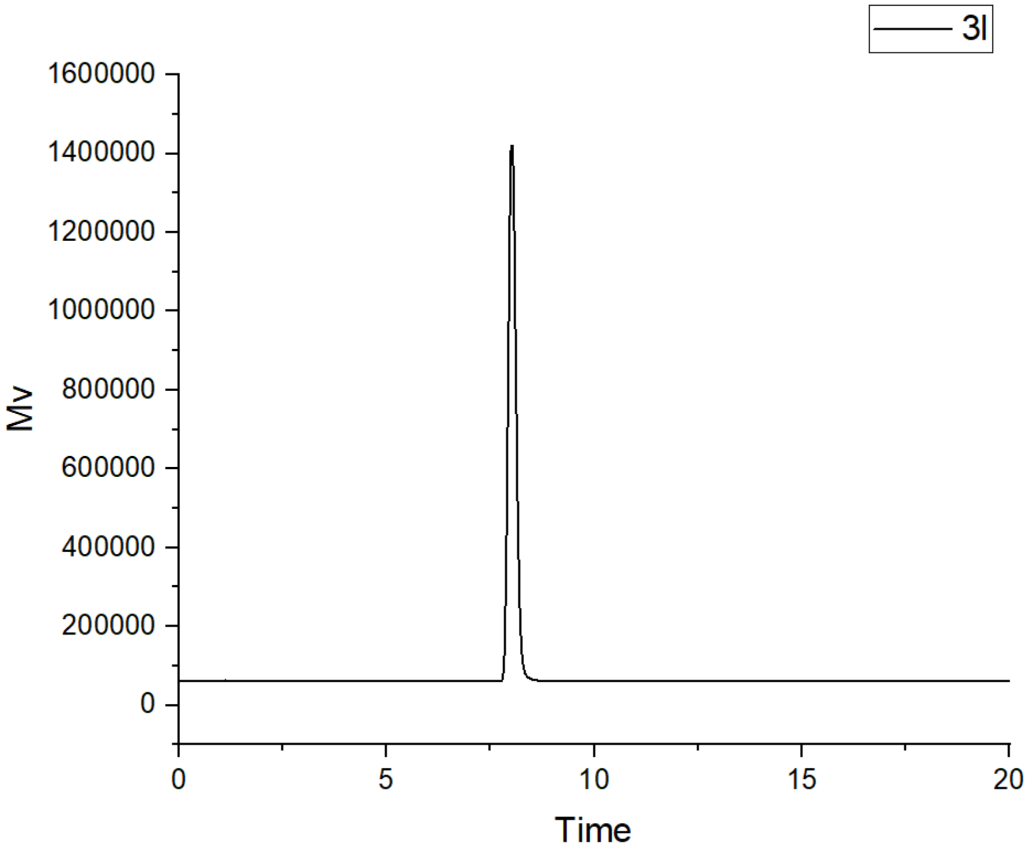
3l
1600000
1400000
1200000
1000000
800000
600000
400000
200000
0
Mv
0
5
10
15
20
Time

3m

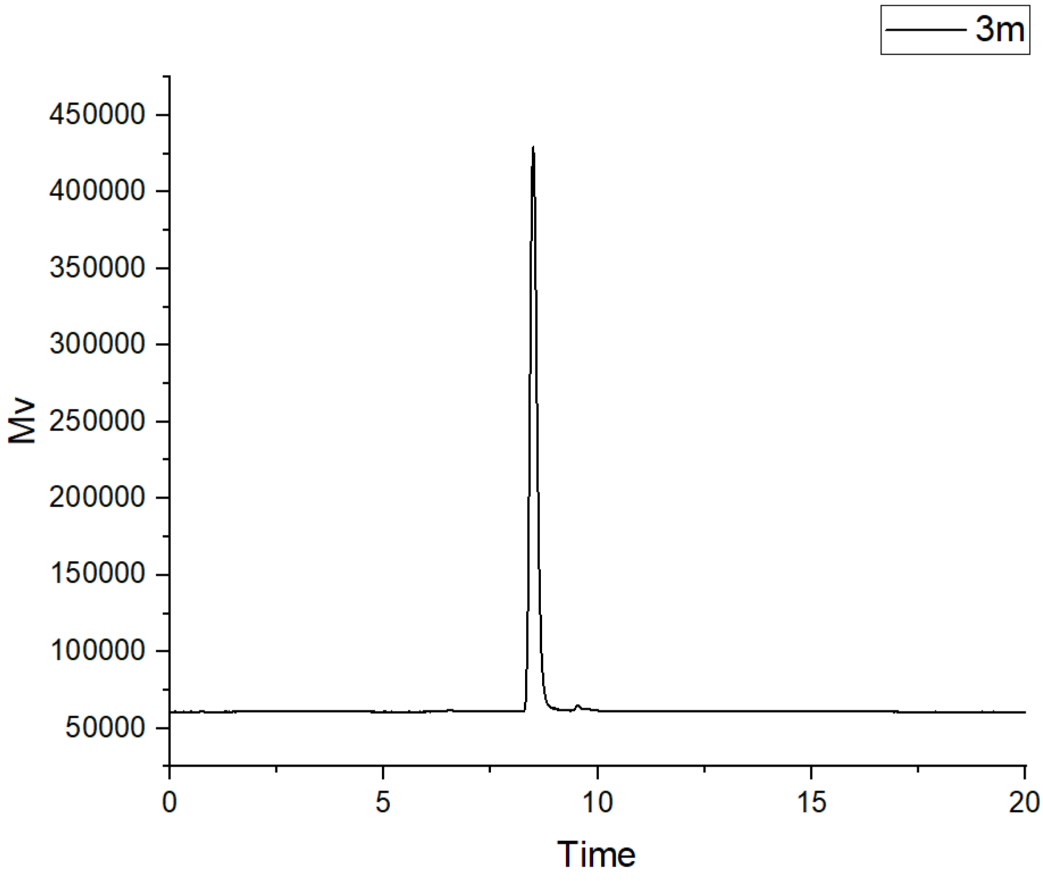
3m
450000
400000
350000
300000
250000
200000
150000
100000
50000
Mv
0
5
10
15
20
Time

3n

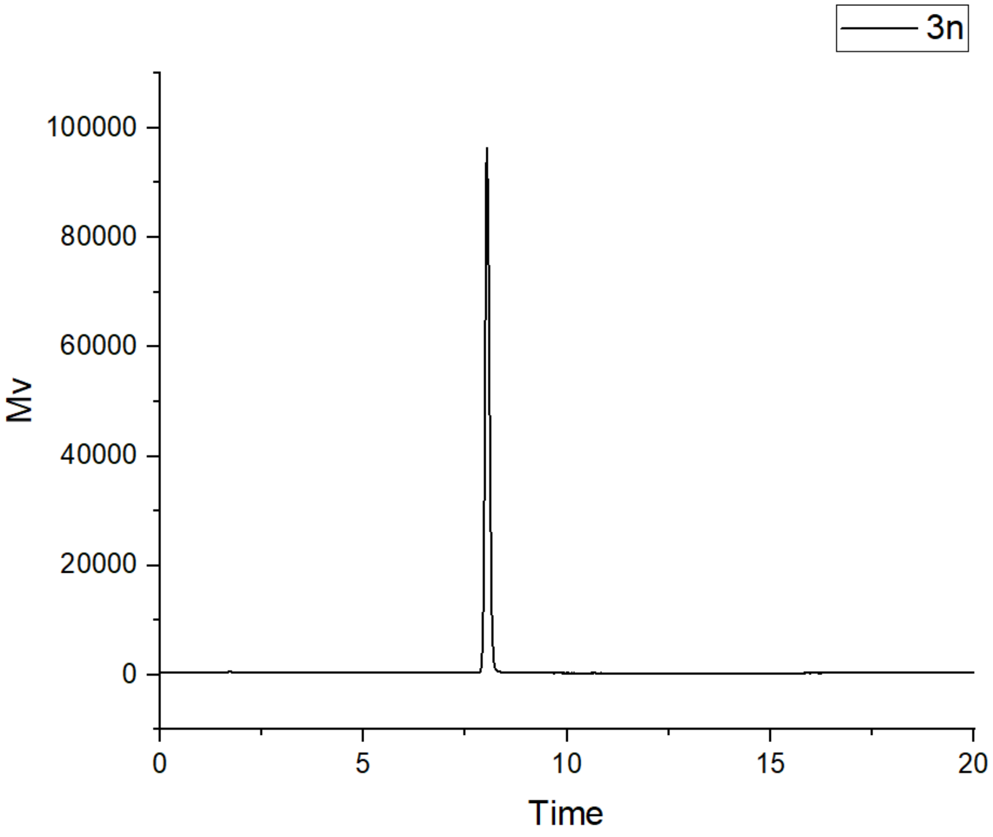
3n
100000
80000
60000
40000
20000
0
Mv
0
5
10
15
20
Time

3o

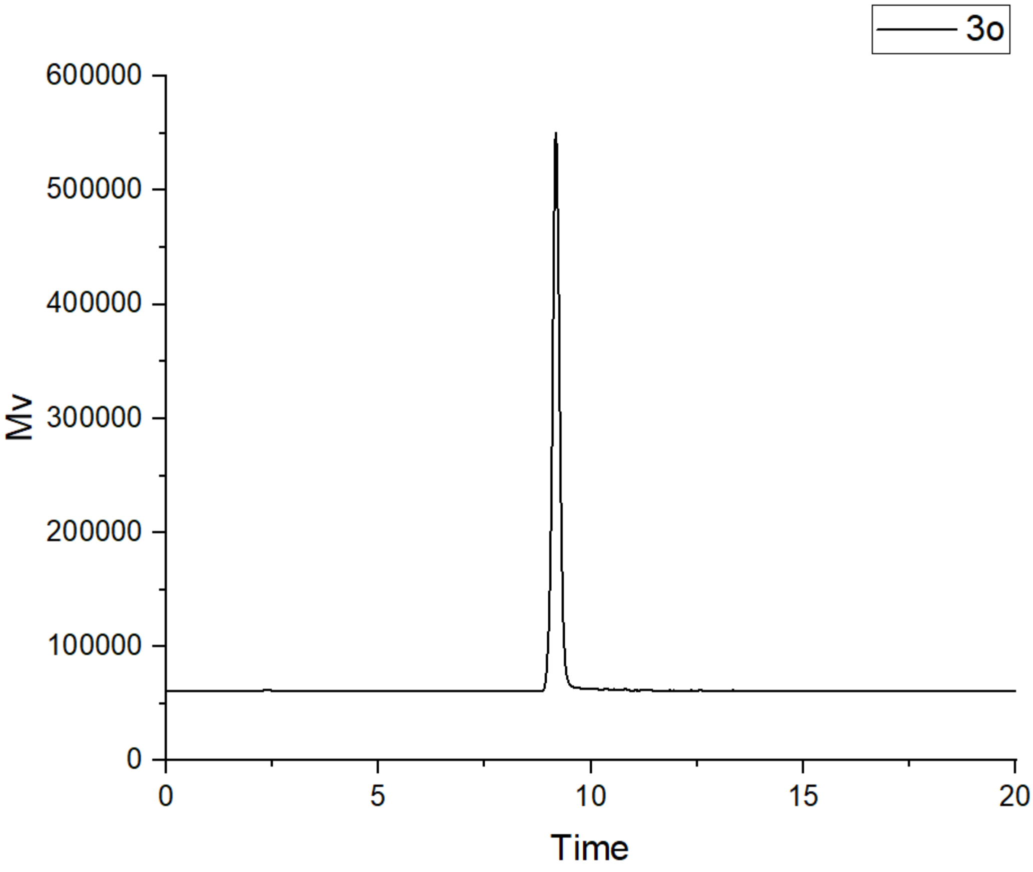
3o
600000
500000
400000
300000
200000
100000
0
Mv
0
5
10
15
20
Time

3p

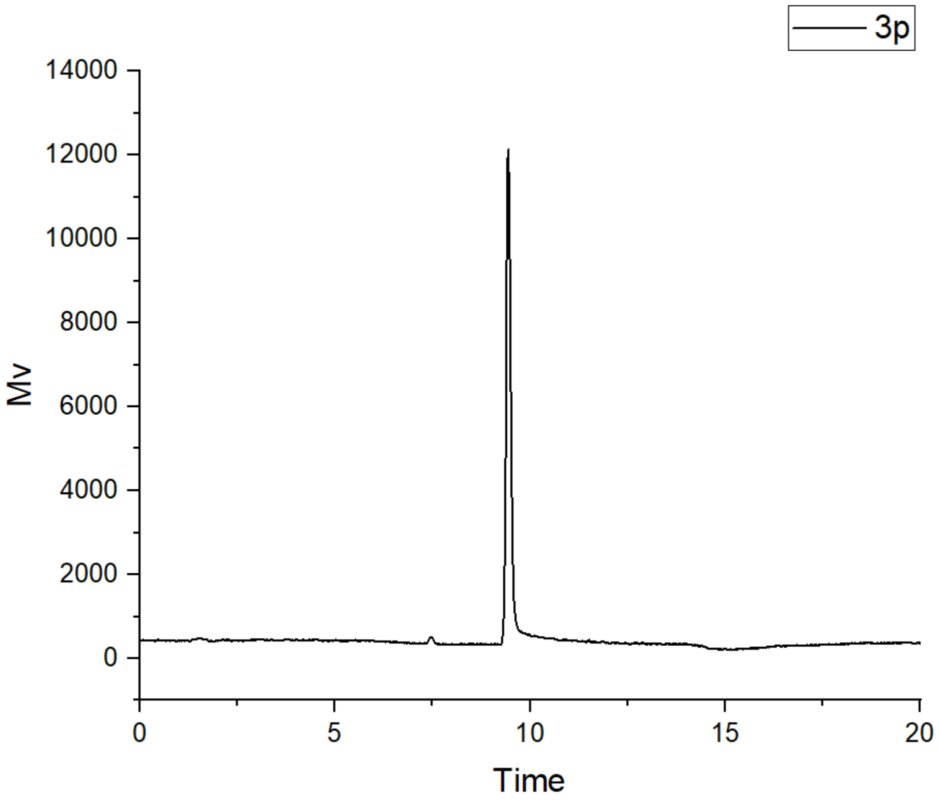
3p
14000
12000
10000
8000
6000
4000
2000
0
Mv
0
5
10
15
20
Time
